# Photoinduced, Chemoselective γ‐Alkylation of 2‐Silyloxyfurans With α‐Bromoketones: A Rapid Entry to Chiral ε‐Keto‐γ‐Butenolides

**DOI:** 10.1002/chem.202503083

**Published:** 2025-12-18

**Authors:** Debora Guazzetti, Luca Aimi, Enrico Marcantonio, Giovanni Maria Siciliano, Kelly Bugatti, Sara Dobani, Andrea Sartori, Lucia Battistini, Franca Zanardi, Claudio Curti

**Affiliations:** ^1^ Department of Food and Drug University of Parma Parma Italy; ^2^ Department of Chemistry Aarhus University Aarhus 8000 Denmark

**Keywords:** heterocycles, lactones, photoredox catalysis, principle of vinylogy, radicals

## Abstract

γ‐Butenolides are widespread structural motifs found in many natural and unnatural products which display an impressive range of biological activities. Among them, ε‐keto‐γ‐butenolides represent underestimated butenolide frameworks, which could serve as valuable platforms to build complex structures, for example, heterobicyclic derivatives. Quite unexpectedly, despite the apparent simplicity of their structures, efficient synthetic methodologies enabling the construction of chiral, ε‐keto‐γ‐butenolide architectures are quite underdeveloped. In this context, herein we present a novel, photoinduced regio‐ and chemoselective γ‐alkylation of 2‐silyloxyfurans with 2‐bromoketones providing a practical access to ε‐ketobutenolide scaffolds in racemic format, in one single step and high yields. The usefulness of these products as starting materials to build chiral, fused‐heterobicycle lactone derivatives was demonstrated by the implementation of a two‐step strategy which successfully delivered unprecedented phenyltetrahydrofuro[3,2‐*b*]furan‐2(3*H*)‐one and tetrahydrofuro[3,2‐*c*]pyridazin‐6(1*H*)‐one chemotypes.

## Graphical abstract

A photoinduced, regio‐ and chemoselective γ‐alkylation of 2‐silyloxyfurans with a‐bromoketones was achieved by exploiting a photoredox catalytic process promoted by blue light. This approach provides practical and efficient access to chiral, ε‐ketobutenolides, in one step and high yields. The usefulness of these scaffolds as versatile platforms to access complex, heterobicyclic molecules via late‐stage modifications was also demonstrated.

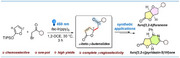

## 1 Introduction

**SCHEME 1:**
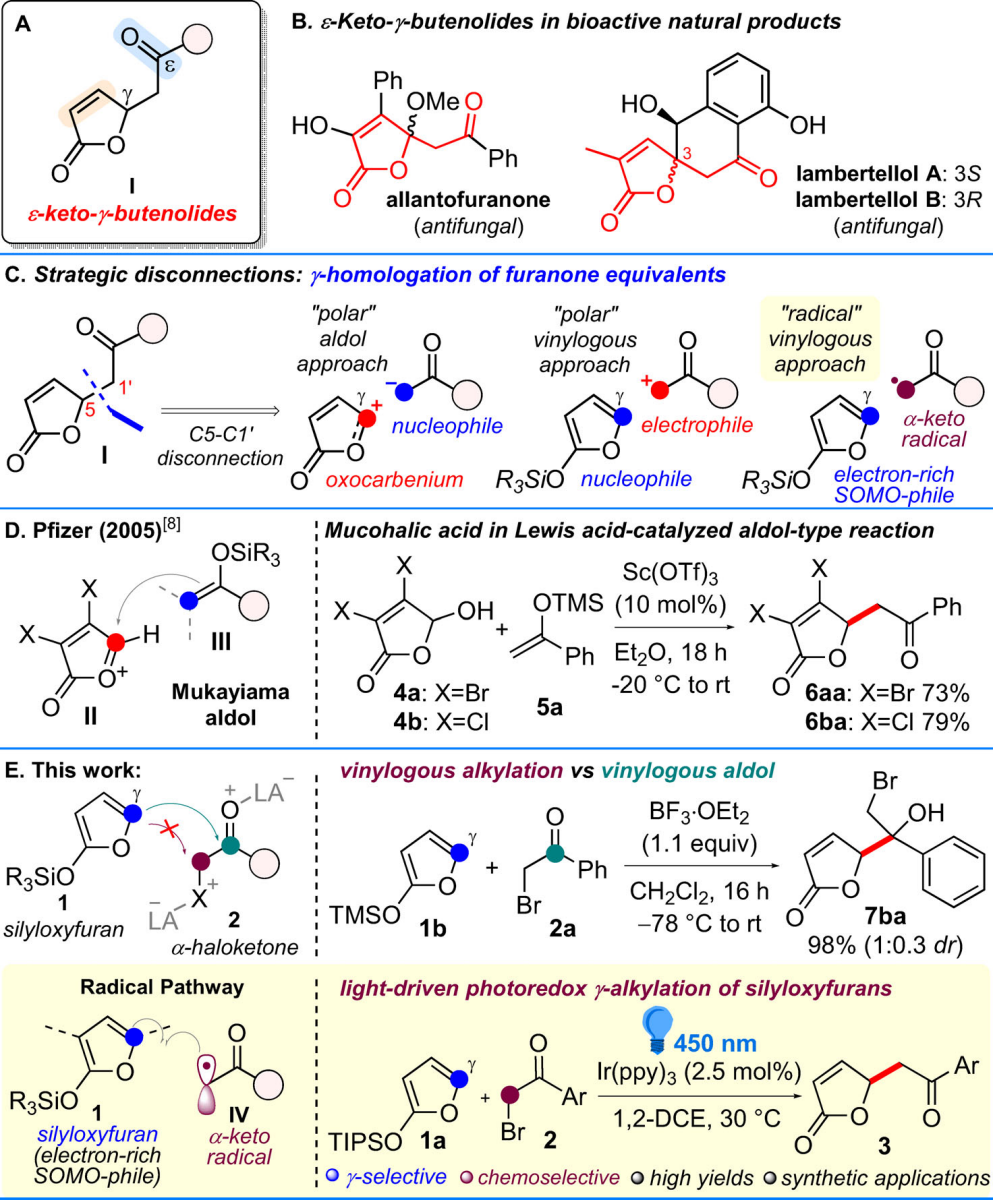
(A) General structure of the targeted ε‐keto‐γ‐butenolides of type **I**. (B) Examples of natural products embedding the ε‐keto‐γ‐butenolide framework. (C) Strategic C5‐C1′ disconnections toward **I**. (D) Aldol‐type approach by J. Zhang et al. at Pfizer (see Ref. [8]). (E) This work: preliminary investigation on the feasibility of the polar, vinylogous alkylation strategy (see Section 3 of the Supporting Information for details), and outline of the successful radical approach.

γ‐Butenolides—i.e., substituted furan‐2(5*H*)‐ones—are five‐membered unsaturated lactones which constitute the structural core of a wide array of natural and unnatural products displaying an impressive range of biological activities in the fields of pharmaceuticals, agrichemicals, and organic materials [1, 2]. A peculiar, yet underestimated butenolide subset comprises ε‐keto‐γ‐butenolides of type **I** (Scheme 1A) which feature an ε‐ketone side chain linked to the stereogenic C5(γ)‐position of the α,β‐unsaturated lactone moiety. Such chiral, tridentate electrophiles, apart from being pivotal “per se” in several bioactive natural products, as antifungal allantofuranone [3] and lambertellols A and B [4, 5] (Scheme 1B), might also serve as prominent platforms for late‐stage modifications to build novel, complex, heterocyclic derivatives (vide infra).

Quite unexpectedly though, efficient synthetic methodologies enabling the construction of these architectures are quite underdeveloped, still limited almost exclusively to phthalide derivatives [6, 7]. From a synthetic point of view, one of the most straightforward access to the ε‐keto‐γ‐butenolide framework entails the strategic disconnection at the C5‐C1′ bond of **I** which envisages the regioselective *γ*‐homologation of a furan‐2(5*H*)‐one equivalent with a coupling (aryl)acyl partner (Scheme 1C). In this context, a first elegant approach was reported by Pfizer in 2005 [8], involving the Lewis acid‐catalyzed, Mukaiyama aldol‐type reaction between mucohalic acids **4a** and **4b** and various silyl ketene acetals or silyl enol ethers (Scheme 1D). In that instance, treatment of **4a** and **4b** with Sc(OTf)_3_ (10 mol%) and the silyl enol ether from acetophenone **5a**, provided the halogenated 5‐phenacylmethyl γ‐butenolides **6aa** and **6ba** in good, isolated yields via formation of the strongly electrophilic oxocarbenium ion **II** at the C‐γ of the butenolide. A reversed option would entail the vinylogous γ‐alkylation of a silyloxyfuran of type **1** with α‐haloketones of type **2** as suitable alkylating agents (Scheme 1E). Interestingly, despite the apparent easiness of this strategy, the γ‐alkylation of vinylogous silyloxyfurans **1** via a S*_N_* 1 or S*_N_* 2 mechanism on haloketones of type **2** is still unprecedented. Indeed, the chemoselective substitution of a nucleophile to the C(*sp* ^3^)−X of **2** appears challenging, as its bidentate electrophilic nature makes the aldol addition at the carbonyl a viable, competitive option [9]. To shed light onto the feasibility of this transformation, in this work we preliminarily tested a series of Lewis acids to promote the vinylogous alkylation of trimethylsilyloxyfuran **1b** with phenacyl bromide **2a** (Scheme 1E; see also Table S1 in the Supporting Information) [10]. Under these conditions, we observed the formation of the sole aldol adduct **7ba**, instead of the expected alkylated product, which was detected but only in traces in the reaction crude mixtures. On this ground, having established the vinylogous aldol reactivity bias of **1** with **2** in the polar domain, we turned to evaluate the viability of this alkylation according to a radical approach. Radicals have emerged as a powerful tool in advanced organic synthesis, offering unique reaction mechanisms and a wide range of applications [11, 12]. In this context, an efficient and sustainable method to promote radical reactions is visible‐light photoredox catalysis, which has polarized widespread research interest owing to its attractive features such as mild conditions, excellent functional group tolerance, and high reactivity [13, 14, 15]. Considering the strategic disconnection described in Scheme 1C, α‐haloketones **2**, like phenacyl bromide **2a**, are well known radical precursors which enable the formation of the corresponding α‐keto radical **IV** intermediates by single electron transfer (SET) reduction of the C─Br bond. As an electrophilic radical, intermediate **IV** could readily couple with electron‐rich olefins (SOMO‐philes) like dienamines [16], silyl enol ethers [17, 18, 19], or their vinylogous π‐extended congeners [20]. We envisaged that, in principle, radical **IV** could likely be trapped by electron‐rich silyloxyfurans **1**, hence delivering the otherwise hardly accessible γ‐butenolides of type **3** (Scheme 1E, bottom). Continuing our efforts toward the discovery of novel synthetic methodologies, that would merge radical chemistry and the principle of vinylogy to expand the structural domain currently accessible through the common (poly)enolate chemistry [21], we here introduce a novel, chemoselective and γ‐regioselective methodology to afford ε‐keto‐γ‐butenolides **3**, in one single step and high efficiency. Furthermore, late‐stage functionalization of scaffolds **3** has been accomplished, demonstrating their usefulness as versatile platforms to readily access complex, heterobicyclic derivatives.

## 2 Results and Discussion

At the outset of our investigation, we selected triisopropylsilyloxyfuran (TIPSOF) **1a** and phenacyl bromide **2a** as model substrates to evaluate the proof‐of‐concept and to optimize reaction conditions (Table 1). Initially, we began our survey by testing conditions common to similar transformations [20], i.e., using *fac*‐Ir(ppy)_3_ (**Ir‐1**) as the photocatalyst (2.5 mol%), K_2_CO_3_ (2 mol equivalents) in degassed CHCl_3_ under blue LED irradiation (450 nm), at 30°C for 5 h. Under these conditions, **1a** reacted with excess **2a** (1.5 equiv) to produce the desired product **3aa** (35% yield after column chromatography), along with the unexpected γ,γ‐bis‐alkylated adduct **8aa** in a 1:0.17 mixture (Table 1, entry 1). Evaluation of several other metal‐ and organic photocatalysts did not improve the formation of **3aa**, with **Ir‐2** and **Ru‐1** being the only alternatives, although giving quite lower yields compared to **Ir‐1** (entries 2 and 3 and Table S2 in the Supporting Information). We then surveyed other solvents, identifying 1,2‐dichloroethane (1,2‐DCE) as a better alternative (entry 4), which provided **3aa** in a 42% isolated yield, accompanied by a lower amount of **8aa** (1:0.1 **3aa**:**8aa**).

**TABLE 1:** Optimization of reaction conditions (selected entries).^a^

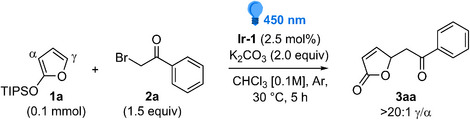			
Entry	Deviations from the initial conditions	Yield [%]^b^ **3aa**	**3aa**:**8aa** ^c^
1	None	35	1:0.17
2	**Ir‐2**	9	n.d.
3	**Ru‐1**	20	1: 0.08
4	1,2‐DCE	42	1:0.10
5	MeCN	*Degradation*	
6	1,2‐DCE, **1a** [0.06 M]	45	1:0.10
7	As entry 6 without K_2_CO_3_	50	1:0.06
8	As entry 7 with 1.5:1 (**1a**:**2a**)	90	1:0.03
9	As entry 8, for 3 h	93	1:0.03
10	As entry 9, with **1b** instead of **1a**	88	1:0.01
11	No photocatalyst	*No reaction*	
12	No light	*No reaction*	
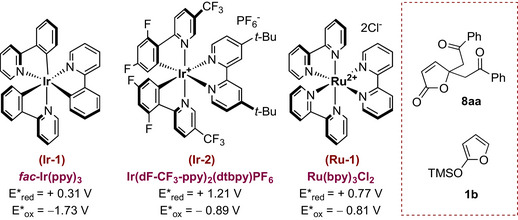			

^a^ Initial reaction conditions: **1a** (0.1 mmol), **2a** (0.15 mmol, 1.5 equiv), and **Ir‐1** (2.5 mol%) in degassed CH_3_Cl [0.1 M] at 30°C, irradiated with blue LEDs (450 nm) for 5 h.

^b^ Isolated yields of **3aa** after silica gel flash chromatography.

^c^ Determined by ^1^H NMR analysis of the crude. See Tables S1–S5 in the Supporting Information for the complete optimization survey. n.d. = not determined.

Envisaging that the formation of **8aa** could be fostered by the carbonate [22], we tested the reaction without the base in a more dilute solution (Table 1, entry 7); pleasingly, after 5 h at 30°C, **3aa** was isolated in an improved 50% yield, accompanied by a corresponding decrease of **8aa** (1:0.06 **3aa**:**8aa**). The real breakthrough occurred by switching the stoichiometric ratio between **1a** and **2a** to 1.5:1 (entries 8 and 9), ultimately providing **3aa** with an optimal 93% isolated yield after 3 h, with only traces of **8aa** (entry 9). Finally, the use of the less hindered trimethylsilyloxyfuran (TMSOF) **1b** proved to be almost equally productive as **1a**, providing **3aa** in a 88% isolated yield while reducing the generation of **8aa** (1:0.01 **3aa**:**8aa**, entry 10). Importantly, in all cases, full γ‐regioselectivity was observed, with no detection of α‐alkyl products. Likewise, no products deriving from attack to the ketone moiety were observed, testifying complete chemoselectivity. Finally, as evidence of its photocatalytic nature, the reaction was completely inert in the absence of either the photocatalyst (entry 11) or light (entry 12). Having established the optimal reaction conditions for accessing γ‐alkylated butenolide **3aa** from **1a** or **1b** (Table 1, entries 9 and 10), we proceeded to evaluate the scope and limitations of the process. As depicted in Table 2, a series of differently substituted aromatic and aliphatic α‐bromoketones **2a‐r** underwent the desired transformation furnishing the corresponding ε‐keto‐γ‐butenolides **3**.

**TABLE 2:** Substrate scope of the photoinduced γ‐alkylation of silyloxyfurans **1a‐d**.^a^, ^b^

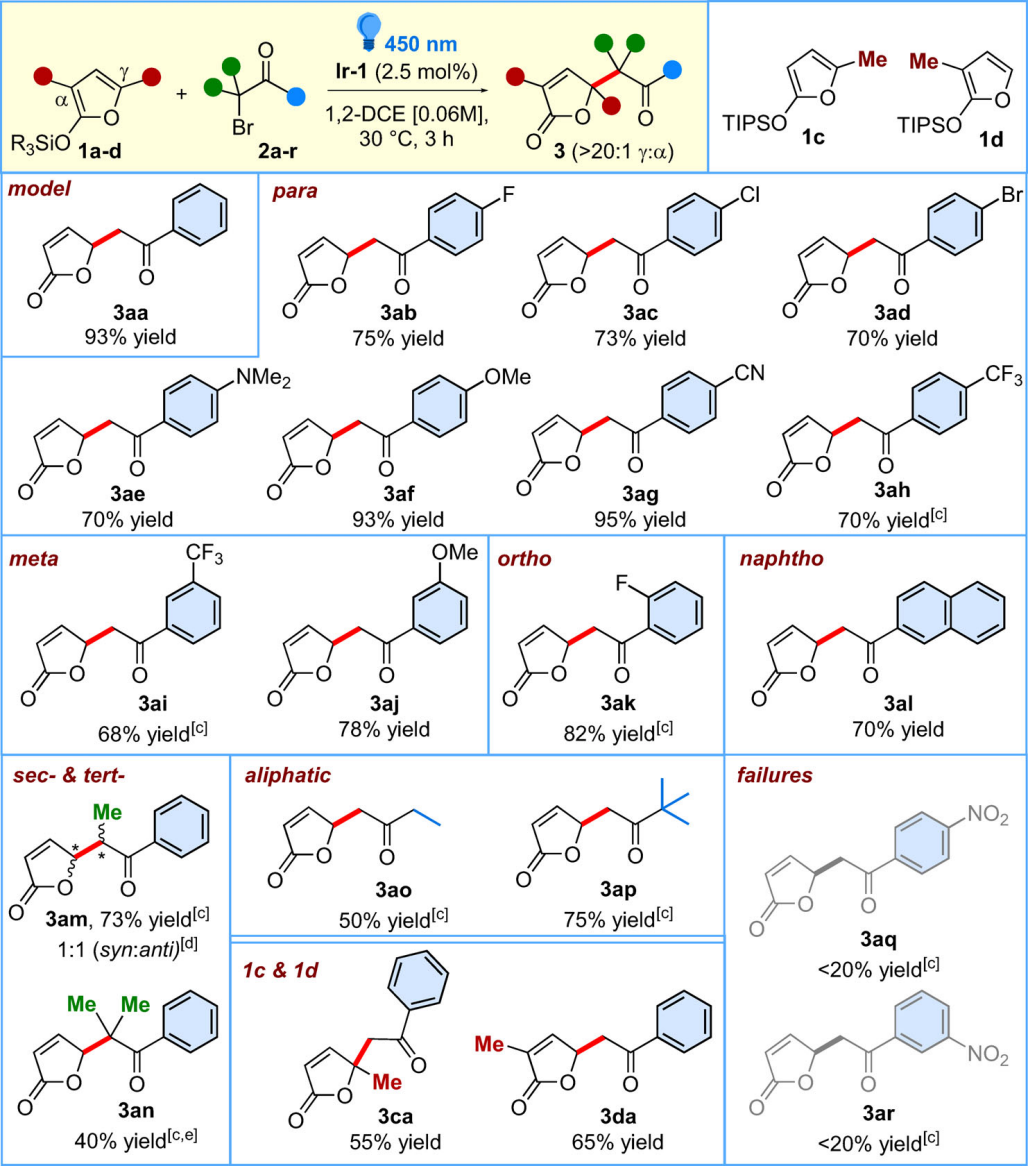

^a^ *Reaction conditions*: **1** (1.5 equiv), **2** (0.1 mmol, 1.0 equiv), **Ir‐1** (2.5 mol%) in degassed 1,2‐DCE [0.06 M] at 30°C, irradiated with blue LEDs (450 nm) for 3 h.

^b^ Yields refer to isolated yields of **3** after silica gel flash chromatography. In all entries, less than 5% of the corresponding γ,γ‐bisalkylated adduct **8** was detected in the crude by ^1^H NMR.

^c^ TMSOF **1b** was used instead of **1a**.

^d^ Determined by ^1^H‐NMR analysis of the crude.

^e^ Regioisomeric ratio: 1.3:1 γ/α, 70% combined yield. See the Supporting Information for details.

Concerning the reactivity of aromatic ketones (acetophenones) with TIPSOF **1a**, irrespective of the electron‐withdrawing or electron‐donating ability of the substituents on the aryl ring, the reaction proved viable under the optimized reaction conditions, as demonstrated, for example, by the electron‐rich 4‐methoxy congener **2f** and the electron‐poor 4‐cyano‐acetofenone **2g** which provided the best results, delivering the corresponding adducts **3af** and **3ag** in highly rewarding yields. Several compounds, including electron‐poor *ortho*‐fluoro‐acetophenone **2k** and *para*‐ and *meta*‐trifluoromethyl‐acetophenones **2h** and **2i** reacted better with the less hindered TMSOF **1b**, providing compound **3ak** in a good 82% isolated yield, and butenolides **3ah** and **3ai** in slightly lower yields around 70%. An exception was observed with electron‐deficient ketones **2q** and **2r**, bearing nitro groups at the *para*‐ and *meta*‐positions, respectively, which failed to provide the expected adducts in satisfactory yields (<20% isolated yield for both). In contrast, **1b** proved to be a competent substrate for sterically demanding secondary and tertiary bromides **2m** and **2n**. Under the optimized conditions, the prostereogenic acetophenone **2m** reacted with **1b** to furnish the corresponding γ‐adduct **3am** in a good 73% isolated yield as a 1:1 *syn*/*anti* mixture. An unusual outcome was observed with tertiary 2‐bromo‐2‐methyl‐acetophenone **2n**, which afforded a 1.3:1 γ/α regioisomeric mixture of products **3an** in a combined 70% yield (40% isolated yield for the sole γ‐adduct) [23, 24]. Finally, γ‐ and *α*‐methyl‐substituted silyloxyfurans **1c** and **1d** were also tested. Under the optimized reaction conditions, both scaffolds reacted with **2a** with similar efficiency, affording the corresponding *γ*‐adducts **3ca** and **3** **da** in 55% and 65% yields, respectively. As a proof of the generality of the disclosed procedure, the reaction also proceeded smoothly with aliphatic bromides such as 1‐bromo‐2‐butanone **2o** and 1‐bromopinacolone **2p**, which reacted with **1b** to deliver the γs‐adducts **3ao** and **3ap** in 50% and 75% isolated yields, respectively.

**SCHEME 2:**
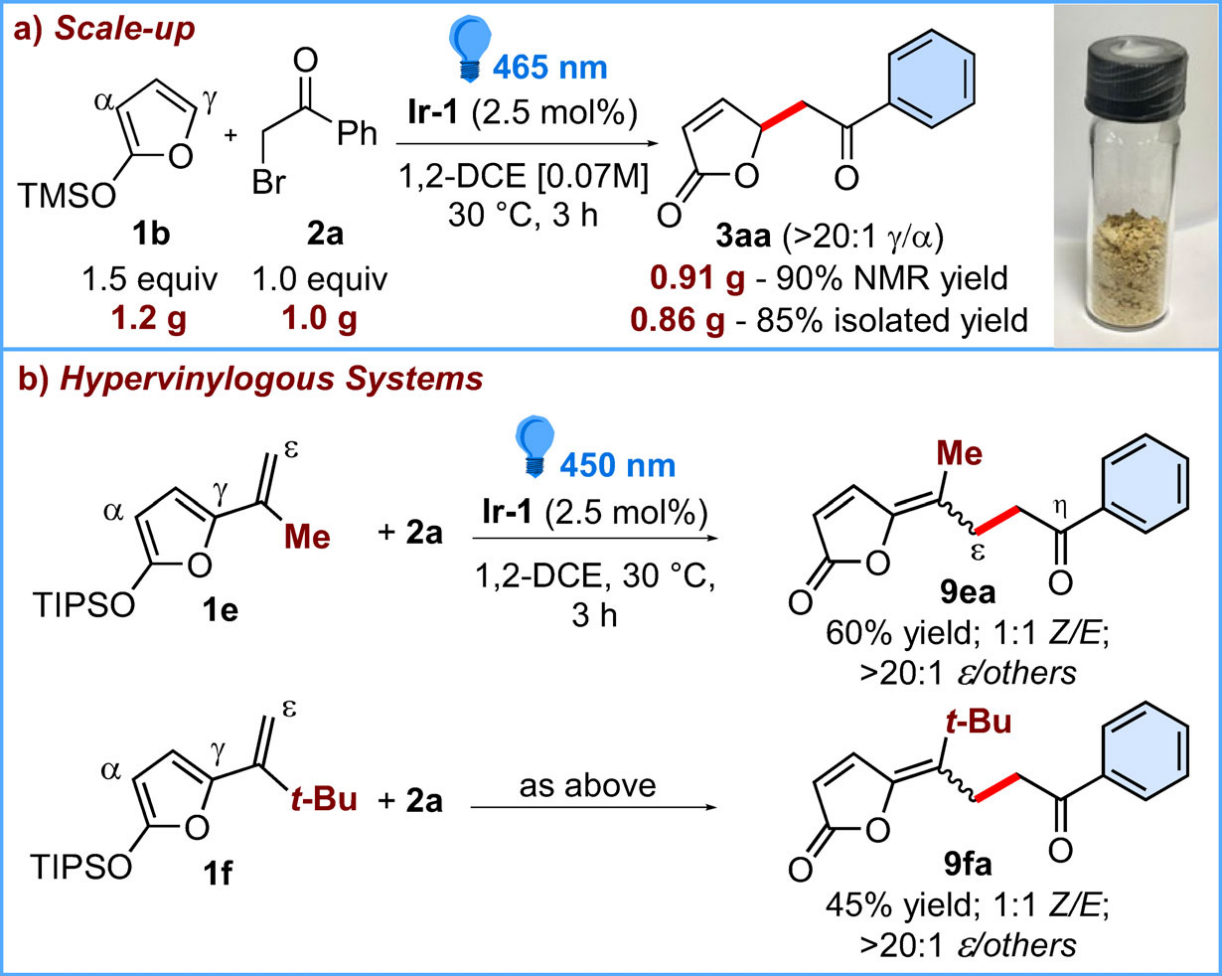
(a) Gram‐scale experiment conducted with a 465 nm blue LED strip source (14.4 W) (b) Photoinduced ε‐alkylation of extended silyloxyfurans **1e** and **1f**. *Reaction conditions*: **1** (1.5 equiv), **2a** (0.1 mmol, 1.0 equiv), **Ir‐1** (2.5 mol%) in degassed 1,2‐DCE [0.06 M] at 30°C, irradiated with blue LEDs (450 nm) for 3 h. Yields refer to isolated yields of **9** after silica gel flash chromatography. The *Z/E* ratio was determined by ^1^H NMR analysis of the crude. See the Supporting Information for details.

The reaction between **1b** and **2a** was successfully carried out also on a gram‐scale using 5.0 mmol of **2a**. Gladly, under slightly modified reaction conditions (i.e., 465 nm blue LED strip source, and 0.07 M instead of 0.06 M reaction concentration; see Section 4.4 of the Supporting Information for details), pure **3aa** was obtained (0.86 g, 85% isolated yield) without any loss of regioselectivity (Scheme 2a). Of note, subjecting the reaction mixture to vacuum‐driven evaporation for 12 h, provided a crude with the sole **3aa** in a 90% NMR purity (0.91 g, 90% yield). As a further extension of the scope, the disclosed methodology could also be employed to regioselectively functionalize more extended π‐systems, alias hypervinylogous systems [25], such as electron‐rich, methyl‐ and *tert*‐butyl‐substituted γ‐vinyl silyloxyfurans **1e** and **1f** (Scheme 2b). Under the optimized reaction conditions, both trienes were able to trap the α‐ketoradical derived from **2a** selectively at the most distal ε‐site, providing η‐keto‐γ‐vinylidene butenolides **9ea** and **9fa** as 1:1 mixture of the corresponding *Z*/*E* isomers in 60% and 45% combined yields, respectively.

**SCHEME 3:**
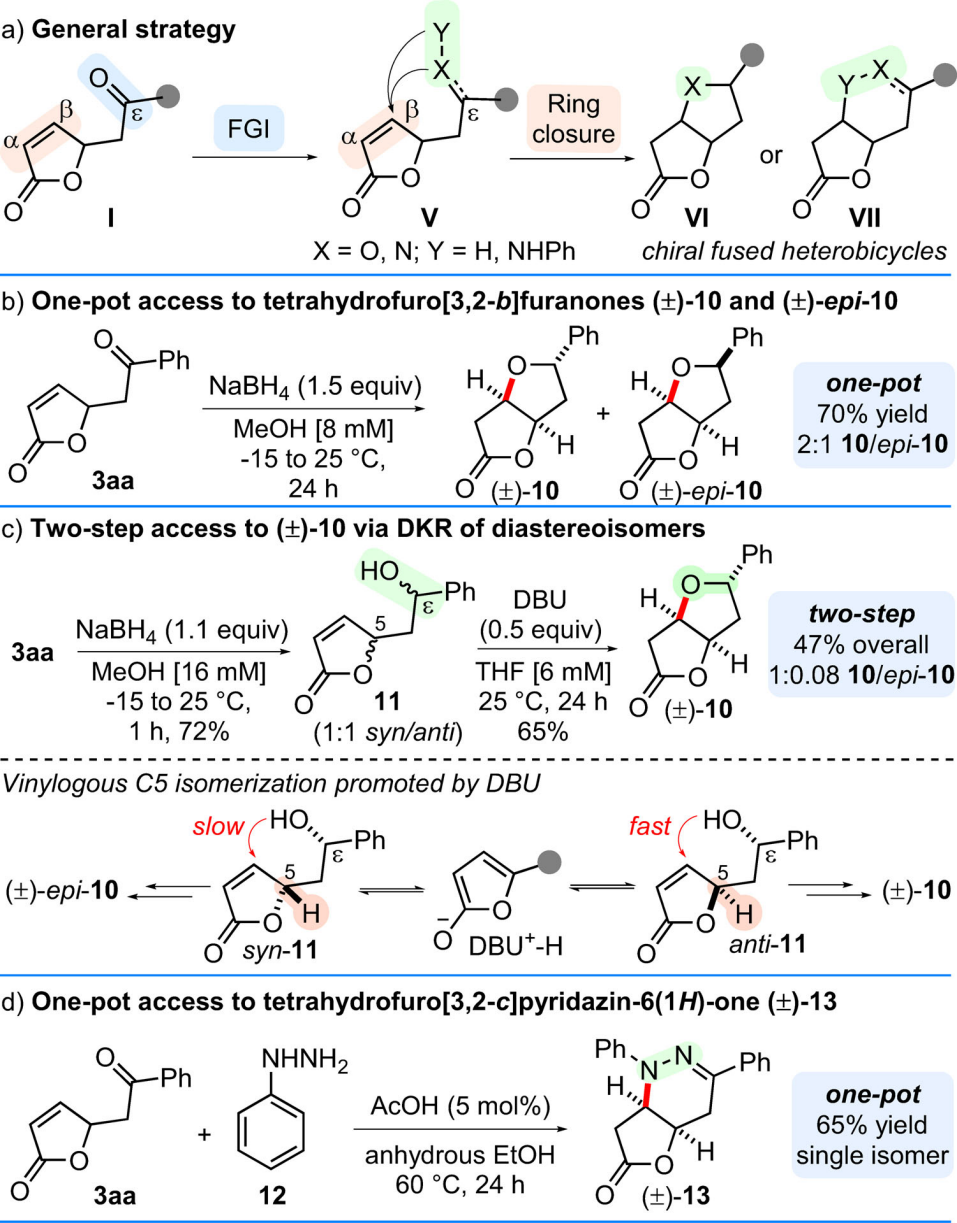
(a) General strategy for late‐stage modification of butenolides **I**. (b) One‐pot access to bicyclic furanones (±)‐**10** and (±)‐*epi*‐**10**. (c) Two‐step access to (±)‐**10** via dynamic kinetic resolution (DKR) of diastereomers *syn*‐**11** and *anti*‐**11**. (d) One‐pot access to bicyclic pyridazine‐6(1*H*)‐one (±)‐**13**. In all cases yields refer to isolated yields; the ratio **10**/*epi*‐**10** was determined by ^1^H NMR of the crude. See the Supporting Information for details.

Having established an efficient procedure for accessing chiral *ε*‐keto‐*γ*‐butenolide chemotypes of type **3**, we then moved on to demonstrate their versatility as strategic platforms to forge fused heterobicyclic scaffolds such as tetrahydrofuro[3,2‐*b*]furan‐2(3*H*)‐ones of type **10** [26] (Scheme 3b‐c), and the tetrahydrofuro[3,2‐*c*]pyridazine‐6(1*H*)‐one **13** (Scheme 3d) [27, 28, 29]. To this end, we envisaged a two‐step strategy centered upon the interconversion of the ε‐ketone moiety of **I** (*e.g*. by reduction to secondary alcohol or hydrazone formation), followed by ring closure at the β‐position of the electron‐deficient unsaturated lactone (Scheme 3a).

Indeed, treating **3aa** with slight excess NaBH_4_ in MeOH (at −15°C to 25°C for 24 h) failed to deliver the expected open‐chain alcohol, ending up in a 2:1 mixture of the *cis*‐fused dioxabicycles (±)‐**10** and (±)‐*epi*‐**10**, in a good 70% combined, isolated yield (Scheme 3b). The easiness of this one‐pot, ketone reduction/*oxa*‐Michael cyclization cascade prompted us to investigate a more diastereoselective procedure to access either (±)‐**10** or (±)‐*epi*‐**10**. After a brief survey (see Table S8 in the Supporting Information for details), we found that treatment of **3aa** with NaBH_4_ (1.1 equiv) in a more concentrated MeOH solution [16 mM], for 1 h, produced alcohol **11** as a racemic 1:1 *syn*/*anti* mixture in a combined 72% yield. Quite unexpectedly though, treating this equimolar mixture of diastereomers with a sub‐stoichiometric amount of DBU (0.5 equiv) in THF under dilute conditions [6 mM] enabled the formation of 5‐phenyltetrahydrofuro[3,2‐*b*]furan‐2(3*H*)‐one (±)‐**10** in a good 65% isolated yield (47% overall) as an almost single isomer (1:0.08 **10**/*epi*‐**10**, Scheme 3c). This result is far from being trivial, since it implies the diastereoselective cycloetherification of the 1:1 *syn*/*anti* mixture of alcohol **11** via a peculiar dynamic kinetic resolution (DKR) [30, 31, 32]. Such DKR is likely enabled by the DBU‐catalyzed, vinylogous C5 site epimerization [33] of *syn*‐**11** into *anti*‐**11**, which cyclizes more rapidly than its isomer, thus selectively leading to (±)‐**10** (Scheme 3c). An *aza*‐version strategy was also implemented, involving the formation of a hydrazone and its subsequent intramolecular *aza*‐Michael ring closure. Indeed, treating **3aa** with phenylhydrazine (**12**) in the presence of a catalytic amount of glacial acetic acid (Scheme 3d) [17] directly produced a single, heterobicyclic compound, namely 1,3‐diphenyl‐4,4a,7,7a‐tetrahydrofuro[3,2‐*c*]pyridazin‐6(1*H*)‐one (±)‐**13** in one‐pot and in a good 65% isolated yield (Scheme 3d).

**SCHEME 4:**
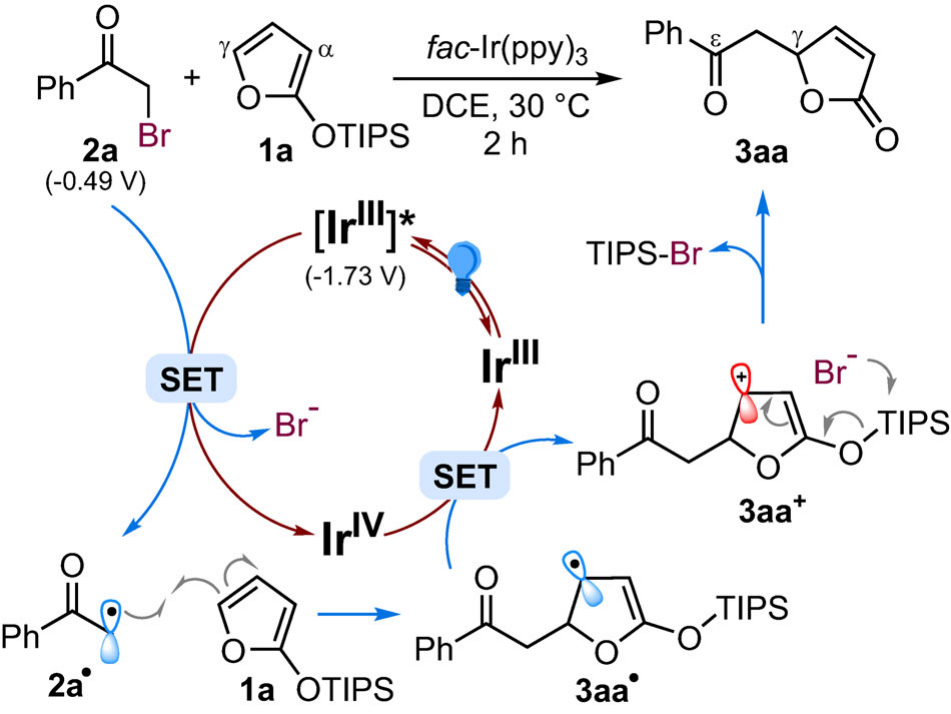
Proposed catalytic cycle.

Based on previous studies highlighting the ability of electron‐rich polyenes to trap electrophilic radicals at distal positions [20, 34, 35, 36], and a series of control proofs such as TEMPO trapping, and On‐Off experiments (see Sections 6.1 and 6.2 in the Supporting Information for details), a plausible catalytic cycle for the photoredox γ‐alkylation of 2‐silyloxyfurans is proposed, as exemplified by the addition of **2a** to **1a** (Scheme 4). The reaction is initiated with the well‐known single‐electron reduction of phenacyl bromide **2a** [*E*_ox_ = −0.49 V vs. Ag/AgCl] [34, 37] by the excited state photocatalyst *Ir^III^(ppy)_3_ [*E*_ox_ = −1.73 V vs. SCE] [15] which affords the corresponding α‐keto radical **2a** ^•^.

Such an electrophilic radical is then selectively trapped at the γ‐position of the electron‐rich diene **1a**, forming the radical intermediate **3aa** ^•^, which is subsequently oxidized to the corresponding carbocation **3aa^+^** via reduction of the newly formed Ir^IV^(ppy)_3_ [*E*_red_ = +0.77 V vs. SCE] [15] hence closing the photocatalytic cycle. Finally, desilylation of **3aa^+^** by the bromide anion results in the formation of the observed adduct **3aa** [22].

The pronounced γ‐regioselectivity observed in nearly all reactions examined in this study highlights the strong bias of electron‐rich polyenes such as **1a** and **1b** to preferentially “trap” electrophilic radicals at remote positions [20, 35, 36]. From a theoretical perspective, this site selectivity is likely governed by two factors: (i) a favorable overlap between the HOMO of the diene system **1** and the SOMO of the electrophilic radical **2^•^**, and (ii) the formation of a stabilized oxyallyl radical intermediate **3aa^•^** (Scheme 4), which is uniquely accessible through the γ‐addition pathway. As a support of this rationale, we took into consideration the DFT calculations performed in a very recent work, aiming at elucidation of the electronic distribution of **1a** and **1b**, based on HOMO composition and global/local electrophilicity and nucleophilicity indices [21]. In that study, the neutral, closed‐shell **1a** exhibited a pronounced nucleophilic character, consistent with its ability to trap electrophilic radicals and carbocations. Both HOMO orbital analysis and condensed Fukui indices (f^−^) confirmed a strong preference for γ‐addition, a trend also observed for **1b**.

## 3 Conclusion

In conclusion, we have successfully developed a light‐mediated, *γ*‐regioselective alkylation of 2‐silyloxyfurans **1** with α‐bromoketones **2**, by exploiting a photoredox catalytic cycle promoted by blue light. This approach provides practical and efficient access to chiral, ε‐ketobutenolides of type **3**, in one single step, with high efficiency and complete γ‐regiocontrol. The usefulness of these scaffolds as versatile platforms to access complex, heterobicyclic derivatives was also demonstrated. Building on our investigations on the reactivity of silyloxyfurans in the field of vinylogous, photo‐promoted radical reactions, we anticipate that this methodology will inspire future discoveries for the direct and remote functionalization of the butenolide core in natural products and medicinal chemistry. Indeed, as this work demonstrates, the combination of visible light‐mediated radical chemistry and the peculiar reactivity of vinylogous systems could be used to promote elusive transformations not accessible via conventional “polar” pathways, enabling access to novel chiral chemotypes. Further investigations aimed at implementing enantioselective pathways for similar transformations, and the evaluation of the antiviral activity of heterobicycles (±)‐**10**, (±)‐*epi*‐**10** and (±)‐**13** are currently ongoing in our laboratory.

## Conflicts of Interest

The authors declare no conflicts of interest.

## Supporting information




**Supporting File 1**: The authors have cited additional references within the Supporting Information.

**Supporting File 2**: chem70577‐sup‐0002‐DataFile.pdf

## Data Availability

The data that support the findings of this study are avilable in the supplementary material of this article.
