# Supplementary material for: Photoinduced, Chemoselective γ‐Alkylation of 2‐Silyloxyfurans With α‐Bromoketones: A Rapid Entry to Chiral ε‐Keto‐γ‐Butenolides
Source: Chemistry. 2025 Dec 18;32(5):e03083. doi: 10.1002/chem.202503083 (PMC12865137; doi:10.1002/chem.202503083)
Supplement: Supplementary file 1 — Supporting File 1: The authors have cited additional references within the Supporting Information. [file CHEM-32-e03083-s001.pdf]

Electronic Supporting Information
Part 1

Photoinduced, Chemoselective γ-Alkylation of 2-Silyloxyfurans with α-Bromoketones: a Rapid Entry to Chiral ε-Keto-γ-Butenolides

Debora Guazzetti,† [a] Luca Aimi,† [a] Enrico Marcantonio,[a,b] Giovanni Maria Siciliano,[a] Kelly Bugatti,[a] Sara Dobani,[c] Andrea Sartori,[a] Lucia Battistini,[a] Franca Zanardi,*[a] and Claudio Curti*[a]

[a] D. Guazzetti, L. Aimi, Dr. E. Marcantonio, G. M. Siciliano, Dr. K. Bugatti, Prof. Dr. A. Sartori, Prof. Dr. L. Battistini, Prof. Dr. F. Zanardi, Prof. C. Curti
Department of Food and Drug,
University of Parma,
Parco Area delle Scienze 27A, I-43124 Parma, Italy
E-mail: claudio.curti@unipr.it, franca.zanardi@unipr.it

[b] Dr. E. Marcantonio (current address)
Department of Chemistry,
Aarhus University,
8000 Aarhus, Denmark

[c] Dr. S. Dobani
Department of Food and Drug,
University of Parma,
Via Volturno 39, I-43125 Parma, Italy

Table of Contents

Part 1

- Table of Contents S2
- 1. General Experimental S3
- 2. Starting Materials S4
  - 2.1 Photocatalysts (Figure S1) S4
  - 2.2 Silyloxyfurans **1a**-**1g** (Figure S2) S4
  - 2.3 α-Bromoketones **2a**-**2r** (Figure S3) S5
- 3. Vinylogous Alkylation of 2-Silyloxyfurans **1** with 2-Bromoacetophenone **2a** Promoted by Lewis Acids (Table S1) S6
  - 3.1 Preparation of (±)-5-(2-bromo-1-hydroxy-1-phenylethyl)furan-2(5*H*)-one (**7ba**) S6

4. Photoinduced γ-Alkylation of 2-Silyloxyfurans with 2-Bromoacetophenones S8
  - 4.1 Optimization studies (Tables S2-S6) S8
  - 4.2 Reaction monitoring via ^{1}H-NMR analyses of the crudes (Figure S4) S12
  - 4.3 Representative procedure A (Table S5, entry 9) S13
    - 4.3.1 Preparation of (±)-5-(2-oxo-2-phenylethyl)furan-2(5*H*)-one (**3aa**, Figure S5) S13
  - 4.4 Scale-up procedure to access **3aa** (Figure S6) S15
  - 4.5 Substrate scope (Table S7) S16
  - 4.6 Photoinduced ε-alkylation of extended silyloxyfurans **1e** and **1f** (Scheme S1) S33

5. Late-Stage Functionalization of Butenolide **3aa** (Scheme S2) S36
  - 5.1 One-pot access to tetrahydrofuro[3,2-*b*]-furanones (±)-**10** and (±)-*epi*-**10** (Table S8) S37
  - 5.2 Two-step access to tetrahydrofuro[3,2-*b*]-furanone (±)-**10** via oxa-Michael cyclization of alcohol (±)-**11** (Table S9) S42
  - 5.3 One-pot access to tetrahydrofuro[3,2-*c*]pyridazine-6(1*H*)-one (±)-**13** S43

6. Control Experiments S45
  - 6.1 Control experiments with TEMPO (Table S10) S45
  - 6.2 On-Off Experiment (Table S11, Figure S5) S46

7. Proposed Mechanism to Access bis-Alkylated Adduct **8aa** (Scheme S3) S48

8. References S49

1. General Experimental

- Unless otherwise noted, all photoinduced reactions were performed in 4 mL vials on a Merck Penn PhD Photoreactor m2 equipped with a 450 nm LED Light Module (Z744033) used with a 50% intensity for the reported times (h). Under these conditions, the reaction temperature within the reaction vessel was measured to be comprised between 30 and 35 ºC and the reaction, equipped with a magnetic stir bar, was stirred with a 200-rpm frequency.
- All solvents used in the photocatalyzed reactions, and particularly 1,2-dichloroethane (anhydrous 99.8%) were degassed using the freeze-pump-thaw method and then placed in a collection flask where they underwent a second degassing phase through argon bubbling.
- Air-sensitive reagents and solutions were transferred via syringe or cannula and were introduced to the apparatus through rubber septa. Acetonitrile (ACN) was HPLC grade and kept under activated 4Å molecular sieves. Solvents for chromatography and filtration including hexane, petroleum ether (bp≥90% 40-60 °C), and ethyl acetate, were ACS grade and were used as such without further purifications.
- Analytical thin layer chromatography (TLC) was performed on silica gel 60 F254 pre-coated plates with visualization under short-wavelength UV light and by dipping the plates with molybdate reagent (aqueous H_2SO_4 solution of ceric sulphate/ammonium molybdate) followed by heating.
- Flash column chromatography was performed using 40-63 μm or 63-200 μm silica gel using the indicated mixture of solvents.
- NMR spectra were recorded at 400 or 600 MHz (^{1}H) and 100 or 150 MHz (^{13}C). Spectra were referenced to tetramethylsilane (0.0 ppm, ^{1}H; 0.0 ppm, ^{13}C, in $CDCl_3$). Chemical shifts (δ) are reported in parts per million (ppm), and multiplicities are indicated as s (singlet), d (doublet), t (triplet), q (quartet), sext (sextet), sept (septet), dd (double doublet), m (multiplet), and b (broad). Coupling constants, *J*, are reported in Hertz. ^{1}H and ^{13}C NMR assignments are corroborated by 1D and 2D experiments (^{13}C-APT, ^{13}C-DEPT, gCOSY, gHSQC). In ^{13}C-DEPTq-135 NMR experiments, CH and CH_3 signals are pointed up, whilst quaternary carbons and CH_2 are pointed down.
- Melting Points (m.p.) were measured with a differential scanning calorimeter DSC 821e Mettler Toledo AG and are uncorrected.
- ESI-mass spectra were recorded on API 150EX apparatus and are reported in the form of (*m/z*).
- High-resolution mass analyses were performed using a Q-ToF Synapt XS high-resolution mass spectrometer interfaced with a UPLC system. The column C18 1.7 µm, 2.1 mm and 100 mm column, set at 40°C, was used for the chromatographic analysis. For the analysis gradient, eluent A was water acidified with 0,01% (v/v) FA and eluent B was acetonitrile acidified with 0,01% (v/v) FA. The percentage of eluent B was as follows: 7% at the baseline; 15% at 3 minutes; 30% at 7.5 minutes; 40% at 8 minutes; 95% at 8.5 minutes; 95% at 10 minutes; 7% at 10.2 minutes; 7% at 11.5 minutes; with a constant flow of 0.4 mL/min. A total amount of 5 µL of each sample was injected. Electrospray source (ESI) set in positive (ESI+) ionization mode was used for the MSe analysis. Settings were as follows: cone voltage of 30 V, cone gas flow of 50 L/h, capillary voltage of 3 kV, desolvation temperature of 600 °C, source temperature of 120 °C, desolvation gas flow of 600 L/h. The mass scan was set in a range between 50 and 600 amu in 0.2 sec for both ionization modes. For mass accuracy, leucine enkephalin was used as lock-mass.

2. Starting Materials

2.1 Photocatalysts

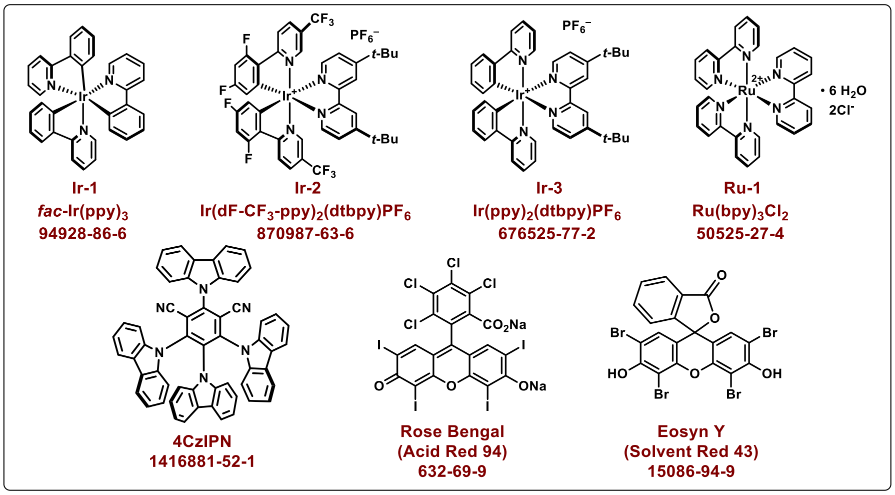

Figure S1. Structures, acronyms and CAS numbers of the photoredox catalysts screened in this work.

All photocatalysts depicted in Figure S1, which were used in this work, were commercially available, and were used as such, without further purification.

2.2 Silyloxyfurans 1a-1g

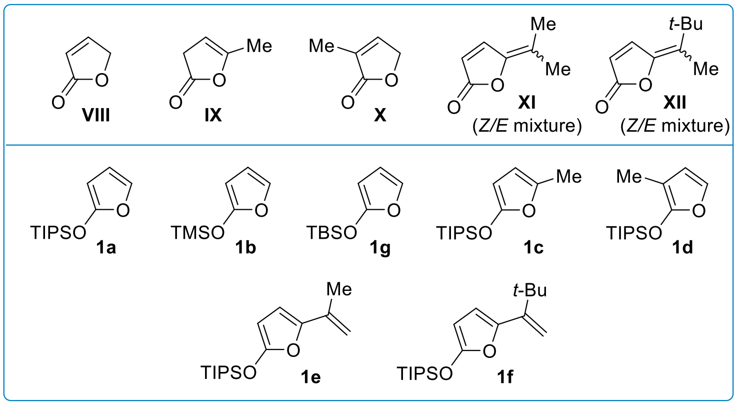

Figure S2. Structure of Silyloxyfurans **1a-g** and their furanone precursors **V-IX**.

2-Sililoxyfuran TIPSOF **1a**,[37] and TBSOF **1g**[38] were prepared from commercially available furan-2(5*H*)-one (**VIII**) according to literature procedures. 2-Trimethylsilyloxyfuran **1b** (TMSOF, 61550-02-5) was commercially available, and was used as such, without further purifications. Triisopropyl((5-methylfuran-2-yl)oxy)silane **1c** was prepared from commercial α-angelicalactone (**IX**) according to literature procedure.[37] Triisopropyl((3-methylfuran-2-yl)oxy)silane **1d** was prepared from commercial 3-methylfuran-2(5*H*)-one (**X**) according to literature procedure.[37] Silyloxytrienes **1e** and **1f** were prepared from the corresponding 5-alkylidene butanolide precursors **XI** and **XII** according to literature procedure.[24]Analytical and Spectroscopic data for **1a-1g** fully matched those reported in literature.

2.3 α-Bromoketones 2a-2r

α-Bromoketones **2a**-**2r** were all commercially available and were used as such without further purifications.

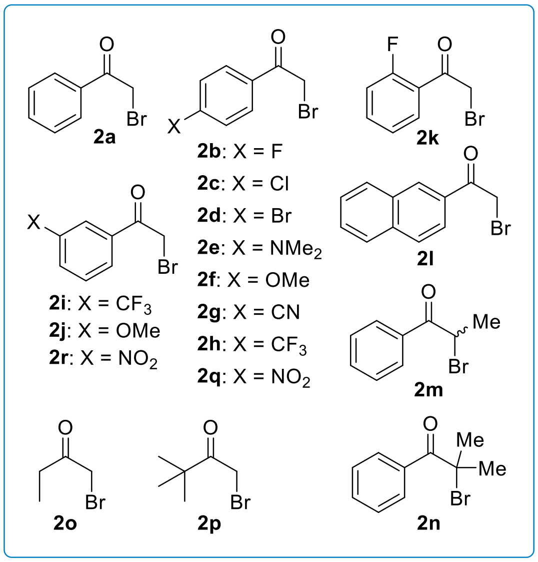

Figure S3. Structure of α-bromoketones **2a-r** used as radical precursors.

3. Vinylogous Alkylation of 2-Silyloxyfurans 1 with 2-Bromoacetophenone 2a Promoted by Lewis Acids

Table S1. Preliminary evaluation of vinylogous aldol *vs* alkylation reactivity of **2a** with **1**.

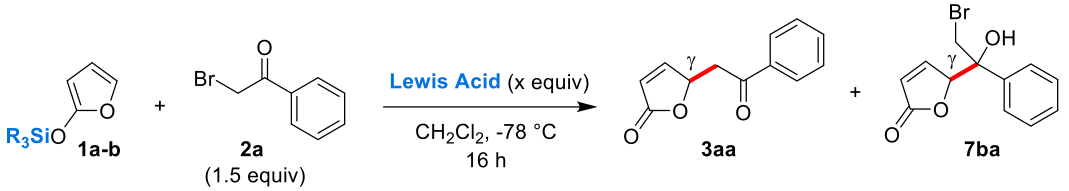

Entry	R_3Si (1)	Lewis Acid (equiv)	3aa:7ba[a]	d.r. (7ba)[b]	Yield % (7ba)[c]
1	TMS (**1b**)	AgOTf (0.5)	0.1:1	nd	<10
2	TIPS (**1a**)	AgOTf (0.5)	0.1:1	nd	<10
3	TMS (**1b**)	$Ag(CF_3CO_2)$ (1.1)	0.2:1	1:0.3	20
4[d]	TMS (**1b**)	BF_3•OEt_2 (1.1)	0.05:1	1:0.3	98

[a] Determined by ^{1}H NMR of the crude.
[b] Relative *syn*/*anti* configuration of **7ba** was not determined.
[c] Isolated, combined yield of **7ba**.
[d] Reaction carried out at –78 °C to rt in 16 h. nd = data not determined.

3.1 Preparation of *syn* and *anti* (±)-5-(2-bromo-1-hydroxy-1-phenylethyl)furan-2(5*H*)-one (7ba)

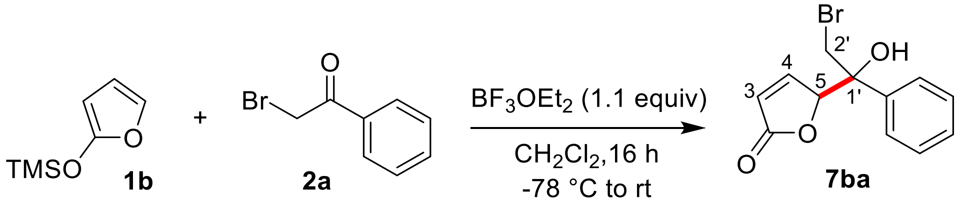

In an oven-dried 10 mL round-bottom flask equipped with a magnetic stir bar and kept under a stream of argon, phenacyl bromide **2a** (165 mg, 0.83 mmol, 1.3 equiv) was added and dissolved in dry DCM (2.5 mL, 0.26 M). The mixture was stirred for 5 minutes and TMSOF **1b** (100 mg, 0.64 mmol, 1.0 equiv) was added dropwise. The reaction mixture was then cooled to –78 °C using a cryostat (acetone bath), and upon reaching the desired temperature, $BF_3\cdot OEt_2$ (90 µL, 0.70 mmol, 1.1 equiv) was added dropwise. The reaction mixture was then allowed to warm slowly to room temperature and stirred for 16 h. The reaction was quenched with saturated aqueous $NaHCO_3$ and extracted with DCM/H_2O. The crude product was purified by silica gel flash chromatography (70:30 Petroleum Ether:EtOAc) to yield an inseparable 1:0.3 diastereomeric mixture of 5-(2-bromo-1-hydroxy-1-phenylethyl)furan-2(5*H*)-one **7ba** (175 mg, 98% yield) as an amorphous white solid.

Data for (±)-**7ba** (major diasteroisomer):

TLC: R_f= 0.45 (70:30 Petroleum ether/EtOAc)

^{1}H NMR (600 MHz, DMSO-D_6) δ 7.69 (dd, *J* = 5.8, 1.5 Hz, 1H, H4), 7.36 – 7.31 (m, 2H, Ph), 7.29 – 7.23 (m, 2H, Ph), 7.21 (m, 1H, Ph), 6.22 (d, *J* = 1.0 Hz, 1H, OH), 5.99 (dd, *J* = 5.8, 1.8 Hz, 1H, H3), 5.39 (dd, *J* = 1.8, 1.5 Hz, 1H, H5), 4.11 (d, *J* = 11.0 Hz, 1H, H2′a), 3.95 (d, *J* = 11.0 Hz, 1H, H2′b).

^{13}C NMR DEPTq-135 (100 MHz, $CDCl_3$): δ 172.2 (Cq, C2), 154.1 (CH, C4), 136.6 (Cq, Ph), 128.9 CH, Ph), 128.5 (2C, CH, Ph), 125.5 (2C, CH Ph), 122.6 (CH, C3), 84.6 (CH, C5), 75.8 (Cq, C1'), 42.3 (CH_2, C2′).

Data for (±)-**7ba** (minor diastereoisomer):

TLC: R_f= 0.45 (70:30 Petroleum ether/EtOAc)

^{1}H NMR (600 MHz, DMSO-D_6) δ 7.49 – 7.44 (m, 2H), 7.37 – 7.31 (m, 2H), 7.30 – 7.23 (m, 1H), 7.13 (dd, *J* = 5.8, 1.5 Hz, 1H, H4), 6.18 (dd, *J* = 5.8, 2.0 Hz, 1H, H3), 5.99 (s, 1H, OH), 5.53 (m, 1H, H5), 4.15 (d, *J* = 10.8 Hz, 1H, H2′a), 3.87 (d, *J* = 10.9 Hz, 1H, H2′b).

^{13}C NMR DEPTq-135 (100 MHz, $CDCl_3$): δ 172.2 (Cq, C2), 155.9 (CH, C4), 139.3 (Cq, Ph), 128.7 CH, Ph), 128.6 (2C, CH, Ph), 125.3 (2C, CH Ph), 123.1 (CH, C3), 86.1 (CH, C5), 75.5 (Cq, C1'), 40.8 (CH_2, C2′).

4. Photoinduced γ-Alkylation of 2-Silyloxyfurans with 2-Bromoacetophenones

4.1 Optimization Studies

Table S2. Photocatalyst Screening[a]

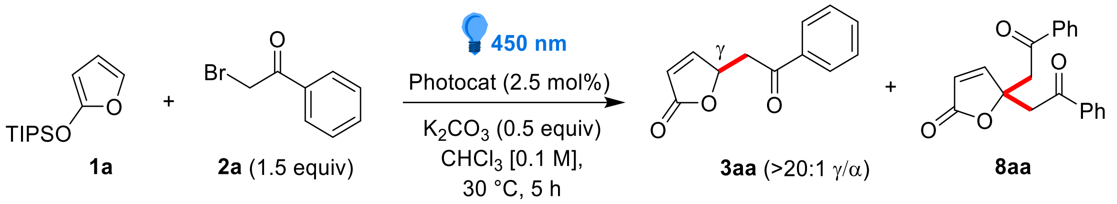

Entry	Photocatalyst (2.5 mol%)	E (V) (M*/M+)[b]	E (V) (M*/M-)[b]	Yield %[c] (3aa)	3aa:8aa[d]
1	*fac*-Ir(ppy)$_3$ (**Ir-1**)	–1.73	+0.31	35	1:0.17
2	(Ir[dF(CF$_3$)ppy]$_2$(dtbpy))PF$_6$ (**Ir-2**)	–0.89	+1.21	9	nd
3	[Ir(dtbbpy)(ppy)$_2$]PF$_6$ (**Ir-3**)	–0.96	+0.66	9	nd
4	Ru(bpy)$_3$Cl$_2$ (**Ru-1**)	–0.81	+0.77	20	1:0.08
5	4CzIPN	–1.12	+1.43	6	nd
6	Rose Bengal	–1.21	+1.77	<3	nd
7	Eosin Y	–1.06	+0.79	<3	nd
8	**Ir-1** (no light)		*No reaction*		
9	No Cat.		*No Reaction*		

[a] Standard reaction conditions: TIPSOF **1a** (0.1 mmol, 1 equiv), phenacyl bromide **2a** (0.15 mmol, 1.5 equiv), photocatalyst (2.5 mol%) in 1 mL of degassed $CHCl_3$, for 5 h at 30 °C.
[b] Unless otherwise noted, values are given in volts (V) versus the saturated calomel electrode (SCE), as reported in Ref. 15.
[c] Isolated yield of **3aa**.
[d] Determined by ^{1}H NMR of the crude. nd = data not determined.

Table S3. Screening of Solvents[a]

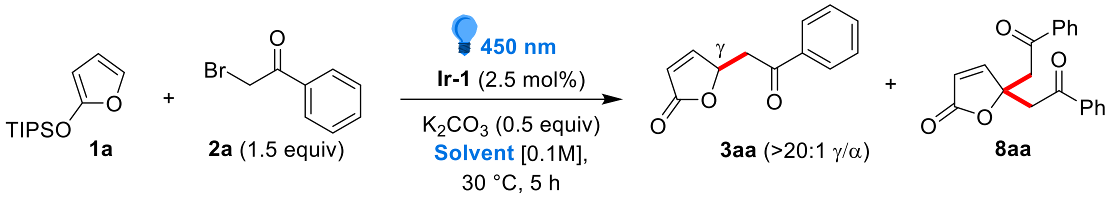

Entry	Solvent	Yield % (3aa)[b]	3aa:8aa[c]
**1**	$CHCl_3$	30	1:0.16
**2**	$CDCl_3$	30	1:0.14
**3**	CH_2Cl_2	20	1:0.05
**4**	1,2-dichloroethane (DCE)	42	1:0.1
**5**	MeCN	*Degradation products*	
**6**	Toluene	8	nd
**7**	Xylenes	8	nd
	Et_2O	*Degradation products*	
	THF	*Degradation products*	
	DMF	*Degradation products*	

[a] Standard reaction conditions: TIPSOF **1a** (0.1 mmol, 1 equiv), phenacyl bromide **2a** (0.15 mmol, 1.5 equiv), **Ir-1** (2.5 mol%) in 1 mL of degassed **solvent**, for 5 h at 30 °C.
[b] Isolated yield of **3aa**.
[c] Determined by ^1H NMR of the crude. nd = data not determined.

Table S4. Additives & Concentration[a]

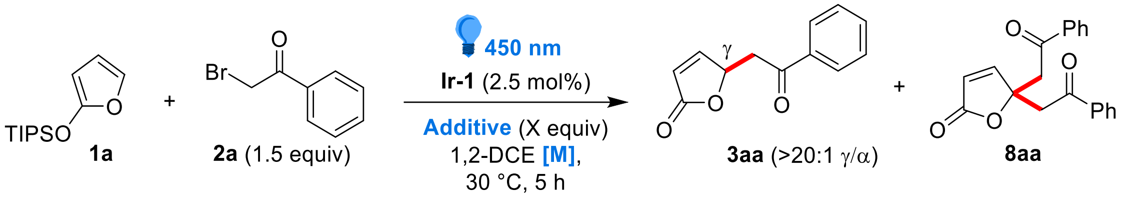

Entry	Additive (equiv)	1a [M]	Yield % (3aa)[b]	3aa:8aa[c]
**1**	K_2CO_3 (0.5 equiv)	0.1	42	1:0.1
**2**	K_2CO_3 (0.5 equiv)	0.2	30	1:0.1
**3**	K_2CO_3 (0.5 equiv)	0.06	45	1:0.1
**5**	K_2CO_3 (2.0 equiv)	0.06	35	1:0.17
**6**	Pr^i_2EtN (0.5 equiv)	0.06	*No Reaction*	
**7**	*none*	0.06	50	1:0.06

[a] Standard reaction conditions: TIPSOF **1a** (0.1 mmol, 1 equiv), phenacyl bromide **2a** (0.15 mmol, 1.5 equiv), **Ir-1** (2.5 mol%) in degassed 1,2-DCE, for 5 h at 30 °C.
[b] Isolated yield of **3aa**.
[c] Determined by ^{1}H NMR of the crude.

Table S5. Stoichiometry & Time[a]

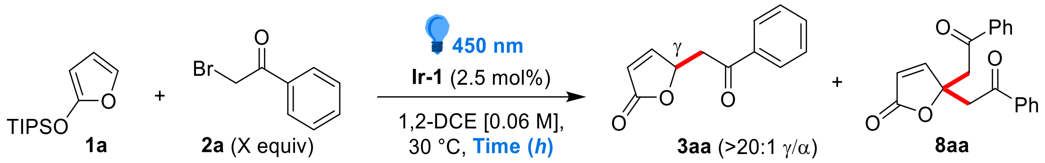

Entry	1a:2a (equiv)	Time (*h*)	Yield %[b] (3aa)	3aa:8aa[c]
1	1:1.5	5	50	1:0.06
2	1:3	5	43	1:0.06
3	1:1	5	40	1:0.08
5	1.5:1	5	90	1:0.03
6	3:1	5	90	1:0.03
7	1.5:1	24	70	1:0.03
8	1.5:1	1	80	1:0.03
9	1.5:1	3	93	1:0.03

[a] Standard reaction conditions: TIPSOF **1a** (X equiv), phenacyl bromide **2a** (Y equiv), **Ir-1** (2.5 mol%) in 1.7 mL of degassed 1,2-DCE, for 5 h at 30 °C.
[b] Isolated yield of **3aa**.
[c] Determined by ^{1}H NMR of the crude.

Table S6. Evaluating the Contribution of the Silyl Group within **1**.[a]

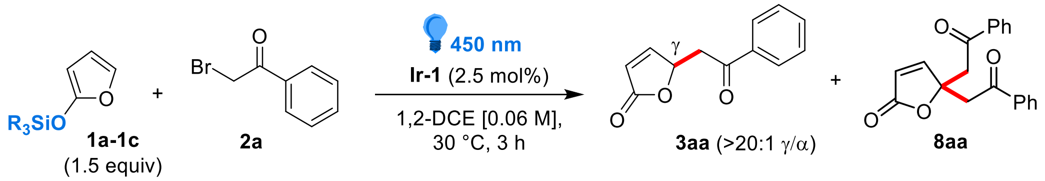

Entry	R_3Si	1	Yield %[b] (3aa)	3aa:8aa[c]
1	TIPS	**1a**	93	1:0.03
2	TMS	**1b**	88	1: 0.01
3	TBS	**1c**	90	1:0.03

[a] Standard reaction conditions: **1a** (0.15 mmol, 1.5 equiv), phenacyl bromide **2a** (0.10 mmol, 1.0 equiv), **Ir-1** (2.5 mol%) in 1.7 mL of degassed 1,2-DCE, for 5 h at 30 °C.
[b] Isolated yield of **3aa**.
[c] Determined by ^{1}H NMR of the crude.

4.2 Reaction monitoring via ^{1}H-NMR analyses of the crudes

During the optimization survey, we were challenged by the formation of γ,γ-bisalkylated product **8aa**. As an example, in Figure S4 we report the ^{1}H NMR (400 MHz, $CDCl_3$) of the crude related to entry 1 of Table S1. We were able to isolate compound **8aa** via flash chromatographic purification of the crude (80/20 Petroleum Ether:EtOAc). For a proposal of the mechanism by which this product could be formed, see Section 7.

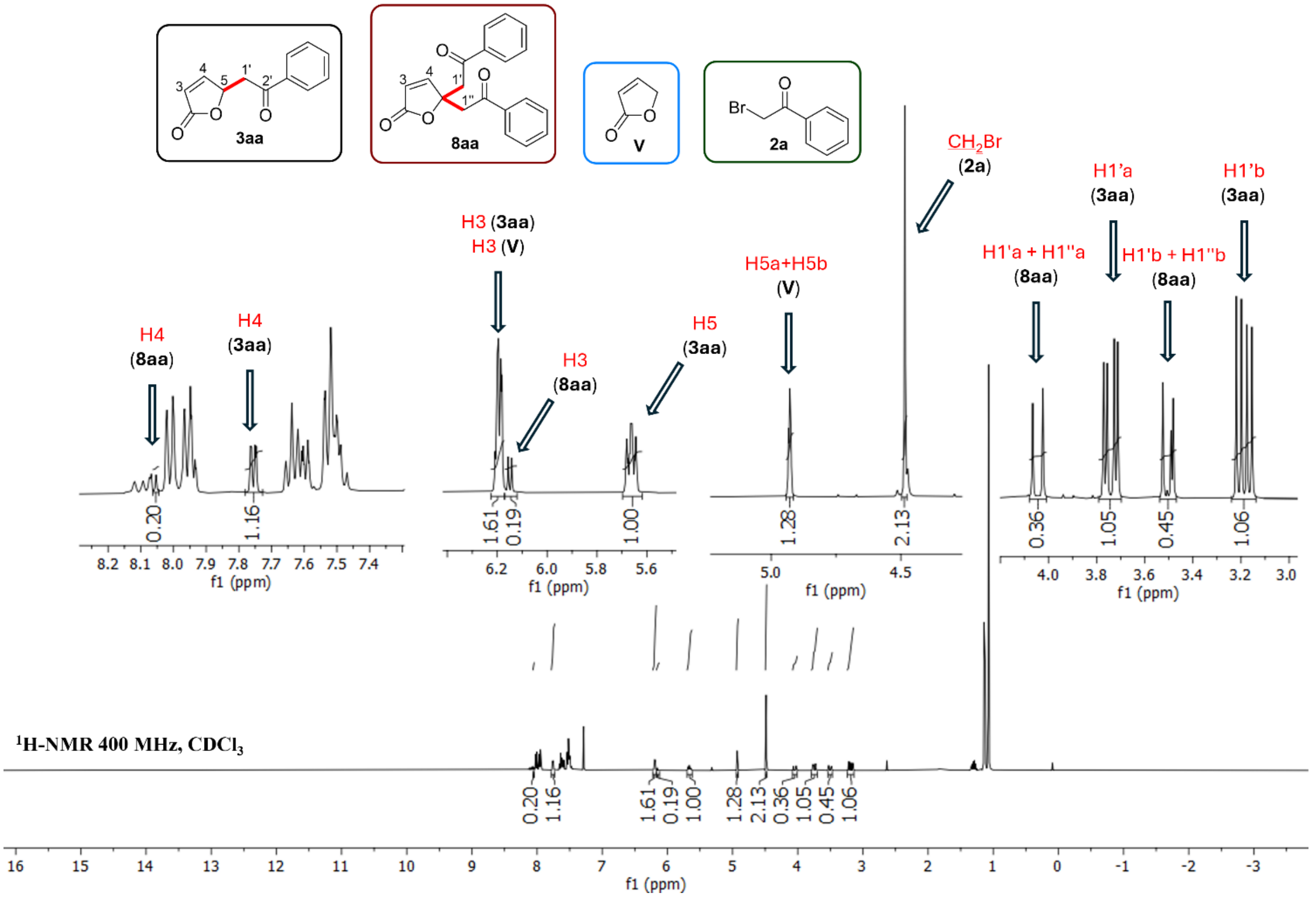

Figure S4. ^{1}H NMR of the crude of the reaction described in entry 1 of Table S2.

Data for 2,2'-(5-oxo-2,5-dihydrofuran-2,2-diyl)bis(1-phenylethan-1-one) (±)-**8aa**:

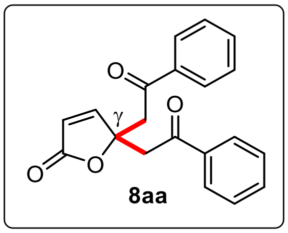

TLC: R_f= 0.47 (80:20 Petroleum ether/EtOAc)

^{1}H NMR (400 MHz, $CDCl_3$): δ 8.07 (d, J = 5.7 Hz, 1H, H4), 7.95 (dt, J = 7.2, 1.3 Hz, 4H, Ph), 7.61 (td, J = 7.2, 1.3 Hz, 2H, Ph), 7.50 (t, J = 7.8 Hz, 5H, Ph), 6.16 (dd, J = 5.7 Hz, 1H, H3), 4.05 (d, J = 17.0 Hz, 2H, H1′a, H1″a), 3.51 (d, J = 17.0, 2H, H1′b, H1″b).

^{13}C NMR (100 MHz, $CDCl_3$): δ 196.2 (2C, Cq, C2′), 171.5 (Cq, C2), 159.5 (CH, C4), 136.6 (2C, Cq, Ph), 133.8 (2C, CH, Ph), 128.8 (4C, CH, Ph), 128.2 (4C, CH, Ph), 121.1 (CH, C3), 86.9 (CH, C5), 44.1 (2C, CH_2, C1′).

4.3 Representative Procedure A. (Table S5, entry 9)

4.3.1 Preparation of (±)-5-(2-oxo-2-phenylethyl)furan-2(5*H*)-one (3aa)

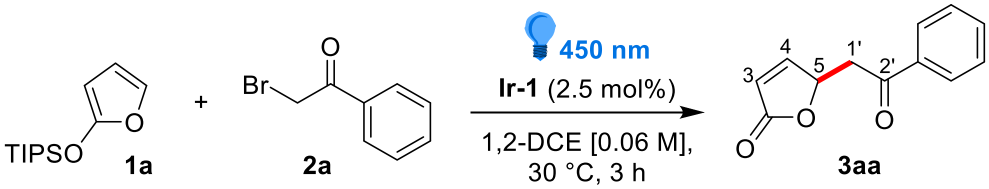

To a 4 mL vial equipped with a magnetic stir bar, 2-triisopropilsilyloxyfuran **1a** (TIPSOF, 36 mg, 0.15 mmol, 1.5 equiv) and phenacyl bromide **2a** (20 mg, 0.1 mmol, 1.0 equiv), were dissolved in 1.0 mL of degassed 1,2-DCE at room temperature under Ar. Afterwards, a solution of photocatalyst *fac*-Ir(ppy)$_3$ (**Ir-1**, 1.3 mg, 0.002 mmol, 0.025 equiv) in 0.7 mL of 1,2-DCE was added by a syringe. The vial was sealed with a screw-top cap with septum and then vacuumed and backfilled with argon for 3 times. The vial was sealed with Parafilm and then stirred under 450 nm irradiation at 30 °C for 3 hours and then concentrated under vacuum. The crude was then purified by silica-gel flash chromatography (80/20 Petroleum Ether:EtOAc) to yield 19 mg of 5-(2-oxo-2-phenylethyl)furan-2(5*H*)-one) (**3aa**, 93% yield) as brown crystals.

<u>Data for (±)-**3aa**</u>:

TLC: R_f= 0.4 (80:20 Petroleum ether/EtOAc)

m.p.: +105.1 °C (see Figure S5)

<u>^{1}H NMR (400 MHz, $CDCl_3$)</u> δ 7.96 (m, 2H, Ph), 7.76 (dd, *J* = 5.7, 1.4 Hz, 1H, H4), 7.64 (m, 1H, Ph), 7.51 (m, 2H, Ph), 6.20 (dd, *J* = 5.7, 1.9 Hz, 1H, H3), 5.67 (ddt, *J* = 7.6, 5.7, 1.7, 1.7 Hz, 1H), 3.75 (dd, *J* = 17.6, 5.7 Hz, 1H, H1′a), 3.19 (dd, *J* = 17.6, 8.5 Hz, 1H, H1′b).

<u>^{13}C NMR (100 MHz, $CDCl_3$)</u>: δ 195.7 (Cq, C2'), 172.5 (Cq, C2), 156.4 (CH, C4), 135.9 (Cq, Ph), 134.0 CH, Ph), 128.9 (2C, CH, Ph), 128.1 (2C, CH Ph), 121.8 (CH, C3), 79.4 (CH, C5), 41.79 (CH_2, C1').

HR-MS (ESI) Calcd.: *m/z* 203.0708 $[C_{12}H_{10}O_3 + H]^+$;
Found: *m/z* 203.0710 $[M + H]^+$
Δppm ±SD: 0.82 ± 0.75

Calcd.: *m/z* 225.0528 $[C_{12}H_{10}O_3 + Na]^+$;
Found: *m/z* 203.0530 $[M + Na]^+$
Δppm ±SD: 1.04 ± 1.03

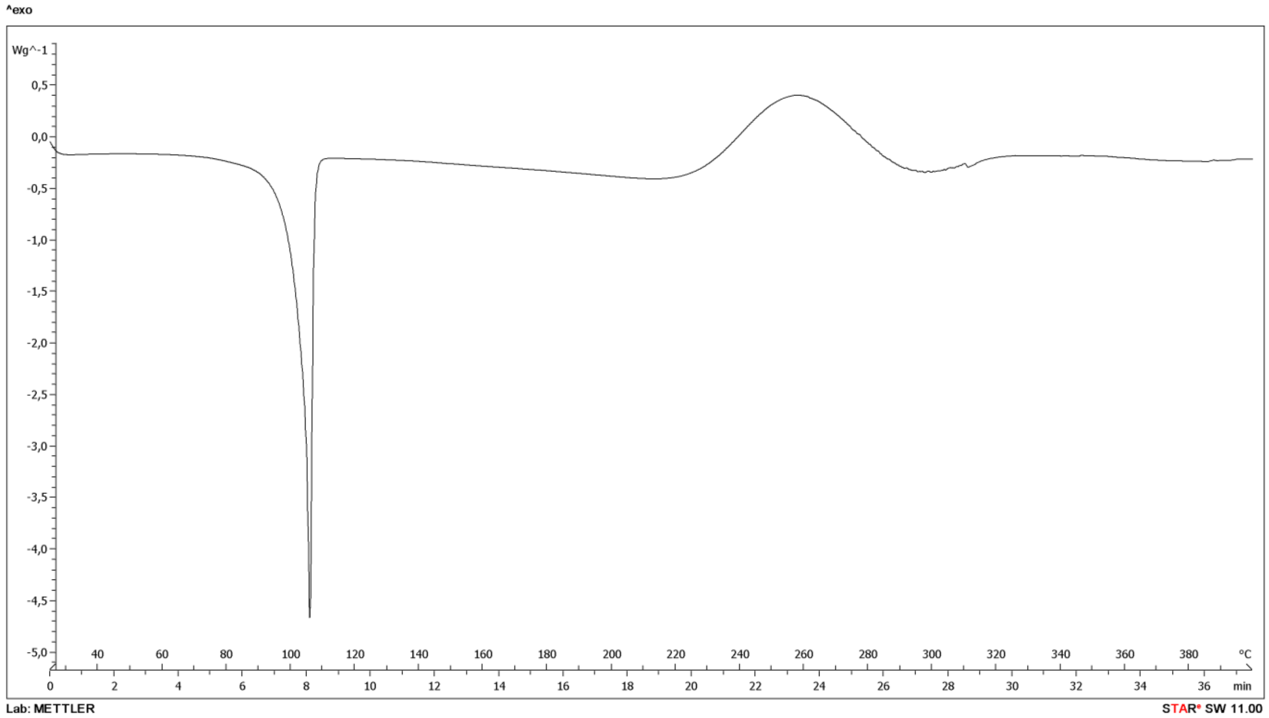

Figure S5. DSC analysis of **3aa** crystals

4.4 Scale-up procedure to access 3aa

Due to the higher volumes needed, the gram scale reaction was performed with illumination at 465 nm, conducted using a 1-meter blue LED strip, 14.4W "LEDXON MODULAR 9009083 LED, SINGLE 5050" (Farnell) that was wrapped around a crystallizing dish. A fan was used to cool down the reactor. The reaction temperature, measured within the reaction vessel using a thermometer, was around 30 ºC (Figure S6).[39]

To an oven-dried 100 mL round-bottom flask equipped with a magnetic stir bar, 2-trimethylsilyloxyfuran **1a** (TMSOF, 1.2 g, 7.5 mmol, 1.5 equiv) and phenacyl bromide **2a** (1.0 g, 5.0 mmol, 1.0 equiv), were dissolved in 60.0 mL of degassed 1,2-DCE at room temperature under Ar. Afterwards, a solution of photocatalyst *fac*-Ir(ppy)$_3$ (**Ir-1**, 65.7 mg, 0.1 mmol, 0.025 equiv) in degassed 1,2-DCE (5.0 mL) was added by a syringe. The round-bottom flask was sealed with septum and then vacuumed and backfilled with argon for 3 times. The round-bottom flask was sealed with Parafilm, stirred under 465 nm irradiation at 30 °C for 3 hours, and then concentrated under vacuum for 12 h. The ^{1}H NMR analysis of the crude (913 mg) revealed the formation of the sole **3aa** in a 90% NMR purity. To remove the remaining traces of the photocatalyst, the crude was purified by silica-gel flash chromatography (80/20 Petroleum Ether:EtOAc) to yield 5-(2-oxo-2-phenylethyl)furan-2(5*H*)-one) (**3aa**, 862 mg, 85% yield) as light brown crystals.

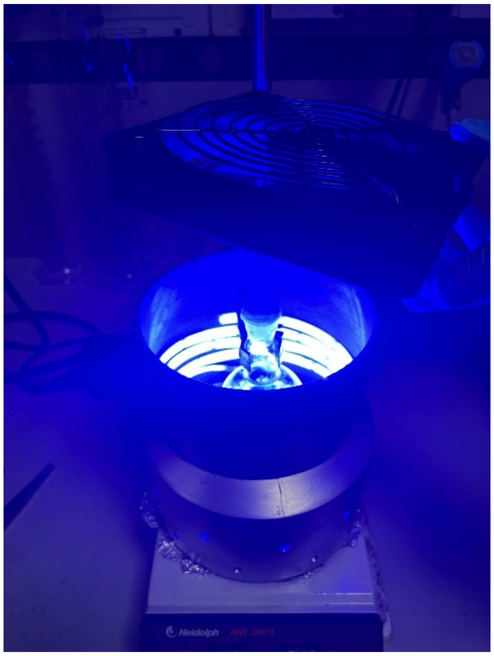

Figure S6. Photoreactor apparatus used for the gram-scale reaction.

4.5 Substrate Scope

Table S7. Scope of the photoinduced alkylation of silyloxyfurans **1a**, **1b**, **1c** and **1d** with substituted α-bromoketones **2a-r** promoted by **Ir-1**.

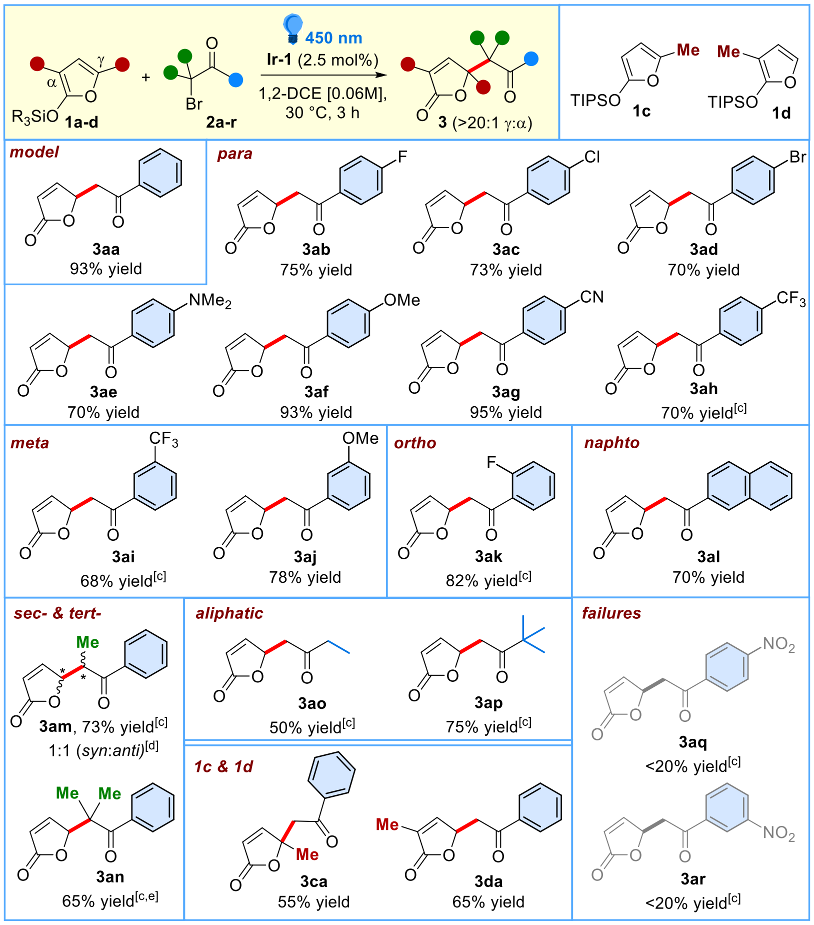

Preparation of (±)-5-(2-(4-fluorophenyl)-2-oxoethyl)furan-2(5*H*)-one (3ab)

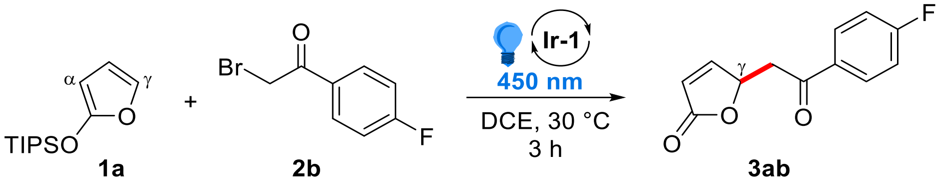

Compound (±)-**3ab** was prepared according to Representative Procedure A using: TIPSOF **1a** (36 mg, 0.15 mmol, 1.5 equiv), 2-bromo-1-(4-fluorophenyl)ethan-1-one (**2b**, 22 mg, 0.1 mmol, 1.0 equiv), **Ir-1** (1.3 mg, 0.002 mmol, 0.025 equiv), in degassed 1,2-DCE (1.7 mL, 0.06 M), at 30 °C for 3 h. The crude was concentrated and then purified by silica-gel flash chromatography (70/30 Petroleum Ether:EtOAc) to yield pure 5-(2-(4-fluorophenyl)-2-oxoethyl)furan-2(5*H*)-one (**3ab**, 16.5 mg, 75% yield) as a yellow, amorphous solid.

Data for (±) **3ab** (major product):

TLC: R_f = 0.38 (70:30 Petroleum ether/EtOAc)

^{1}H NMR (400 MHz, $CDCl_3$): δ 7.99 (m, 2H, Ar), 7.7 (dd, *J* = 5.7, 1.4 Hz, 1H, H4), 7.20 (m, 2H, Ar), 6.20 (dd, *J* = 5.7, 1.9 Hz, 1H, H3), 5.70 (dddd, *J* = 7.8, 5.8, 1.9, 1,4 Hz, 1H, H5), 3.70 (dd, *J* = 17.5, 5.8 Hz, 1H, H1′a), 3.16 (dd, *J* = 17.5, 8.2 Hz, 1H, H1′b).

^{13}C NMR (100 MHz, $CDCl_3$): δ 194.1 (Cq, C2′), 172.4 (Cq, C2), 166 (d, $^1J_{CF}$ = 255 Hz, Cq, Ar) 156.3 (CH, C4), 132.4 (Cq, Ar), 130.8 (d, $^2J_{CF}$ = 10 Hz, 2C, CH, Ar), 121.9 (CH, C3), 116.10 (d, $^3J_{CF}$ = 21,9 Hz, 2C, CH, Ar), 79.3 (CH, C5), 41.7 (CH_2, C1′).

HR-MS (ESI) Calcd.: *m/z* 221.0614 $[C_{12}H_9FO_3 + H]^+$;
Found: *m/z* 203.0614 $[M + H]^+$
Δppm ±SD: 0.45 ± 0.45

Preparation of (±)-5-(2-(4-chlorophenyl)-2-oxoethyl)furan-2(5*H*)-one (3ac)

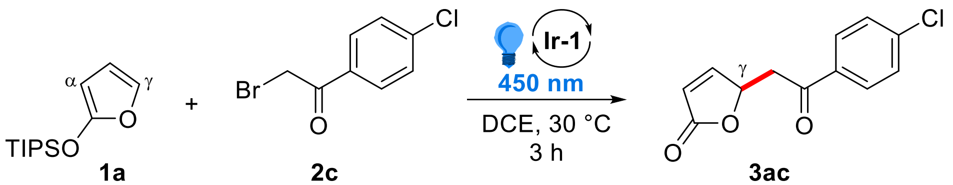

Compound (±)-**3ac** was prepared according to Representative Procedure A using: TIPSOF **1a** (36 mg, 0.15 mmol, 1.5 equiv), 2-bromo-1-(4-chlorophenyl)ethan-1-one (**2c**, 23 mg, 0.1 mmol, 1.0 equiv), **Ir-1** (1.3 mg, 0.002 mmol, 0.025 equiv), in degassed 1,2-DCE (1.7 mL, 0.06 M), at 30 °C for 3 h. The crude was purified by silica-gel flash chromatography (70/30 Petroleum Ether:EtOAc) to yield pure 5-(2-(4-chlorophenyl)-2-oxoethyl)furan-2(5*H*)-one (**3ac**, 17.3 mg, 73% yield) as a yellow amorphous solid.

Data for (±) **3ac**:

TLC: R_f = 0.4 (70:30 Petroleum ether/EtOAc)

^{1}H NMR (400 MHz, $CDCl_3$): δ 7.90 (m, 2H, Ar), 7.74 (dd, *J* = 5.7, 1.4 Hz, 1H, H4), 7.49 (m, 2H, Ar), 6.20 (dd, *J* = 5.7, 1.9 Hz, 1H, H3), 5.65 (dddd, *J* = 7.7, 5.8, 1.9, 1.4 Hz, 1H, H5), 3.69 (dd, *J* = 17.6, 5.9 Hz, 1H, H1′a), 3.16 (dd, *J* = 17.6, 8.2 Hz, 1H, H1′b).

^{13}C NMR (100 MHz, $CDCl_3$): δ 194.5 (Cq, C2′), 172.4 (Cq, C2), 156.2 (CH, C4), 140.6 (Cq, Ar), 134.2 (Cq, Ar), 129.5 (2C, CH, Ar), 129.3 (2C, CH, Ar), 121.9 (CH, C3), 79.2 (CH, C5), 41.75 (CH_2, C1′).

HR-MS (ESI) Calcd.: *m/z* 237.0318 $[C_{12}H_9ClO_3 + H]^+$;
Found: *m/z* 237.0323 $[M + H]^+$
Δppm ±SD: 2.11 ± 0.00

Calcd.: *m/z* 259.0138 $[C_{12}H_9ClO_3 + Na]^+$;
Found: *m/z* 259.0142 $[M + Na]^+$
Δppm ±SD: 1.42 ± 0.59

Preparation of (±)-5-(2-(4-bromophenyl)-2-oxoethyl)furan-2(5*H*)-one (3ad)

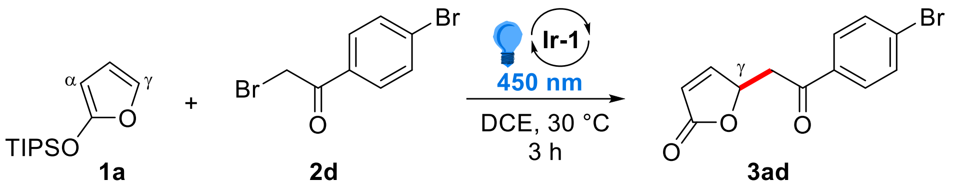

Compound (±)-**3ad** was prepared according to Representative Procedure A using: TIPSOF **1a** (36 mg, 0.15 mmol, 1.5 equiv), 2-bromo-1-(4-bromophenyl)ethan-1-one **6c** (28 mg, 0.1 mmol, 1.0 equiv), **Ir-1** (1.3 mg, 0.002 mmol, 0.025 equiv), in degassed 1,2-DCE (1.7 mL, 0.06 M), at 30 °C for 3 h. The crude was purified by silica-gel flash chromatography (70/30 Petroleum Ether:EtOAc) to yield pure 5-(2-(4-bromophenyl)-2-oxoethyl)furan-2(5*H*)-one (**3ad**, 20 mg, 70% yield) as a yellow amorphous solid.

Data for (±) **3ad**:

TLC: R_f = 0.4 (70:30 Petroleum ether/EtOAc)

^{1}H NMR (400 MHz, $CDCl_3$): δ 7.82 (m, 2H, Ar), 7.73 (dd, *J* = 5.7, 1.4 Hz, 1H, H4), 7.66 (m, 2H, Ar), 6.20 (dd, *J* = 5.7, 1.9 Hz, 1H, H3), 5.65 (dddd, *J* = 7.7, 5.8, 1.9, 1.4 Hz, 1H, H5), 3.69 (dd, *J* = 17.6, 5.9 Hz, 1H, H1′a), 3.15 (dd, *J* = 17.6, 8.2 Hz 1H, H1′b).

^{13}C NMR (100 MHz, $CDCl_3$): δ 194.7 (Cq, C2′), 172.4 (Cq, C2), 156.2 (CH, C4), 134.60 (Cq, Ar), 132.3 (2C, CH, Ar), 129.5 (2C, 2H, Ar), 129.3 (Cq, Ar), 121.9 (CH, C3), 79.2 (CH, C5), 41.73 (CH_2, C1′).

HR-MS (ESI) Calcd.: *m/z* 280.9813 [$C_{12}H_9BrO_3$+ H]$^+$;
Found: *m/z* 280.9817 [M + H]$^+$
Δppm ±SD: 1.30 ± 0.21

Calcd.: *m/z* 302.9633 [$C_{12}H_9BrO_3$+ Na]$^+$;
Found: *m/z* 302.9635 [M + Na]$^+$
Δppm ±SD: 0.88 ± 0.95

Preparation of (±)-5-(2-(4-(dimethylamino)phenyl)-2-oxoethyl)furan-2(5*H*)-one (3ae)

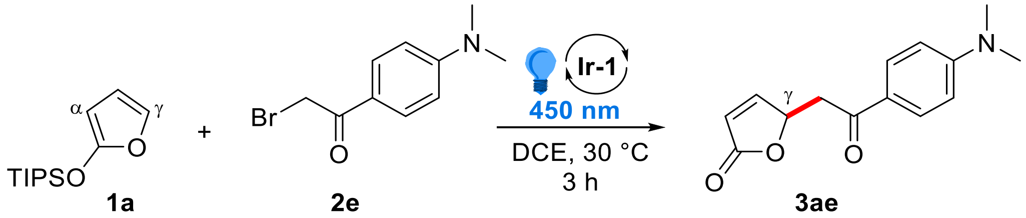

Compound (±)-**3ae** was prepared according to Representative Procedure A using: TIPSOF **1a** (36 mg, 0.15 mmol, 1.5 equiv), 2-bromo-1-(4-(dimethylamino)phenyl)ethan-1-one (**2e**, 24 mg, 0.1 mmol, 1.0 equiv), **Ir-1** (1.3 mg, 0.002 mmol, 0.025 equiv), in degassed 1,2-DCE (1.7 mL, 0.06 M), at 30 °C for 3 h. The crude was purified by silica-gel flash chromatography (70/30 Petroleum Ether:EtOAc) to yield pure 5-(2-(4-(dimethylamino)phenyl)-2-oxoethyl)furan-2(5*H*)-one (**3ae**, 17 mg, 70% yield) as a brownish-orange amorphous solid.

Data for (±) **3ae**:

TLC: R_f = 0.3 (60:40 Petroleum ether/EtOAc)

^{1}H NMR (400 MHz, $CDCl_3$): δ 7.91–7.80 (m, 2H, Ar), 7.78 (dd, *J* = 5.7, 1.5 Hz, 1H, H4), 6.72–6.62 (m, 2H, Ar), 6.15 (dd, *J* = 5.7, 2.0 Hz, 1H, H3), 5.64 (dddd, *J* = 9.0, 5.6, 2.0, 1.5 Hz, 1H, H5), 3.68 (dd, *J* = 16.9, 5.5 Hz, 1H, H1′a), 3.10 (s, 6H, NMe), 3.05 (dd, *J* = 16.9, 8.9 Hz, 1H, H1′b).

^{13}C DEPTq-135 NMR (100 MHz, $CDCl_3$): δ 200.0 (Cq, C2′), 157.2 (CH, C4), 153.0 (Cq, Ar), 130.4 (2C, 2H, Ar), 121.3 (CH, C3), 110.7 (2C, CH, Ar), 80.2 (CH, C5), 41.0 (CH_2, C1′), 40.0 (2C, CH_3, NMe_2).

HR-MS (ESI)
Calcd.: *m/z* 246.1130 $[C_{14}H_{15}NO_3 + H]^+$;
Found: *m/z* 246.1138 $[M + H]^+$
Δppm ±SD: 3.25 ± 1.46

Calcd.: *m/z* 268.0950 $[C_{14}H_{15}NO_3 + Na]^+$;
Found: *m/z* 268.0956 $[M + Na]^+$
Δppm ±SD: 2.11 ± 0.78

Preparation of (±)5-(2-(4-methoxyphenyl)-2-oxoethyl)furan-2(5*H*)-one (3af)

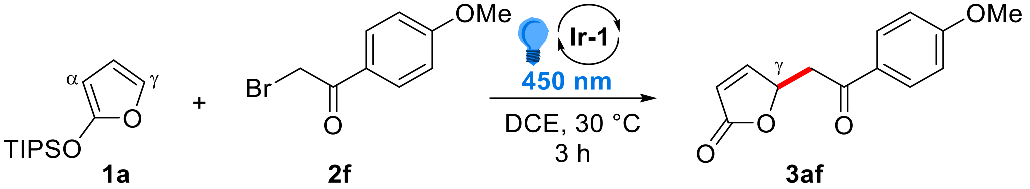

Compound (±)-**3af** was prepared according to Representative Procedure A using: TIPSOF **1a** (36 mg, 0.15 mmol, 1.5 equiv), 2-bromo-1-(4-methoxyphenyl)ethan-1-one (**2f**, 23 mg, 0.1 mmol, 1.0 equiv), **Ir-1** (1.3 mg, 0.002 mmol, 0.025 equiv), in degassed 1,2-DCE (1.7 mL, 0.06 M), at 30 °C for 3 h. The crude was purified by silica-gel flash chromatography (70/30 Petroleum Ether:EtOAc) to yield pure 5-(2-(4-methoxyphenyl)-2-oxoethyl)furan-2(5*H*)-one (**3af**, 21.6 mg, 93% isolated yield) as a yellow amorphous solid.

Data for (±) **3af:**

TLC: R_f = 0.2 (70:30 Petroleum ether/EtOAc)

^{1}H NMR (400 MHz, $CDCl_3$): δ 7.93 (m, 2H, Ar), 7.75 (dd, *J* = 5.7 Hz, 1.5 Hz, 1H, H4), 6.97 (m, 2H, Ar), 6.17 (dd, *J* = 5.7, 1.9 Hz, 1H, H3), 5.64 (dddd, *J* = 8.6, 5.7, 1.9, 1,5 Hz, 1H, H5), 3.90 (s, 3H, OMe), 3.69 (dd, *J* = 17.3, 5.7 Hz, 1H, H1′a), 3.12 (dd, *J* = 17.3, 8.5 Hz, 1H, H1′b).

^{13}C NMR (100 MHz, $CDCl_3$): δ 194.2 (Cq, C2′), 172.6 (Cq, C2), 164.2 (Cq, Ar), 156.7 (CH, C4), 130.5 (2C, CH, Ar), 129.0 (Cq, Ar), 121.6 (CH, C3), 114,0 (2C, CH, Ar), 79.7 (CH, C5), 55.6 (CH_3, OMe), 41.4 (CH_2, C1′).

HR-MS (ESI) Calcd.: *m/z* 233.0814 $[C_{13}H_{12}O_4 + H]^+$;
Found: *m/z* 233.0817 $[M + H]^+$
Δppm ±SD: 1.14 ± 0.25

Calcd.: *m/z* 255.0634 $[C_{13}H_{12}O_4 + Na]^+$;
Found: *m/z* 255.0640 $[M + Na]^+$
Δppm ±SD: 2.35 ± 0.68

Preparation of (±)-5-(2-(4-cyanophenyl)-2-oxoethyl)furan-2(5*H*)-one (3ag)

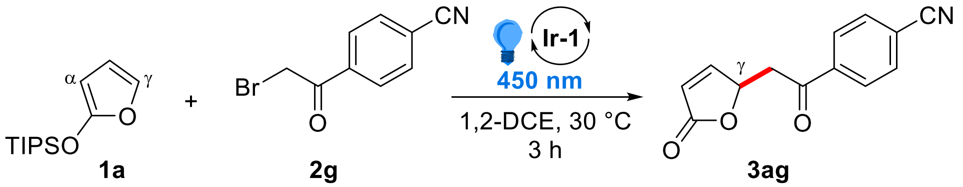

Compound (±)-**3ag** was prepared according to Representative Procedure A using: TIPSOF **1a** (36 mg, 0.15 mmol, 1.5 equiv), 4-(2-bromoacetyl)benzonitrile **2g** (22 mg, 0.1 mmol, 1.0 equiv), **Ir-1** (1.3 mg, 0.002 mmol, 0.025 equiv), in degassed 1,2-DCE (1.7 mL, 0.06 M), at 30 °C for 3 h. The crude was purified by silica-gel flash chromatography (70/30 Petroleum Ether:EtOAc) to yield pure 4-(2-(5-oxo-2,5-dihydrofuran-2-yl)acetyl)benzonitrile (**3ag**, 21.6 mg, 95% yield) as a yellow amorphous solid.

Data for (±) **3ag**:

TLC: R_f = 0.3 (60:40 Petroleum ether/EtOAc)

^{1}H NMR (400 MHz, $CDCl_3$): δ 8.11-7.98 (m, 2H, Ar), 7.90-7.79 (m, 2H, Ar), 7.73 (dd, *J* = 5.7, 1.6 Hz, 1H, H4), 6.22 (dd, *J* = 5.8, 2.0 Hz, 1H, H3), 5.66 (dddd, *J* = 7.9, 6.1, 2.0, 1.6 Hz, 1H, H5), 3.70 (dd, *J* = 17.7, 6.1 Hz, H1′a), 3.22 (dd, *J* = 17.7, 7.7 Hz, H1′b).

^{13}C DEPTq-135 NMR (100 MHz, $CDCl_3$): δ 191.4 (Cq, C2′), 174.1 (Cq, C2), 155.8 (CH, C4), 136.3 (Cq, Ar), 132.8 (2C, CH, Ar), 128.7 (2C, 2H, Ar), 122.2 (CH, C3), 117.6 (Cq, CN), 116.4 (Cq, Ar) 78.8 (CH, C5), 42.0 (CH_2, C1′).

HR-MS (ESI) Calcd.: *m/z* 228.0661 [$C_{13}H_9NO_3$+ H]$^+$;
Found: *m/z* 228.0663 [M + H]$^+$
Δppm ±SD: 0.73 ± 1.27

Preparation of (±)-5-(2-oxo-(4-(trifluoromethyl)phenyl)ethyl) furan-2(5*H*)-one (3ah)

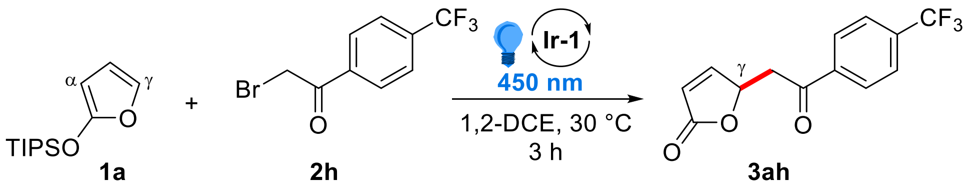

Compound (±)-**3ah** was prepared according to Representative Procedure A using: TIPSOF **1a** (36 mg, 0.15 mmol, 1.5 equiv), 5-(2-oxo-2-(4-(trifluoromethyl)phenyl)ethyl)furan-2(5*H*)-one **2h** (27 mg, 0.1 mmol, 1.0 equiv), **Ir-1** (1.3 mg, 0.002 mmol, 0.025 equiv), in degassed 1,2-DCE (1.7 mL, 0.06 M), at 30 °C for 3 h. The crude was concentrated and then purified by silica-gel flash chromatography (70/30 Petroleum Ether:EtOAc) to yield pure 5-(2-oxo-2-(4-(trifluoromethyl)phenyl)ethyl)furan-2(5*H*)-one (**3ah**, 19.0 mg, 70% yield) as a yellow, amorphous solid.

Data for (±) **3ah**:

TLC: R_f = 0.30 (70:30 Petroleum ether/EtOAc)

^{1}H NMR (400 MHz, $CDCl_3$) δ 8.07 (d, J = 8.2 Hz, 2H, Ar), 7.78 (d, J = 8.1 Hz, 2H, Ar), 7.74 (dd, J = 5.8, 1.5 Hz, 1H, H4), 6.20 (ddd, J = 5.8, 1.7, 1.5 Hz, 1H, H3), 5.66 (dddd, J = 8.0, 6.2, 1.9, 1.7 Hz, 1H, H5), 3.72 (ddd, J = 17.7, 6.1, 1.5 Hz, 1H, H1′a), 3.24 (dd, J = 17.7, 7.8 Hz, 1H, H1′b).

^{13}C NMR (100 MHz, $CDCl_3$) δ 194.8 (Cq, C2′), 172.3 (Cq, C2), 156.0 (CH, C4), 138.5 (Cq, Ar), 135.2 (q, $^2J_{CF}$ = 33.3 Hz, Cq, Ar),), 128.5 (2C, CH, Ar), 126.0 (q, ^3J = 3.7 Hz, 2C, CH, Ar), 123.0 (q, $^1J_{CF}$ = 294.5 Hz, CF_3), 122.0 (CH, C3), 79.0 (CH, C5), 42.0 (CH_2, C1′).

^{19}F NMR (564 MHz, $CDCl_3$):δ –63.2 (bs, 3F; CF_3).

MS (ESI, 100eV): Calculated.: [$M+Na^+$]: 293.0
Found: [$M+Na^+$]: 293.1

Preparation of (±)-5-(2-oxo-(3-(trifluoromethyl)phenyl)ethyl) furan-2(5*H*)-one (3ai)

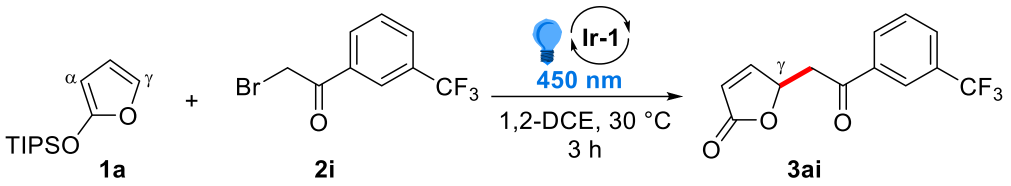

Compound (±)-**3ai** was prepared according to Representative Procedure A using: TIPSOF **1a** (36 mg, 0.15 mmol, 1.5 equiv), 2-bromo-1-(3-(trifluoromethyl)phenyl)ethan-1-one (**2i**, 27 mg, 0.1 mmol, 1.0 equiv), **Ir-1** (1.3 mg, 0.002 mmol, 0.025 equiv), in degassed 1,2-DCE (1.7 mL, 0.06 M), at 30 °C for 3 h. The crude was concentrated and then purified by silica-gel flash chromatography (70/30 Petroleum Ether:EtOAc) to yield pure 5-(2-oxo-2-(3-(trifluoromethyl)phenyl)ethyl)furan-2(5*H*)-one (**3ai**, 18.4 mg, 68% yield) as a yellow, amorphous solid.

Data for (±) **3ai**:

TLC: R_f = 0.32 (70:30 Petroleum ether/EtOAc)

^{1}H NMR (400 MHz, $CDCl_3$) δ 8.21 (s, 1H, Ar), 8.14 (d, *J* = 8.1 Hz, 1H; Ar), 7.89 (d, *J* = 7.8 Hz, 1H, Ar), 7.74 (dd, *J* = 5.7, 1.6 Hz, 1H, H4), 7.67 (t, *J* = 7.8 Hz, 1H, Ar), 6.21 (dd, *J* = 5.8, 1.6 Hz, 1H, H3), 5.67 (dddd, *J* = 8.0, 6.2, 1.9, 1.6 Hz, 1H, H5), 3.73 (dd, *J* = 17.7, 6.1 Hz, 1H, H1′a), 3.24 (dd, *J* = 17.7, 7.8 Hz, 1H, H1′b).

^{13}C NMR (100 MHz, $CDCl_3$) δ 194.4 (Cq, C2′), 172.3 (Cq, C2), 156.0 (CH, C4), 136.4 (Cq, Ar), 131.76 (q, $^2J_{CF}$ = 33.1 Hz, Cq, Ar), 131.28 (d, ^4J = 1.4 Hz, CH, Ar), 130.35 (q, ^3J = 3.6 Hz, CH, Ar), 129.67 (CH, Ar), 124.93 (q, ^3J = 3.7 Hz, CH, Ar), 123,46 (q, $^1J_{CF}$ = 273.7 Hz, CF_3), 122.03 (CH, C3), 78.9 (CH, C5), 41.9 (CH_2, C1′).

^{19}F NMR (564 MHz, $CDCl_3$): δ –62.8 (bs, 3F; CF_3).

MS (ESI, 100eV):	Calculated.:	[M+Na$^+$]: 293.0
	Found:	[M+Na$^+$]: 293.0

Preparation of (±)-5-(2-(3-methoxyphenyl)-2-oxoethyl)furan-2(5*H*)-one (3aj)

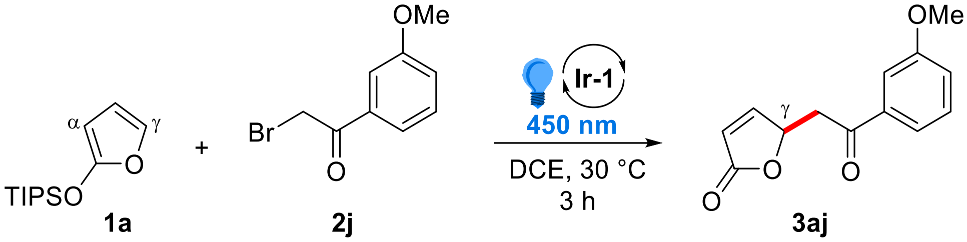

Compound (±)-**3aj** was prepared according to Representative Procedure A using: TIPSOF **1a** (36 mg, 0.15 mmol, 1.5 equiv), 2-bromo-1-(3-methoxyphenyl)ethan-1-one (**2j**, 23 mg, 0.1 mmol, 1.0 equiv), **Ir-1** (1.3 mg, 0.002 mmol, 0.025 equiv), in degassed 1,2-DCE (1.7 mL, 0.06 M), at 30 °C for 3 h. The crude was purified by silica-gel flash chromatography (70/30 Petroleum Ether:EtOAc) to yield pure (±)-5-(2-(3-methoxyphenyl)-2-oxoethyl)furan-2(5*H*)-one (**3aj**, 18 mg, 78% isolated yield) as a yellow amorphous solid.

Data for (±) **3aj:**

TLC: R_f = 0.2 (70:30 Petroleum ether/EtOAc)

^{1}H NMR (400 MHz, $CDCl_3$): δ 7.85 (dd, *J* = 7.8, 1.9 Hz, 1H, Ar), 7.75 (dd, *J* = 5.7, 1.5 Hz, 1H, Ar), 7.56-7.52 (m, 1H, Ar), 7.12-6.95 (m, 2H, Ar + H4), 6.16 (dd, *J* = 5.7, 2.0 Hz, 1H, H3) 5.63 (ddt, *J* = 8.9, 5.7, 1.8 Hz, 1H, H5), 3.94 (s, 3H, OMe), 3.78 (dd, *J* = 18.1, 5.7 Hz, 1H, H1′a), 3.22 (dd, *J* = 18.1, 8.7 Hz, 1H, H1′b).

^{13}C NMR (101 MHz, $CDCl_3$): δ 196.9 (Cq, C2′), 173.0 (Cq, C2), 159.3 (Cq, Ar), 157.0 (CH, C4), 134.8 (CH, Ar), 130.7 (CH, Ar), 126.4 (Cq, Ar), 121.4 (CH, C3), 120. 9 (CH, Ar), 111.7 (CH, Ar), 79.9 (CH, C5), 55.6 (CH_3, OMe), 47.2 (CH_2, C1′).

HR-MS (ESI) Calcd.: *m/z* 233.0814 [$C_{13}H_{12}O_4$+ H]$^+$;
Found: *m/z* 233.0818 [M + H]$^+$
Δppm ±SD: 1.86 ± 0.25

Calcd.: *m/z* 255.0634 [$C_{13}H_{12}O_4$+ Na]$^+$;
Found: *m/z* 255.0640 [M + Na]$^+$
Δppm ±SD: 2.22 ± 0.45

Preparation of (±)-5-(2-(2-fluorophenyl)-2-oxoethyl)furan-2(5*H*)-one (3ak)

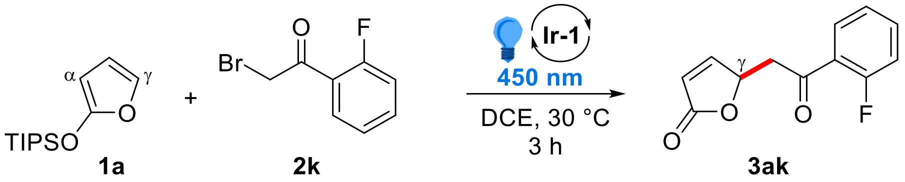

Compound (±)-**3ak** was prepared according to Representative Procedure A using: TIPSOF **1a** (36 mg, 0.15 mmol, 1.5 equiv), 2-bromo-1-(2-fluorophenyl)ethan-1-one (**2k**, 22 mg, 0.1 mmol, 1.0 equiv), **Ir-1** (1.3 mg, 0.002 mmol, 0.025 equiv), in degassed 1,2-DCE (1.7 mL, 0.06 M), at 30 °C for 3 h. The crude was purified by silica-gel flash chromatography (70/30 Petroleum Ether:EtOAc) to yield pure 5-(2-(2-fluorophenyl)-2-oxoethyl)furan-2(5*H*)-one (**3ak**, 18 mg, 82% yield) as a yellow amorphous solid.

Data for (±) **3ak**:

TLC: R_f = 0.38 (70:30 Petroleum ether/EtOAc)

^{1}H NMR (400 MHz, $CDCl_3$): δ 7,95 (td, *J* = 7.6, 1.9 Hz, 1H, Ar), 7.73 (dd, *J* = 5.7, 1.5 Hz, 1H, H4), 7.60 (dddd, *J* = 8.2, 7.1, 5.1, 1.9 Hz, 1H, Ar), 7.33 – 7.25 (m, 1H, Ar), 7.19 (ddd, *J* = 11.4, 8.4, 1.1 Hz, 1H, Ar), 6.19 (dd, *J* = 5.7, 2.0 Hz, 1H, H3), 5.67 (dddd, *J* = 7.9, 6.6, 2.0, 1.4 Hz, 1H, H5), 3.68 (ddd, *J* = 18.4, 6.2, 3.1 Hz, 1H, H1′a), 3.24 (ddd, *J* = 18.4, 7.8, 3.2 Hz, 1H, H1′b).

^{13}C NMR (100 MHz, $CDCl_3$): δ 193.7 (Cq, C2'), 172.6 (Cq, C2), 162.3 (d, $^1J_{CF}$ = 254 Hz, Cq, Ar), 156.3 (CH, C4), 135.6 (d, $^3J_{CF}$ = 9.1 Hz, CH, Ar), 130.6 (d, $^4J_{CF}$ = 1.7 Hz, CH, Ar), 124.8 (d, $^3J_{CF}$ = 3.4 Hz, CH, Ar), 124.4 (d, $^2J_{CF}$ = 13.0 Hz, Cq, Ar), 121,8 (CH, C3), 116.9 (d, $^2J_{CF}$ = 23.5 Hz, CH, Ar), 79.1 (CH, C5), 46.7 (CH_2, C1').

HR-MS (ESI) Calcd.: *m/z* 221.0614 [$C_{12}H_9FO_3$+ H]$^+$;
Found: *m/z* 221.0615 [M + H]$^+$
Δppm ±SD: 0.30 ± 0.52

Calcd.: *m/z* 243.0434 [$C_{12}H_9FO_3$+ Na]$^+$;
Found: *m/z* 243.0435 [M + Na]$^+$
Δppm ±SD: 0.55 ± 0.63

Preparation of (±)-5-(2-(naphthalen-2-yl)-2-oxoethyl)furan-2(5*H*)-one (3al)

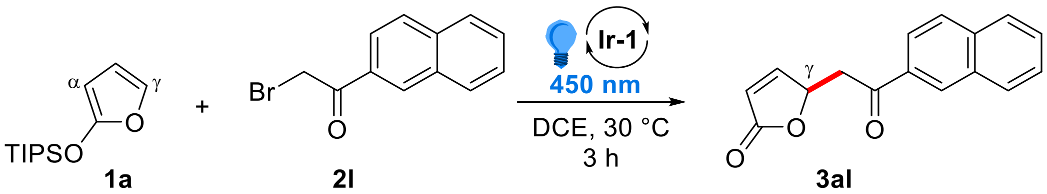

Compound (±)-**3al** was prepared according to Representative Procedure A using: TIPSOF **1a** (36 mg, 0.15 mmol, 1.5 equiv), 2-bromo-1-(naphthalen-2-yl)ethan-1-one (**2l**, 25 mg, 0.1 mmol, 1.0 equiv), **Ir-1** (1.3 mg, 0.002 mmol, 0.025 equiv), in degassed 1,2-DCE (1.7 mL, 0.06 M), at 30 °C for 3 h. The crude was purified by silica-gel flash chromatography (70/30 Petroleum Ether:EtOAc) to yield pure (±)-5-(2-(naphthalen-2-yl)-2-oxoethyl)furan-2(5*H*)-one (**3al**, 18 mg, 70% isolated yield) as an off-white amorphous solid.

Data for (±) **3al**:

TLC: R_f = 0.65 (80:20 Petroleum ether/EtOAc)

^{1}H NMR (400 MHz, $CDCl_3$): δ 8.47 (s, 1H, Ar), 8.06–7.89 (m, 4H, Ar), 7.80 (dd, *J* = 5.7, 1.6 Hz, 1H, H4), 7.64 (m, 2H, Ar), 6.22 (dd, *J* = 5.7, 2.0 Hz, 1H, H3), 5.73 (dddd, *J* = 8.4, 5.7, 2.0, 1.6 Hz, 1H, H5), 3.89 (dd, *J* = 17.5, 5.7 Hz, 1H, H1′a), 3.33 (dd, *J* = 17.5, 8.5 Hz, 1H, H1′b).

^{13}C DEPTq-135 NMR (100 MHz, $CDCl_3$): δ 195.7 (Cq, C2′), 175.7 (Cq, C2), 156.5 (CH, C4), 135.9 (Cq, Ar), 133.3 (Cq, Ar), 132.4 (Cq, Ar), 130.2 (CH, Ar), 129.6 (CH, Ar), 129.0 (CH, Ar), 128.9 (CH, Ar), 127.9 (CH, Ar), 127.2 (CH, Ar), 123.3 (CH, Ar),121.8 (CH, C3), 79.5 (CH, C5), 41.9 (CH_2, C1′).

HR-MS (ESI)
Calcd.: *m/z* 253.0865 $[C_{16}H_{12}O_3 + H]^+$;
Found: *m/z* 253.0867 $[M + H]^+$
Δppm ±SD: 0.92 ± 0.91

Calcd.: *m/z* 275.0684 $[C_{16}H_{12}O_3 + Na]^+$;
Found: *m/z* 275.0689 $[M + Na]^+$
Δppm ±SD: 1.82 ± 0.73

Preparation of a 1:1 *syn*/*anti* mixture of 5-(1-oxo-1-phenylpropan-2-yl)furan-2(5*H*)-one (3am)

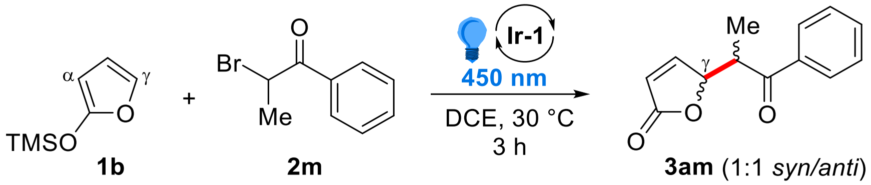

Compound (±)-**3am** was prepared according to Representative Procedure A using: TMSOF **1b** (24 mg, 0.15 mmol, 1.5 equiv), 2-bromo-1-phenylpropan-1-one (**2m**, 21.5 mg, 0.1 mmol, 1.0 equiv), **Ir-1** (1.3 mg, 0.002 mmol, 0.025 equiv), in degassed 1,2-DCE (1.7 mL, 0.06 M), at 30 °C for 3 h. The crude was purified by silica-gel flash chromatography (70/30 Petroleum Ether:EtOAc) to yield a first isomer of 5-(1-oxo-1-phenylpropan-2-yl)furan-2(5*H*)-one (**3am**, 8 mg), and a second congener (**3am**, 8 mg) in a 73% combined, isolated yield, as a yellow amorphous solid.

Data for (±)-**3am** (first isomer):

TLC: R_f = 0.52 (70:30 Petroleum ether/EtOAc)

^{1}H NMR (600 MHz, $CDCl_3$): δ 7.92–7.88 (m, 2H, Ph), 7.63 – 7.58 (m, 1H, Ph), 7.51 (dd, *J* = 5.7, 1.5 Hz, 1H, H4), 7.50 – 7.47 (m, 2H, Ph), 6.11 (dd, *J* = 5.7, 2.0 Hz, 1H, H3), 5.32 (ddd, *J* = 8.8, 2.0, 1.5 Hz, 1H, H5), 3.53 (dq, *J* = 8.8, 7.1 Hz, 1H, H1′), 1.50 (d, *J* = 7.1 Hz, 3H, Me).

^{13}C DEPTq-135 NMR (101 MHz, $CDCl_3$): δ 200.6 (Cq, C2′), 172.6 (Cq, C2), 155.3 (CH, C4), 135.1 (Cq, Ph), 134.0 (CH, Ph), 129.0 (2C, CH, Ph), 128.5 (2C, CH, Ph), 122.0 (CH, C3), 84.7 (CH, C5), 45.5 (CH, C1′), 16.3 (CH_3, Me).

HR-MS (ESI)
Calcd.: *m/z* 217.0865 [$C_{13}H_{12}O_3$+ H]$^+$;
Found: *m/z* 217.0860 [M + H]$^+$
Δppm ±SD: 3.07 ± 2.08

Calcd.: *m/z* 239.0684 [$C_{13}H_{12}O_3$+ Na]$^+$;
Found: *m/z* 239.0685 [M + Na]$^+$
Δppm ±SD: 0.84 ± 0.42

Data for (±)-**3am** (second isomer):

TLC: R_f = 0.46 (70:30 Petroleum ether/EtOAc)

^{1}H NMR (400 MHz, $CDCl_3$): δ 8.01–7.96 (m, 2H, Ph), 7.78 (dd, *J* = 5.8, 1.6 Hz, 1H, H4), 7.68–7.61 (m, 1H, Ph), 7.53 (dd, *J* = 8.4, 7.0 Hz, 2H, Ph), 6.26 (dd, *J* = 5.8, 2.0 Hz, 1H, H3), 5.48 (ddd, *J* = 5.7, 2.0, 1.6 Hz, 1H, H5), 3.98 (qd, *J* = 7.2, 5.6 Hz, 1H, H1′), 1.18 (d, *J* = 7.2 Hz, 3H, Me).

^{13}C DEPTq-135 NMR (101 MHz, $CDCl_3$): δ 200.4 (Cq, C2′), 172.1 (Cq, C2), 155.0 (CH, C4), 135.3 (Cq, Ph), 133.9 (CH, Ph), 129.0 (2C, CH, Ph), 128.5 (2C, CH, Ph), 123.0 (CH, C3), 83.2 (CH, C5), 43.6 (CH, C1′), 12.5 (CH_3, Me).

Preparation of 5-(2-methyl-1-oxo-1-phenylpropan-2-yl)furan-2(5*H*)-one (3an)

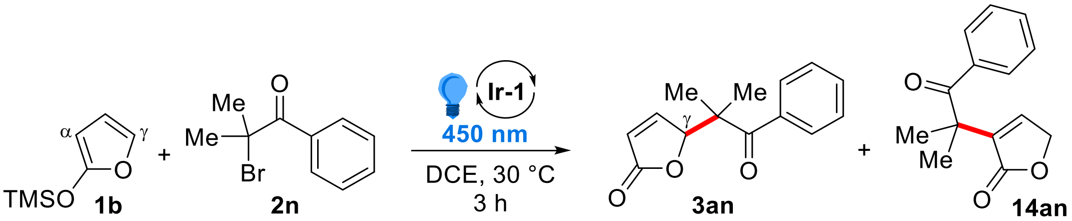

Compound (±)-**3an** was prepared according to Representative Procedure A using: TMSOF **1b** (24 mg, 0.15 mmol, 1.5 equiv), 2-bromo-2-methylpropiophenone **2n** (22.7 mg, 0.1 mmol, 1.0 equiv), **Ir-1** (1.3 mg, 0.002 mmol, 0.025 equiv), in degassed 1,2-DCE (1.7 mL, 0.06 M), at 30 °C for 3 h. The crude was purified by silica-gel flash chromatography (70/30 Petroleum Ether:EtOAc) to yield 5-(2-methyl-1-oxo-1-phenylpropan-2-yl)furan-2(5*H*)-one (**3an**, 9.2 mg, 40% isolated yield) as a yellow amorphous solid, and the α-regioisomer **14an** (7 mg, 30% isolated yield, 70% combined yield) as a yellow amorphous solid.

Data for (±)-**3an**:

TLC: R_f = 0.33 (70:30 Petroleum ether/EtOAc)

^{1}H NMR (600 MHz, $CDCl_3$): δ 7.77 – 7.71 (m, 2H, Ph), 7.57 (dd, *J* = 5.8, 1.5 Hz, 1H, H4), 7.54 (d, *J* = 7.4 Hz, 1H, Ph), 7.50 – 7.43 (m, 2H, Ph), 6.22 (dd, *J* = 5.8, 2.0 Hz, 1H, H3), 5.53 (t, *J* = 1.8 Hz, 1H, H5), 1.54 (s, 3H, Me), 1.29 (s, 3H, Me).

^{13}C NMR (101 MHz, $CDCl_3$) δ 206.0 (Cq, C2′), 172.8 (Cq, C2), 154.7 (CH, C4), 137.4 (Cq, Ph), 131.8 (CH, Ph), 128.4 (2C, CH, Ph), 127.9 (2C, CH, Ph), 123.0 (CH, C3), 87.3 (CH, C5), 50.5 (Cq, C1′), 23.0 (CH_3, Me), 20.0 (CH_3, Me).

MS (ESI, 100eV):	Calculated.:	[M+Na$^+$]: 253.1
	Found:	[M+Na$^+$]: 253.1

Data for (±)-**14an**:

TLC: R_f = 0.22 (80:20 Petroleum ether/EtOAc)

^{1}H NMR (400 MHz, $CDCl_3$): δ 7.79 – 7.72 (m, 2H, Ph), 7.48 (m, 1H, Ph), 7.38 (dd, J = 8.3, 6.9 Hz, 2H, Ph), 7.34 (t, J = 1.8 Hz, 1H, H4), 4.86 (d, J = 1.8 Hz, 2H, H5), 1.65 (s, 6H, Me).

^{13}C NMR (101 MHz, $CDCl_3$): δ 202.0 (Cq, C2′), 171.9 (Cq, C2), 143.5 (CH, C4), 140.2 (Cq, Ph), 136.9 (Cq, C3), 131.9 (CH, Ph), 128.4 (2C, CH, Ph), 128.2 (2C, CH, Ph), 70.1 (CH_2, C5), 46.8 (Cq, C1′), 25.6 (2C, CH_3, Me).

Preparation of (±)-5-(2-oxobutyl)furan-2(5*H*)-one (3ao)

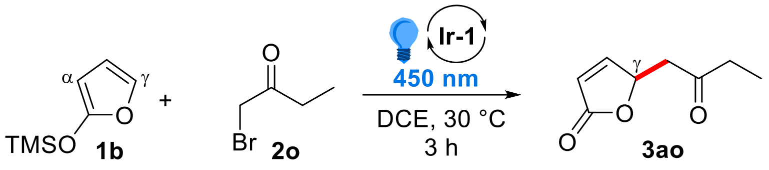

Compound (±)-**3ao** was prepared according to Representative Procedure A using: TMSOF **1b** (24 mg, 0.15 mmol, 1.5 equiv), 1-bromobutan-2-one (**2o**, 15.0 mg, 0.1 mmol, 1.0 equiv), **Ir-1** (1.3 mg, 0.002 mmol, 0.025 equiv), in degassed 1,2-DCE (1.7 mL, 0.06 M), at 30 °C for 3 h. The crude was concentrated and then purified by silica-gel flash chromatography (70/30 Petroleum Ether:EtOAc) to yield pure 5-(2-oxobutyl)furan-2(5*H*)-one (**3ao**, 7.7 mg, 50% yield) as a yellow, amorphous solid.

Data for (±) **3ao**:

TLC: R_f = 0.35 (70:30 Petroleum ether/EtOAc)

^{1}H NMR (400 MHz, $CDCl_3$) δ 7.61 (dd, J = 5.7, 1.6 Hz, 1H, H4), 6.14 (dd, J = 5.7, 2.0 Hz, 1H, H3), 5.46 (dddd, J = 7.8, 6.8, 1.8, 1.6 Hz, 1H, H5), 3.04 (dd, J = 17.3, 6.7 Hz, 1H, H1′a), 2.68 (dd, J = 17.3, 7.6 Hz, 1H, H1′b), 2.52 (m, 2H, H3′), 1.10 (t, J = 7.3 Hz, 3H, H4′).

^{13}C NMR (101 MHz, $CDCl_3$) δ 206.95 (Cq, C2′), 172.48 (Cq, C2), 156.1 (CH, C4), 121.71 (CH, C3), 79.03 (CH, C5), 44.91 (CH_2, C1′), 36.63 (Cq, C3′), 7.46 (CH_3, C4′).

MS (ESI, 100eV): Calculated.: $[M+Na^+]$: 177.0
Found: $[M+Na^+]$: 177.1

Preparation of (±)-5-(3,3-dimethyl-2-oxobutyl)furan-2(5*H*)-one (3ap)

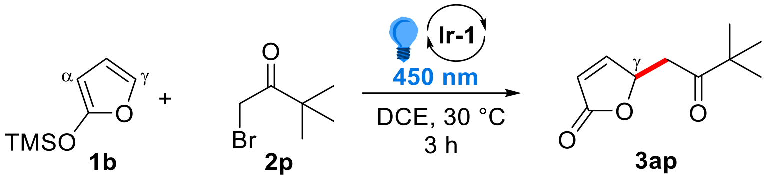

Compound (±)-**3ap** was prepared according to Representative Procedure A using: TMSOF **1b** (24 mg, 0.15 mmol, 1.5 equiv), 1-bromo-3,3-dimethylbutan-2-one (**2p**,17.8 mg, 0.1 mmol, 1.0 equiv), **Ir-1** (1.3 mg, 0.002 mmol, 0.025 equiv), in degassed 1,2-DCE (1.7 mL, 0.06 M), at 30 °C for 3 h. The crude was concentrated and then purified by silica-gel flash chromatography (70/30 Petroleum Ether:EtOAc) to yield pure 5-(3,3-dimethyl-2-oxobutyl)furan-2(5*H*)-one (**3ap**, 13.7 mg, 75% yield) as a yellow, amorphous solid.

Data for (±) **3ap**:

TLC: R_f = 0.35 (70:30 Petroleum ether/EtOAc)

^{1}H NMR (400 MHz, $CDCl_3$) δ 7.61 (dd, *J* = 5.7, 1.5 Hz, 1H, H4), 6.13 (dd, *J* = 5.7, 2.0 Hz, 1H, H3), 5.45 (dddd, *J* = 7.8, 6.2, 1.9, 1.7 Hz, 1H, H5), 3.19 (dd, *J* = 17.7, 6.1 Hz, 1H, H1′a), 2.68 (dd, *J* = 17.6, 7.7 Hz, 1H, H1′a), 1.16 (s, 9H, CH_3).

^{13}C NMR (100 MHz, $CDCl_3$) δ 211.8 (Cq, C2′), 172.5 (Cq, C2), 156.4 (CH, C4), 121.6 (CH, C3), 79.5 (CH, C5), 44.3 (Cq, C3′), 39.9 (CH_2, C1′), 26.1 (3C, CH_3).

MS (ESI, 100eV):	Calculated.:	[M+Na$^+$]: 205.1
	Found:	[M+Na$^+$]: 205.1

Preparation of (±)-5-methyl-5-(2-oxo-2-phenylethyl)furan-2(5*H*)-one (3ca)

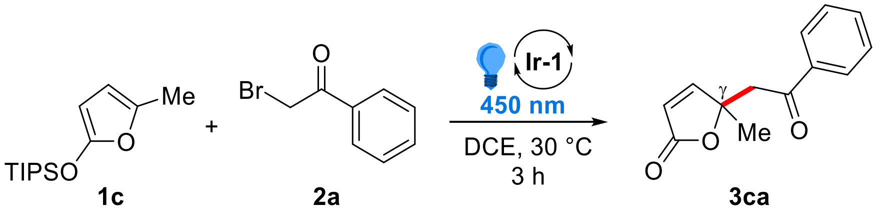

Compound (±)-**3ca** was prepared according to Representative Procedure A using: γ-Me-TIPSOF **1c** (38 mg, 0.15 mmol, 1.5 equiv), phenacyl bromide **2a** (20 mg, 0.1 mmol, 1.0 equiv), **Ir-1** (1.3 mg, 0.002 mmol, 0.025 equiv), in degassed 1,2-DCE (1.7 mL, 0.06 M), at 30 °C for 3 h. The crude was purified by silica-gel flash chromatography (70/30 Petroleum Ether:EtOAc) to yield pure 5-methyl-5-(2-oxo-2-phenylethyl)furan-2(5*H*)-one (**3ca**, 12 mg, 55% yield) as a yellow oil.

Data for (±) (**3ca**):

TLC: R_f = 0.4 (70:30 Petroleum ether/EtOAc)

^{1}H NMR (600 MHz, $CDCl_3$): δ 7.94–7.87 (m, 3H, Ph, H4), 7.63–7.57 (m, 1H, Ph), 7.51–7.44 (m, 2H, Ph), 6.03 (d, J = 5.7 Hz, 1H, H3), 3.73 (d, J = 17.1 Hz, 1H, H1′a), 3.25 (d, J = 17.0 Hz, 1H, H1′b), 1.64 (s, 3H, Me).

^{13}C NMR (150 MHz, $CDCl_3$): δ 196.1 (Cq, C2'), 171.9 (Cq, C2), 160.5 (CH, C4), 136.5 (Cq, Ph), 133.9 (CH, Ph), 128.9 (2C, CH, Ph), 128.2 (2C, CH, Ph), 120.4 (CH, C3), 87.2 (Cq, C5), 47.6 (CH_2, C1′), 24.0 (CH_3, Me).

HR-MS (ESI) Calcd.: *m/z* 217.0865 [$C_{13}H_{12}O_3$+ H]$^+$ - Found: *m/z* 217.0862 [M + H]$^+$
Δppm ±SD: 1.23 ± 1.16

Calcd.: *m/z* 239.0684 [$C_{13}H_{12}O_3$+ Na]$^+$ - Found: *m/z* 239.0689 [M + Na]$^+$
Δppm ±SD: 2.09 ± 0.00

Preparation of (±)-3-methyl-5-(2-oxo-2-phenylethyl)furan-2(5*H*)-one (3da)

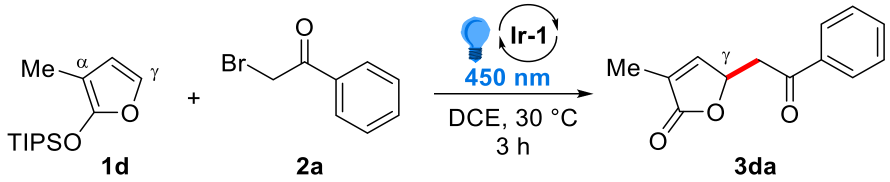

Compound (±)-**3da** was prepared according to Representative Procedure A using: α-Me-TIPSOF **1d** (38 mg, 0.15 mmol, 1.5 equiv), phenacyl bromide **2a** (20 mg, 0.1 mmol, 1.0 equiv), **Ir-1** (1.3 mg, 0.002 mmol, 0.025 equiv), in degassed 1,2-DCE (1.7 mL, 0.06 M), at 30 °C for 3 h. The crude was purified by silica-gel flash chromatography (70/30 Petroleum Ether:EtOAc) to yield pure (±)-3-methyl-5-(2-oxo-2-phenylethyl)furan-2(5*H*)-one (**3da**, 14 mg, 65% yield) as a yellow oil.

Data for (±)-(**3da**):

TLC: R_f = 0.42 (70:30 Petroleum ether/EtOAc)

^{1}H NMR (600 MHz, $CDCl_3$): δ 7.94–7.88 (m, 2H, Ph), 7.64–7.56 (m, 1H, Ph), 7.51–7.45 (m, 2H, Ph), 7.29 (t, J = 1.7 Hz, 1H, H4), 5.48 (ddt, J = 7.8, 5.9, 1.9 Hz, 1H, H5), 3.65 (dd, J = 17.4, 5.8 Hz, 1H, H1′a), 3.09 (dd, J = 17.4, 8.3 Hz, 1H, H1′b), 1.92 (t, J = 1.8 Hz, 3H).

^{13}C NMR (150 MHz, $CDCl_3$): δ 196.1 (Cq, C2'), 173.8 (Cq, C2), 148.8 (CH, C4), 144.9 (Cq, Ph), 133.9 CH, Ph), 130.5 (Cq, C3), 128.9 (2C, CH, Ph), 128.2 (2C, CH, Ph), 70.0 (CH, C5), 42.2 (CH_2, C1′), 10.7 (CH_3, Me).

MS (ESI, 100eV): Calculated.: [M+Na+]: 239.0
Found: [M+Na+]: 239.0

4.6 Photoinduced ε-alkylation of extended silyloxyfurans 1e and 1f

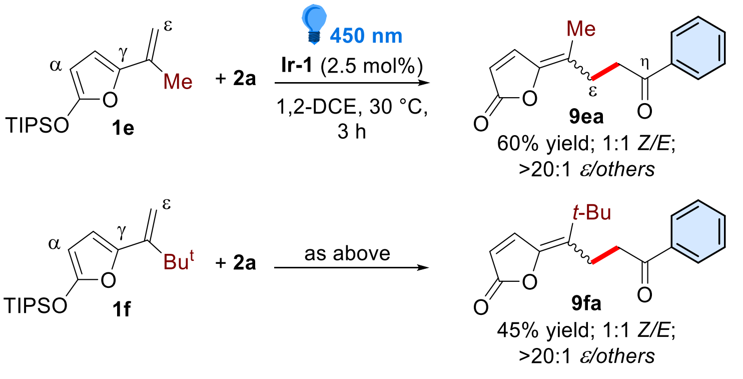

Scheme S1. Photoinduced ε-alkylation of silyloxyfurans **1e** and **1f.**

Preparation of *Z*- and *E*-5-(5-oxo-5-phenylpentan-2-ylidene)furan-2(5*H*)-one (9ea)

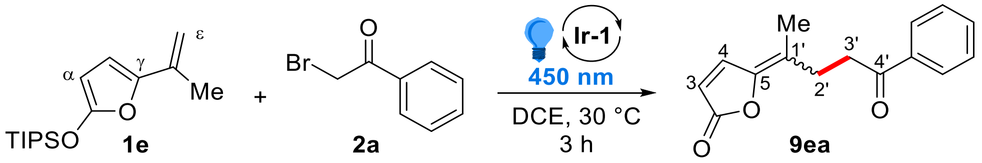

Compound **9ea** was prepared according to Representative Procedure A using triisopropyl((5-(prop-1-en-2-yl)furan-2-yl)oxy)silane (**1e**, 42 mg, 0.15 mmol, 1.5 equiv), phenacyl bromide **2a** (20 mg, 0.1 mmol, 1.0 equiv), **Ir-1** (1.3 mg, 0.002 mmol, 0.025 equiv), in degassed 1,2-DCE (1.7 mL, 0.06 M), at 30 °C for 3 h. The crude was purified by silica-gel flash chromatography (70/30 Petroleum Ether:EtOAc) to afford a first isomer (either *Z* or *E*) of 5-(5-oxo-5-phenylpentan-2-ylidene)furan-2(5*H*)-one (**9ea**, 4.5 mg) and a second geometrical isomer (4.5 mg, 60% combined yield), as white amorphous solids.

Data for **9ea** (Isomer 1)

TLC: R_f= 0.5 (70:30 Petroleum ether/EtOAc)

^{1}H NMR (400 MHz, $CDCl_3$): δ 7.99–7.94 (m, 2H, Ph), 7.77 (d, *J* = 5.5 Hz, 1H, H4), 7.64–7.58 (m, 1H, Ph), 7.53–7.48 (m, 2H, Ph), 6.15 (d, *J* = 5.5 Hz, 1H, H1′), 3.21 (t, *J* = 7.3 Hz, 2H, H3′a, H3′b), 2.77 (t, *J* = 7.3 Hz, 2H, H2′a, H2′b), 2.07 (s, 3H, Me).

^{13}C DEPTq-135 NMR (101 MHz, $CDCl_3$): δ 139.9 (CH, C4), 133.5 (CH, Ph), 128.8 (2C, CH, Ph), 128.0 (2C, CH, Ph), 118.4 (CH, C3), 36.5 (CH_2, C3′), 26.7 (CH_2, C2′), 16.8 (CH_3, Me).

Data for **9ea** (Isomer 2)

TLC: R_f = 0.41 (70:30 Petroleum ether/EtOAc)

^{1}H NMR (400 MHz, $CDCl_3$): δ 8.01–7.96 (m, 2H, Ph), 7.64 (d, *J* = 5.5 Hz, 1H, H4), 7.62–7.55 (m, 1H, Ph), 7.52–7.46 (m, 2H, Ph), 6.15 (d, *J* = 5.5 Hz, 1H, H3), 3.26 (t, *J* = 7.5 Hz, 2H, H3′a, H3′b), 2.85 (t, *J* = 7.5 Hz, 2H, H2′a, H2′b), 2.05 (s, 3H, Me).

^{13}C DEPTq-135 NMR (101 MHz, $CDCl_3$): δ 139.8 (CH, C4), 133.3 (CH, Ph), 128.7 (2C, CH, Ph), 128.1 (2C, CH, Ph), 118.4 (CH, C3), 36.7 (CH_2, C3′), 29.7 (CH_2, C2′), 17.3 (CH_3, Me).

HR-MS (ESI) Calcd.: m/z 243.1021 [$C_{15}H_{14}O_3$+ H]$^+$;
Found: m/z 243.1024 [M + H]$^+$
Δppm ±SD: 1.10 ± 0.95

Calcd.: m/z 265.0841 [$C_{15}H_{14}O_3$+ Na]$^+$;
Found: m/z 265.0847 [M + Na]$^+$
Δppm ±SD: 2.39 ± 0.79

Preparation of 5-(2,2-dimethyl-6-oxo-6-phenylhexan-3-ylidene)furan-2(5*H*)-one (9fa)

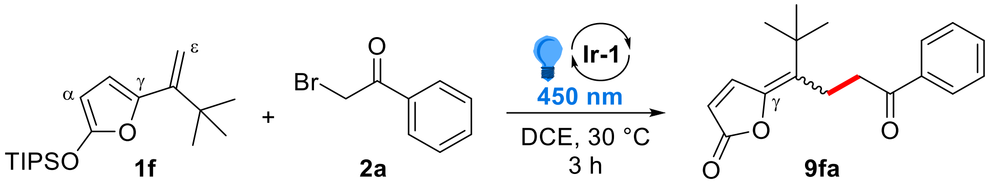

Compound **9fa** was prepared according to Representative Procedure A using ((5-(3,3-dimethylbut-1-en-2-yl)furan-2-yl)oxy)triisopropylsilane (**1f**, 48 mg, 0.15 mmol, 1.5 equiv), phenacyl bromide **2a** (20 mg, 0.1 mmol, 1.0 equiv), **Ir-1** (1.3 mg, 0.002 mmol, 0.025 equiv), in degassed 1,2-DCE (1.7 mL, 0.06 M), at 30 °C for 3 h. The crude was purified by silica-gel flash chromatography (70/30 Petroleum Ether:EtOAc) to afford an inseparable 1:1 *Z/E* mixture of 5-(2,2-dimethyl-6-oxo-6-phenylhexan-3-ylidene)furan-2(5*H*)-one (**9fa**, 45% combined yield), as a yellow oil.

Data for **9fa** (Isomer 1 + Isomer 2* – data extrapolated from spectra of the mixture):

TLC: R_f= 0.24 (90:10 Petroleum ether/EtOAc)

^{1}H NMR (400 MHz, $CDCl_3$): δ 8.04–7.99 (m, 2H, Ph), 7.99–7.94 (m, 2H, Ph*), 7.92 (d, *J* = 5.7 Hz, 1H, H4), 7.72 (d, *J* = 5.6 Hz, 1H, H4*), 7.62–7.55 (m, 2H, Ph, Ph*), 7.52–7.46 (m, 4H, Ph, Ph*), 6.17 (d, *J* = 5.7 Hz, 1H, H3), 6.09 (d, *J* = 5.5 Hz, 1H, H3*), 3.29 – 3.22 (m, 2H, H3′a, H3′b), 3.16 (m, 2H, H3′a*, H3′b*), 2.92 – 2.85 (m, 2H, H2′a, H2′b), 2.81 (m, 2H, H2′a*, H2′b*), 1.37 (s, 9H, *t*-Bu*), 1.34 (s, 9H, *t*-Bu).

^{13}C DEPTq-135 NMR (101 MHz, $CDCl_3$): δ 141.8 (CH, C4), 141.7 (CH, C4*), 133.5 (CH, Ph*), 133.3 (CH, Ph), 128.8 (2C, CH, Ph*), 128.6 (2C, CH, Ph), 128.2 (2C, CH, Ph), 128.0 (2C, CH, Ph*), 118.6 (CH, C3), 118.4 (CH, C3*), 38.8 (CH_2, C3′), 40.2 (CH_2, C3′*), 25.1 (CH_2, C2′), 23.7 (CH_2, C2′*), 30.9 (CH_3, *t*-Bu), 30.1 (CH_3, *t*-Bu*).

HR-MS (ESI) Calcd.: *m/z* 285.1491 [$C_{13}H_{12}O_3$+ H]$^+$;
Found: *m/z* 285.1496 [M + H]$^+$
Δppm ±SD: 1.64 ± 0.20

5. Late-Stage Functionalization of Butenolide 3aa

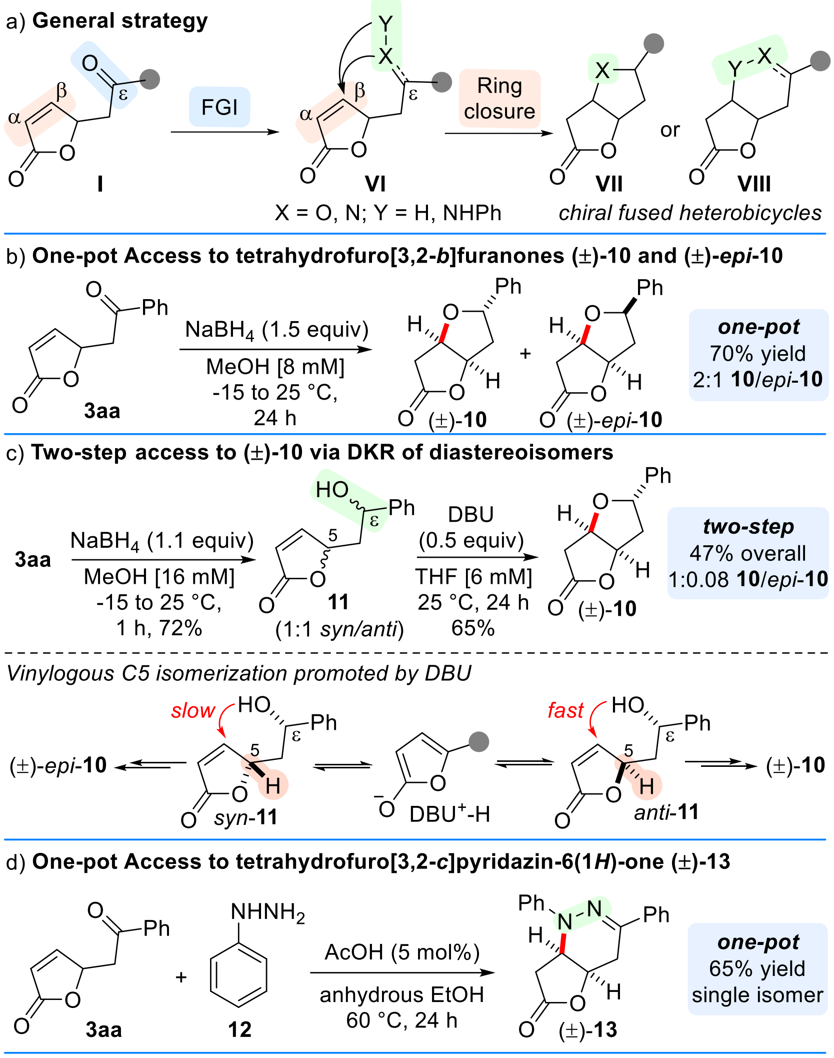

Scheme S2. a) General strategy for late-stage modification of butenolides **I**. b) One-pot access to bicyclic furanones (±)-**10** and (±)-*epi*-**10**. c) Two-step access to (±)-**10** via dynamic kinetic resolution of diastereomers *syn*-**11** and *anti*-**11**. d) One-pot access to bicyclic pyridazine-6(1*H*)-one (±)-**13**. DKR = dyanamic kinetic resolution.

5.1 One-Pot access to tetrahydrofuro[3,2-*b*]-furanones (±)-10 and (±)-*epi*-10

To demonstrate the versatility of the targeted ε-ketobutenolides **3** as strategic platforms to forge fused heterobicyclic scaffolds such as tetrahydrofuro[3,2-*b*]-furanones **10** and *epi*-**10**, we envisaged a two-step strategy centered upon the chemoselective interconversion of the ε-ketone carbonyl within **3aa** to the corresponding secondary alcohol, followed by ring closure to the β-position of the electron-deficient unsaturated lactone. At first, we evaluated the feasibility of this strategy by testing a one-pot procedure on scaffold **3aa** (Table S8).

Table S8. Chemoselective reduction of the ketone moiety within butenolide **3aa**.

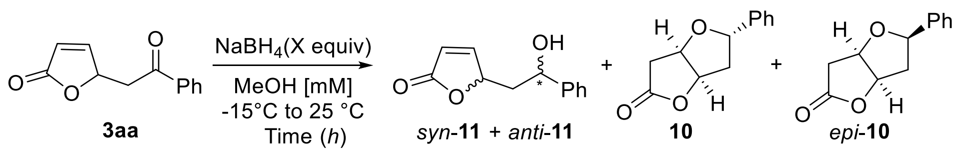

entry	NaBH4 (equiv)	[**3aa**] (mM)	Time (h)	Yield **11**[a]	d.r.[b] *syn*-**11**:*anti*-**11**	Yield[a] (**10**:*epi*-**10**)[d]	d.r.[b] (**10**:*epi*-**10**)[c]
1	1.5	8	24	nd	nd	70%	2:1
2	1.5	32	16	62%	1:1	10%	1:1
3	1.5	16	1	68	1:1	>5%	n.d.
4	1.1	16	1	72%	1:1	<5%	n.d.

[a] Isolated, combined yield of *syn*-**11** + *anti*-**11**.
[b] Determined by ^{1}H NMR of the crudes.
[c] Isolated, combined yield of **10** + *epi*-**10**. n.d. = not determined.

Indeed, treating ketone **3aa** with slight excess $NaBH_4$ (1.5 equiv) in MeOH, at −15 °C to 25 °C for 24 h, failed to deliver the expected alcohols, ending up directly to the formation of a 2:1 mixture of the *cis*-fused dioxabicycles (±)-**10** as the major product with its epimer (±)-*epi*-**10**, in a good 70% combined, isolated yield (Table S8, entry 1). Conversely, running the reaction in more concentrated solutions (32 or 16 mM) inhibited the formation of the bicycles in favor of the alcohols (±)-**11** (entries 2-4). At best, treating **3aa** with slight excess $NaBH_4$ (1.1 equiv) for 1 h at a concentration of 16 mM afforded a 1:1 *syn*/*anti* mixture of alcohols (±)-**11** in a good 72% combined, isolated yield (Table S8, entry 4).

Preparation of tetrahydrofuro[3,2-*b*]-furanones (±)-10 and (±)-*epi*-10 via a one-pot procedure (Table S8, entry 1)

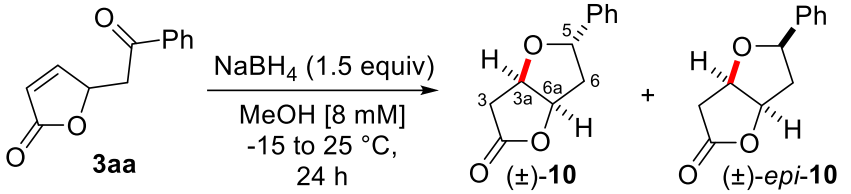

In a 50 mL flamed, round-bottom flask, ketobutenolide **3aa** (50 mg, 0.25 mmol, 1.0 equiv) was dissolved in 30 mL of dry methanol [8 mM], and the resulting solution was then kept at –15 °C in an ice-salt mixture bath under an inert argon atmosphere. After 15 min, sodium borohydride (14 mg, 0.37 mmol, 1.5 equiv) was added. The resulting suspension was stirred for 1 hour, then the temperature was allowed to rise from –15 °C to room temperature. After 24 h, the reaction was quenched with 15 mL of a saturated aqueous solution of ammonium chloride, then extracted with ethyl acetate (3×10 mL). The organic layers were collected, dried with anhydrous sodium sulfate, filtered, and concentrated under reduced pressure. The crude product was purified by silica gel flash chromatography (20:80 petroleum ether/diethyl ether) to afford (±)-**10** (24 mg) as and (±)-*epi*-**10** (12 mg) in a 70% isolated, combined yield as light-yellow solids.

Data for (±)-**10**:

TLC: R_f= 0.61 (diethyl ether)

^{1}H NMR (400 MHz, $CDCl_3$): δ 7.45–7.30 (m, 5H, Ph), 5.27 (t, *J* = 4.6 Hz, 1H, H6a), 5.15 (dd, *J* = 10.6, 5.0 Hz, 1H, H5), 5.08 (ddd, *J* = 6.0, 4.4, 1.5 Hz, 1H, H3a), 2.88 (dd, *J* = 18.8, 6.0 Hz, 1H, H3α), 2.79 (bd, *J* = 18.8, 1.1 Hz, 1H, H3β), 2.74 (dd, *J* = 14.0, 4.9 Hz, 1H, H6β), 2.06 (ddd, *J* = 14.0, 10.6, 4.8 Hz, 1H, H6α).

^{13}C NMR (100 MHz, $CDCl_3$): δ 175.5 (Cq, C2), 139.9 (Cq, Ph), 128.6 (2C, CH, Ph), 128.1 (CH, Ph), 125.8 (2C, CH, Ph), 84.8 (CH, C6a), 79.7 (CH, C5), 78.3 (CH, C3a), 41.6 (CH_2, C6), 36.7 (CH_2, C3).

HR-MS (ESI) Calcd.: m/z 205.0865 $[C_{12}H_{12}O_3 + H]^+$;
Found: m/z 205.0867 $[M + H]^+$
Δppm ±SD: 0.98 ± 0.98

The relative configuration of (±)-**10** was determined by ^{1}H-^{1}H NOESY NMR experiments (400 MHz, $CDCl_3$, Figure S3).

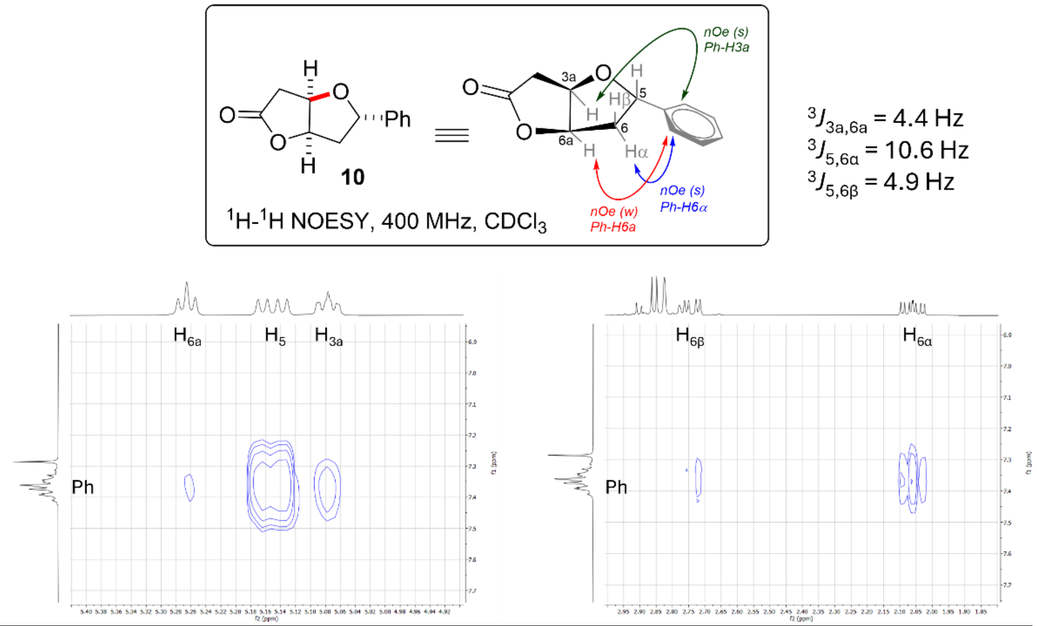

Figure S3. Diagnostic ^{1}H-^{1}H NOESY signals to assess the relative configuration of compound **10**.

On these bases we identified compound **10** as *rac*-(3a*R*,5*S*,6a*R*)-5-phenyltetrahydrofuro[3,2-*b*]furan-2(3*H*)-one (**10**).

Data for (±)-*epi*-**10**:

TLC: R_f= 0.37 (diethyl ether)

^{1}H NMR (400 MHz, $CDCl_3$): δ 7.42–7.30 (m, 5H, Ph), 5.17 (ddd, *J* = 6.9, 4.5, 2.3 Hz, 1H, H6a), 5.00 (dd, *J* = 8.4, 7.4 Hz, 1H, H5), 4.72 (ddd, *J* = 5.5, 4.6, 1.3 Hz, 1H, H3a), 2.90 (dd, *J* = 18.4, 1.3 Hz, 1H, H3β), 2.84 (dd, *J* = 18.4, 5.5 Hz, 1H, H3α), 2.82 (ddd, *J* = 14.5, 7.4, 6.9 Hz, 1H, H6α), 2.29 (ddd, *J* = 14.5, 8.4, 2.4 Hz, 1H, H6β).

^{13}C NMR (100 MHz, CDCl3):175.3 (Cq, C2), 139.9 (Cq, Ph), 128.6 (2C, CH, Ph), 128.2 (CH, Ph), 126.1 (2C, CH, Ph), 84.5 (CH, C6a), 81.7 (CH, C5), 78.9 (CH, C3a), 41.0 (CH_2, C6), 36.1 (CH_2, C3).

The relative configuration of compound **(±)-*epi*-10** was corroborated by ^{1}H-^{1}H NOESY NMR experiments (400 MHz, $CDCl_3$, Figure S4).

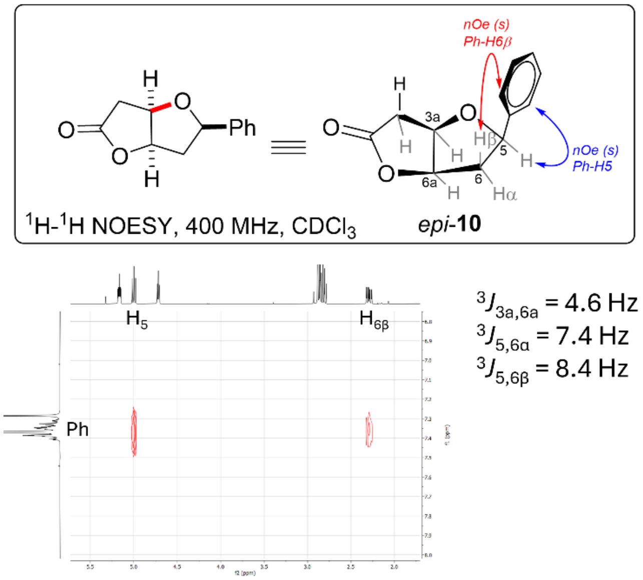

Figure S4. Diagnostic ^{1}H-^{1}H NOESY signals to assess the relative configuration of compound *epi*-**10**.

On these bases we identifed compound *epi*-**10** as *rac*-(3a*R*,5*R*,6a*R*)-5-phenyltetrahydrofuro[3,2-*b*]furan-2(3*H*)-one.

Preparation of 5-(2-hydroxy-2-phenylethyl)furan-2(5*H*)-one (±)-*syn*-11 and (±)-*anti*-11
(Table S8, entry 4)

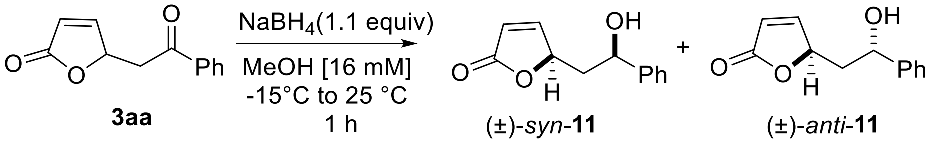

In a 50 mL flamed, round-bottom flask, ketobutenolide **3aa** (50 mg, 0.25 mmol, 1.0 equiv) was dissolved in 16 mL of dry methanol [16 mM], and the resulting solution was then kept at –15 °C in an ice-salt mixture bath under an inert argon atmosphere. After 15 min, sodium borohydride (10.5 mg, 0.27 mmol, 1.1 equiv) was added. The resulting suspension was stirred for 5 minutes, then the temperature was allowed to rise from –15 °C to room temperature. After 1 h, the reaction was quenched with 15 mL of a saturated aqueous solution of ammonium chloride, then extracted with ethyl acetate (3×10 mL). The organic layers were collected, dried with anhydrous sodium sulfate, filtered, and concentrated under reduced pressure. The crude product was purified by silica gel flash chromatography (20:80 petroleum ether/diethyl ether) to afford (±)-*syn*-**11** (18 mg) and (±)-*anti*-**11** (18 mg) in a 72% isolated, combined yield as light-yellow solids.

Data for (±)-*syn*-**11**:

TLC: R_f= 0.61 (20:80 petroleum ether/diethyl ether)

^{1}H NMR (400 MHz, $CDCl_3$): δ 7.53 (dd, *J* = 5.7, 1.5 Hz, 1H, H4), 7.39 (m, 4H, Ph), 7.3 –7.30 (m, 1H, Ph), 6.13 (dd, *J* = 5.7, 2.0 Hz, 1H, H3), 5.44 (ddt, *J* = 9.6, 3.8, 1.8 Hz, 1H, H5), 5.09 (bd, *J* = 10.2 Hz, 1H, H2′), 2.16 (ddd, *J* = 14.4, 10.4, 4.0 Hz, 1H, H1′a), 1.86 (ddd, *J* = 14.4, 9.6, 2.6 Hz, 1H, H1′b).

^{13}C DEPTq-135 NMR (100 MHz, $CDCl_3$): 172.5 (Cq, C2), 156.9 (CH, C4), 143.7 (Cq, Ph), 128.8 (2C, CH, Ph), 128.1 (CH, Ph), 125.5 (2C, CH, Ph), 121.2 (CH, C3), 80.9 (CH, C5), 70.9 (CH, C2′), 42.8 (CH_2, C1′).

Data for (±)-*anti*-**11**:

TLC: R_f= 0.54 (20:80 petroleum ether/diethyl ether)

^{1}H NMR (400 MHz, $CDCl_3$): δ 7.54 (dd, *J* = 5.7, 1.5 Hz, 1H, H4), 7.39-7.43 (m, 4H, Ph), 7.33 (m, 1H, Ph), 6.13 (dd, *J* = 5.7, 2.0 Hz, 1H, H3), 4.99 (ddd, *J* = 7.8, 5.2, 1.8 Hz, 1H, H5), 4.98 (dd, *J* = 7.8, 6.0 Hz, 1H, H2′), 2.24 (dt, *J* = 14.3, 7.8 Hz, 1H, H1′a), 2.13 (ddd, *J* = 14.3, 6.0, 5.2 Hz, 1H, H1′b).

^{13}C DEPTq-135 NMR (100 MHz, $CDCl_3$): 172.3 (Cq, C2), 165.5 (CH, C4), 143.0 (Cq, Ph), 128.8 (2C, CH, Ph), 128.3 (CH, Ph), 125.9 (2C, CH, Ph), 121.1 (CH, C3), 81.1 (CH, C5), 71.4 (CH, C2′), 42.2 (CH_2, C1′).

5.2 Two-Step access to tetrahydrofuro[3,2-*b*]-furanone 10 via *oxa*-Michael cyclization of alcohol (±)-11

With an efficient procedure at hand to access alcohols (±)-**11** (Table S8, entry 4), we evaluated the possibility to obtain bicycle (±)-**10** in a more stereoselective way via base-catalysed *oxa*-Michel cyclization of (±)-**11**. Based on literature precedents,[40] several tests were carried out using DBU as the base of choice (Table S9). Initially, we started by reacting a 1:1 mixture of *syn*-**11** and *anti*-**11** with DBU (1.0 equiv) in THF at a concentration of 16 mM, at room temperature (Table S9, entry 1).

Table S9. Evaluating the *oxa*-Michael cyclization of (±)-**11**, promoted by DBU.

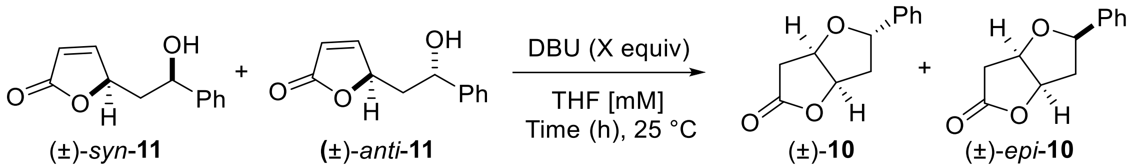

entry	*syn*-**11**:*anti*-**11**	[**11**] (mM)	DBU (equiv)	Time (h)	Yield % (**10**:*epi*-**10**)[a]	d.r. (**10**:*epi*-**10**)[b]	Overall yield from **3aa** (**10**)[c]
1	1:1	16	1.0	24	35%	2:1	12
2	1:0	16	1.0	72	60%	2:1	7
3	0:1	16	1.0	24	70%	4:1	20
4	1:0	6	0.5	72	55%	5:1	16
5	0:1	6	0.5	16	75%	12.5:1	26
6	1:1	6	0.5	24	65%[d]	10:1	47

[a] Isolated, combined yield of **10** + *epi*-**10**.
[b] Determined by ^{1}H NMR of the crudes.
[c] Overall isolated yield of (±)-**10** over the two steps from **3aa**.
[d] 25% of *syn*-**11** recovered. n.d. = not determined.

After 24 h we observed the formation of a 2:1 **10**/*epi*-**10** mixture with a combined, isolated yield of 35%. Interestingly, the same reaction carried out on the sole alcohol *syn*-**11** proved slower, and the same 2:1 **10**/*epi*-**10** mixture was obtained in a good 60% yield (entry 2). Since bicycle (±)-**10** could only be obtained from the cyclization of alcohol (±)-*anti*-**11**, it was clear that a base-catalyzed isomerization of (±)-*syn*-**11** to (±)-*anti*-**11** was here operative (see ref. [31] in the text). To corroborate these observations, we performed the reaction on the sole alcohol (±)-*anti*-**11**: indeed, under the same reaction conditions of entry 2, a 4:1 (±)-**10**/(±)-*epi*-**10** mixture was obtained in a 70% yield (Table S9, entry 3). Aiming at further enhancing diastereoselection, we performed the reaction in a more dilute solution with a sub-stoichiometric loading of the base. Gladly, running the reaction on the sole alcohol (±)-*anti*-**11** at 6 mM concentration with DBU (0.5 equiv) resulted in the formation of (±)-**10** in a good 75% isolated yield as almost the sole isomer (12.5:1 (±)-**10**/(±)-*epi*-**10**, entry 5). The best result was finally obtained by applying these conditions directly to the 1:1 mixture of alcohols (±)-**11**: indeed,

after 24 h, almost the sole bicycle (±)-**10** was obtained (10:1 (±)-**10**/(±)-*epi*-**10**, entry 6), with an overall yield from **3aa** of 47%.

Preparation of tetrahydrofuro[3,2-*b*]-furanones 10 via cyclization of (±)-11 (Table S9, entry 6)

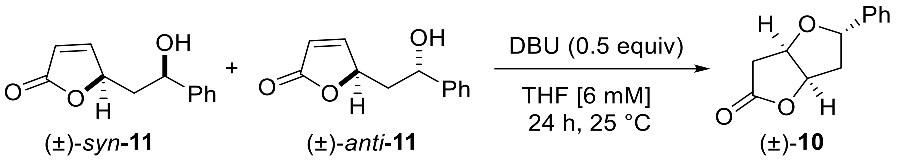

In a 100 mL flamed, round-bottom flask, a 1:1 mixture of (±)-*syn*-**11** and (±)-*anti*-**11** (50 mg, 0.25 mmol, 1.0 equiv) was dissolved in 40 mL of dry THF [6 mM], and the resulting solution was then kept at room temperature under an inert argon atmosphere. DBU (20 μL, 0.125 mmol, 0.5 equiv) was then added. The reaction was vigorously stirred at room temperature for 24 h, then it was quenched with saturated aqueous solution of ammonium chloride (15 mL) and finally extracted with ethyl acetate (3×10 mL). The organic layers were collected, dried with anhydrous sodium sulfate, filtered, and concentrated under reduced pressure. ^{1}H NMR analysis of the crude revealed the formation of a 10:1 (±)-**10**/(±)-*epi*-**10** mixture of products. The crude product was purified by silica gel flash chromatography (20:80 petroleum ether/diethyl ether) to afford (±)-**10** (33 mg, 65% yield) as a light-yellow solid.

5.3 One-pot access to tetrahydrofuro[3,2-*c*]pyridazine-6(1*H*)-one (±)-13

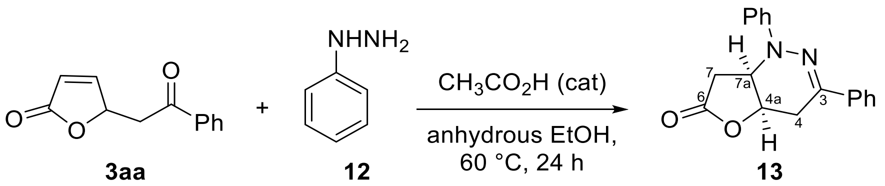

To a solution of ketobutenolide **3aa** (50 mg, 0.26 mmol, 1.0 equiv) in 2 mL of absolute ethanol (0.1 M) at room temperature under an inert argon atmosphere, phenylhydrazine (**12**, 26 μL, 0.26 mmol, 1.0 equiv) and catalytic glacial acetic acid (6 μL) were added. The yellow suspension was vigorously stirred and heated to 60 °C in a 4 mL vial. After 24 h the resulting mixture was concentrated under reduced pressure to afford a yellow solid. The crude product was purified by silica gel flash chromatography (20:80 petroleum ether/diethyl ether) to yield (±)-**13** (21.8 mg, 60%) as a single isomer, as a yellow solid.

Data for (±)-**13**:

TLC: R_f = 0.3 (70:30 Petroleum ether/EtOAc)

^{1}H NMR (400 MHz, $CDCl_3$): δ 7.81 (m, 2H, Ph), 7.47–7.36 (m, 5H, Ph), 7.35–7.29 (m, 2H, Ph), 7.04 (m, 1H, Ph), 5.31 (dt, *J* = 7.5, 4.7 Hz, 1H, H4a), 4.81 (td, *J* = 7.6, 4.8 Hz, 1H, H7a), 3.15 (dd, *J* = 16.3, 4.6 Hz, 1H, $H4_\alpha$), 3.04 (dd, *J* = 18.2, 7.7 Hz, 1H, $H7_\alpha$), 2.90 (dd, *J* = 16.4, 4.8 Hz, 1$H4_\beta$), 2.71 (dd, *J* = 18.2, 4.8 Hz, 1H, $H7_\beta$).

^{13}C DEPTq-135 NMR (100 MHz, $CDCl_3$): 173.9 (Cq, C2), 146.2 (Cq, C3), 144.6 (Cq, Ph), 136.9 (Cq, Ph), 129.3 (2C, CH, Ph), 129.0 (CH, Ph), 128.6 (2C, CH, Ph), 125.2 (2C, CH, Ph), 121.6 (CH, Ph), 115.5 (2C, CH, Ph), 76.0 (CH, C4a), 51.9 (CH, C7a), 34.3 (CH_2, C4 or C7), 26.3 (CH_2, C4 or C7).

MS (ESI, 50eV): Calcd.: [M+Na+]: 315.1
Found: [M+Na+]: 315.1

6. Control Experiments

6.1 Control experiments with TEMPO. Preparation of 1-phenyl-2-((2,2,6,6-tetramethylpiperidin-1-yl)oxy)ethan-1-one (14)

To a 5 mL vial equipped with a magnetic stir bar, TIPSOF **1a** (20 mg, 0.14 mmol, 1.0 equiv), phenacyl bromide **2a** (87 mg, 0.28 mmol, 2.0 equiv) and TEMPO (65 mg, 0.42 mmol, 3.0 equiv) were dissolved in degassed 1,2-dichoroethane (1.5 mL), at room temperature under Ar. Afterwards, a solution of **Ir-1** (2.5 mg, 0.0035 mmol, 0.025 equiv) in degassed 1,2-DCE (0.5 mL) was added via syringe. The vial was sealed with a screw-top cap with septum and then vacuumed and backfilled with argon (3 times). The vial was then sealed with Parafilm and placed in the photoreactor under 450 nm irradiation at 30 °C for 3 hours, after which it was concentrated under vacuum. ^{1}H-NMR analysis of the crude (trimethylorthoformate was used as internal standard) revealed the formation of adduct **14** (33% NMR yield) with no traces of alkylation product **3aa** (Table S10, entry 1). The crude was finally purified by silica gel flash chromatography (Petroleum Ether:EtOAc 90:10) to afford pure 1-phenyl-2-((2,2,6,6-tetramethylpiperidin-1-yl)oxy)ethan-1-one **14** (12.3 mg, 32%) as a yellow wax. All spectroscopic (^{1}H and ^{13}C NMR) and analytical data of compound **14** fully matched those reported in literature.[41]

Table S10. TEMPO-trapping experiments.

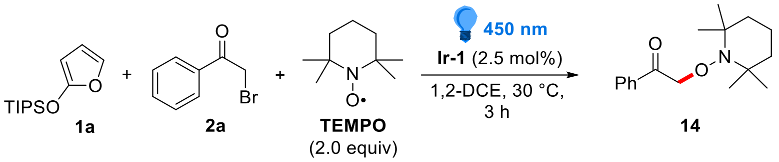

entry	**1a**:**2a**	Yield % (**14**)
1	1.5:1	32%[a]
2	1:0	*no reaction*
3	0:1	5%[b]
4[c]	1.5:1	*no reaction*
5[c]	1.5:1	*no reaction*

[a] Isolated yield.
[b] Determined by ^{1}H NMR of the crudes.
[c] Reaction carried out in the absence of **Ir-1**.
[d] Reaction carried out without blue LED irradiation.

The reaction performed on the sole **1a** proved completely unsuccessful (entry 2), while using **2a** (43.5 mg, 0.14 mmol, 1.0 equiv) and TEMPO (44 mg, 0.28 mmol, 2.0 equiv) without **1a** afforded a small quantity of compound **14** (5%), only detectable by MS and NMR (entry 3). Again, as a control experiment, running the reaction under the conditions of entry 1 but without light irradiation or without **Ir-1** did not afford any products (entries 4 and 5).

6.2 On-Off Experiment

To a 4 mL vial equipped with a magnetic stir bar and protected from light with an aluminium cover, 2-triisopropilsilyloxyfuran **1a** (TIPSOF, 36 mg, 0.15 mmol, 1.5 equiv) and phenacyl bromide **2a** (20 mg, 0.1 mmol, 1.0 equiv) were dissolved in degassed 1,2-dichloroethane-d_4 (1.0 mL) at room temperature under Ar. 1,2-Diphenylethane (18.2 mg, 0.1 mmol, 1.0 equiv) was added to the solution as an internal standard (shown to be inert under the reaction conditions and exhibiting a characteristic peak that does not overlap with other signals of interest). Afterwards, a solution of photocatalyst *fac*-Ir(ppy)$_3$ (**Ir-1**, 1.3 mg, 0.002 mmol, 0.025 equiv) in 1,2-dichloroethane-d_4 (0.7 mL) was added by a syringe. The vial was sealed with a screw-top cap with septum and then vacuumed and backfilled with argon for 3 times. A homogeneous portion of the vial contents (0.6 mL) was transferred to an NMR tube. An inert atmosphere was re-established by flushing with argon, and the tube was sealed with Parafilm. The tube was irradiated with alternating light and dark cycles in duplicate as described in Table S11 and Figure S5. At the end of every cycle of light and every cycle of dark, ^{1}H-NMR analysis of the crude (1,2-diphenylethane was used as internal standard) was performed.

Table S11. On-Off Experiment Data

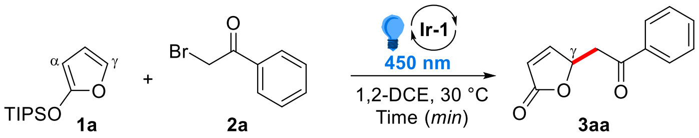

Entry	Time (min)	Light (On/Off)	NMR Yield (3aa %)
**1**	T_0 (0)	Off	0
**2**	T_1 (0-2)	On	8
**3**	T_2 (2-12)	Off	8
**4**	T_3 (12-14)	On	22
**5**	T_4 (14-24)	Off	22
**6**	T_5 (24-26)	On	28
**7**	T_6 (26-36)	Off	28
**8**	T_7 (36-40)	On	38
**9**	T_8 (40-50)	Off	38
**10**	T_9 (50-55)	On	46
**11**	T_{10} (55-65)	Off	46
**12**	T_{11} (65-75)	On	52
**13**	T_{12} (70-85)	Off	52
**14**	T_{13} (85-2965)	Off	52
**15**	T_{14} (2965-2980)	On	58

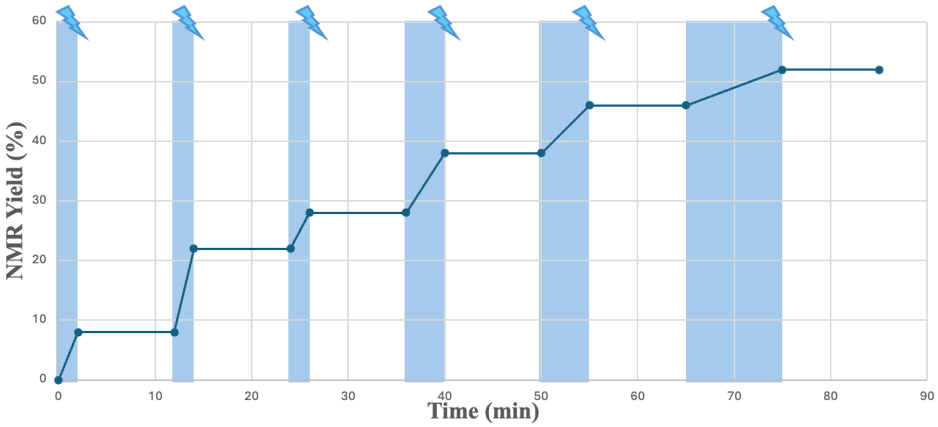

Figure S5. Plotted Time *vs* NMR Yield diagram elucidating the inertness of the reaction in the absence of light irradiation.

7. Proposed Mechanism to Access *bis*-Alkylated Adduct 8aa

Concerning the formation of the bis-alkylated adduct **8aa**, which was detected and isolated as minor product during the optimization screening described in Section 4, herein we propose a likely mechanism which could be operative in certain solvents other than 1,2-DCE, such as $CHCl_3$. In this context, the last step of the proposed catalytic cycle toward **3aa** (Scheme 4 in the main text), consisted of the desilylation of carbocation **3aa**$^+$. As depicted in Scheme S3a, either β-elimination of **3aa**$^+$, likely promoted by basic conditions (K_2CO_3), or formation of the bromo-adduct **15** followed by elimination, would lead to the γ-alkyl silyldienol ether **14**. This compound can undergo a second vinylogous alkylation with **2a** to afford the γ,γ-bisalkylated product **8aa**, through the same photocatalytic cycle promoted by Ir(ppy$_3$) (Scheme S3b).

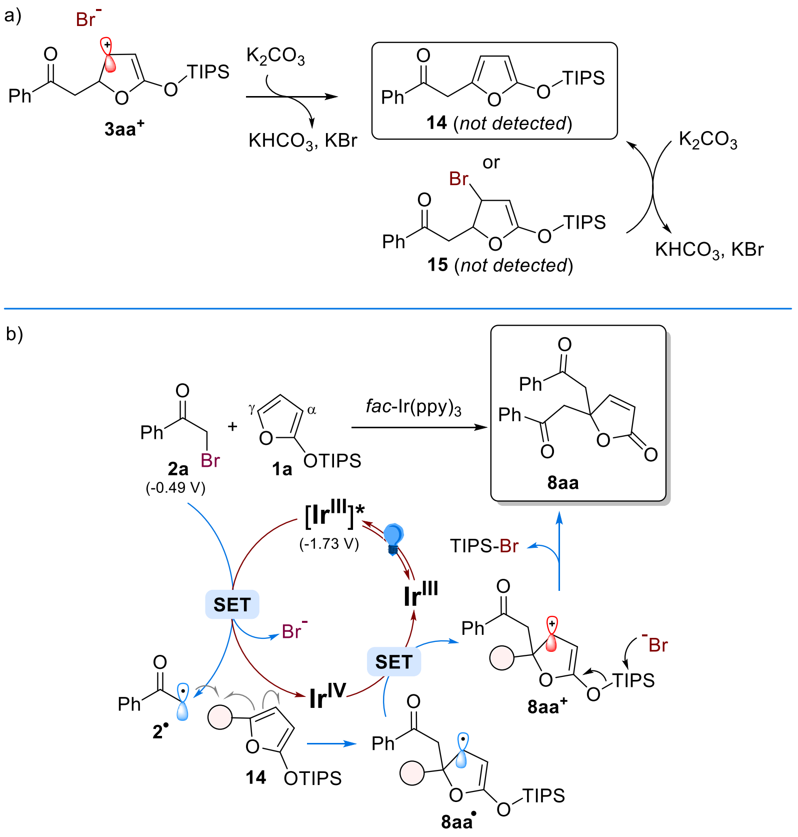

Scheme S3. a) Alternative fate of carbocation intermediate **3aa**$^+$ affording silyloxyfuran **14** possibly via either direct β-elimination or formation of bromo-derivative **15** and subsequent elimination b) Proposed catalytic cycle for the construction of *γ,γ*-bis-alkylated product **8aa**.

8. References

[37] M. Balletti, E. Marcantonio, P. Melchiorre, "*Photochemical organocatalytic enantioselective radical γ-functionalization of α-branched enals*" *Chem. Commun.* **2022**, *58*, 6072–6075.

[38] S. F. Martin, K. J. Barr, D. W. Smith, S. K. Bur, "Applications of Vinylogous Mannich Reactions. Concise Enantiospecific Total Syntheses of (+)-Croomine" *J. Am. Chem. Soc.* **1999**, *121*, 6990–6997.

[39] C. Curti, B. Ranieri, L. Battistini, G. Rassu, V. Zambrano, G. Pelosi, G. Casiraghi, F. Zanardi, "Catalytic, Asymmetric Vinylogous Mukaiyama Aldol Reactions of Pyrrole- and Furan-Based Dienoxy Silanes: How the Diene Heteroatom Impacts Stereocontrol" *Adv. Synth. Catal.* **2010**, *352*, 2011–2022.

[40] Y. Zhang, X. Liu, F. Shui, F. Zhou, J. Cui, X. Chen, "A concise synthesis of (+)-goniofufurone, (+)-7-*epi*-goniofufurone, (+)-crassalactones B and C" *Tetrahedron Lett.* **2019**, *60*, 1784–1787.

[41] N. S. Dange, A. Hussain Jatoi, F. Robert, Y. Landais, "Visible-Light-Mediated Addition of Phenacyl Bromides onto Cyclopropenes" *Org. Lett.* **2017**, *19*, 3652–3655.
