# Supplementary material for: Photoinduced, Chemoselective γ‐Alkylation of 2‐Silyloxyfurans With α‐Bromoketones: A Rapid Entry to Chiral ε‐Keto‐γ‐Butenolides
Source: Chemistry. 2025 Dec 18;32(5):e03083. doi: 10.1002/chem.202503083 (PMC12865137; doi:10.1002/chem.202503083)
Supplement: Supplementary file 2 — Supporting File 2: chem70577‐sup‐0002‐DataFile.pdf [file CHEM-32-e03083-s002.pdf]

# Electronic Supporting Information
# Part 2

## Photoinduced, Chemoselective γ-Alkylation of 2-Silyloxyfurans with α-Bromoketones: a Rapid Entry to Chiral ε-Keto-γ-Butenolides

Debora Guazzetti,† [a] Luca Aimi,† [a] Enrico Marcantonio,[a,b] Giovanni Maria Siciliano,[a] Kelly Bugatti,[a] Sara Dobani,[c] Andrea Sartori,[a] Lucia Battistini,[a] Franca Zanardi,*[a] and Claudio Curti*[a]

[a] D. Guazzetti, L. Aimi, Dr. E. Marcantonio, G. M. Siciliano, Dr. K. Bugatti, Prof. Dr. A. Sartori, Prof. Dr. L. Battistini, Prof. Dr. F. Zanardi, Prof. C. Curti
Department of Food and Drug,
University of Parma,
Parco Area delle Scienze 27A, I-43124 Parma, Italy
E-mail: claudio.curti@unipr.it, franca.zanardi@unipr.it

[b] Dr. E. Marcantonio (current address)
Department of Chemistry,
Aarhus University,
8000 Aarhus, Denmark

[c] Dr. S. Dobani
Department of Food and Drug,
University of Parma,
Via Volturno 39, I-43125 Parma, Italy

## Table of Contents

## Part 2

- 9. Representative $^{1}H$ and $^{13}C$ NMR Spectra S51

# 9. Representative $^{1}H$ and $^{13}C$ NMR Spectra

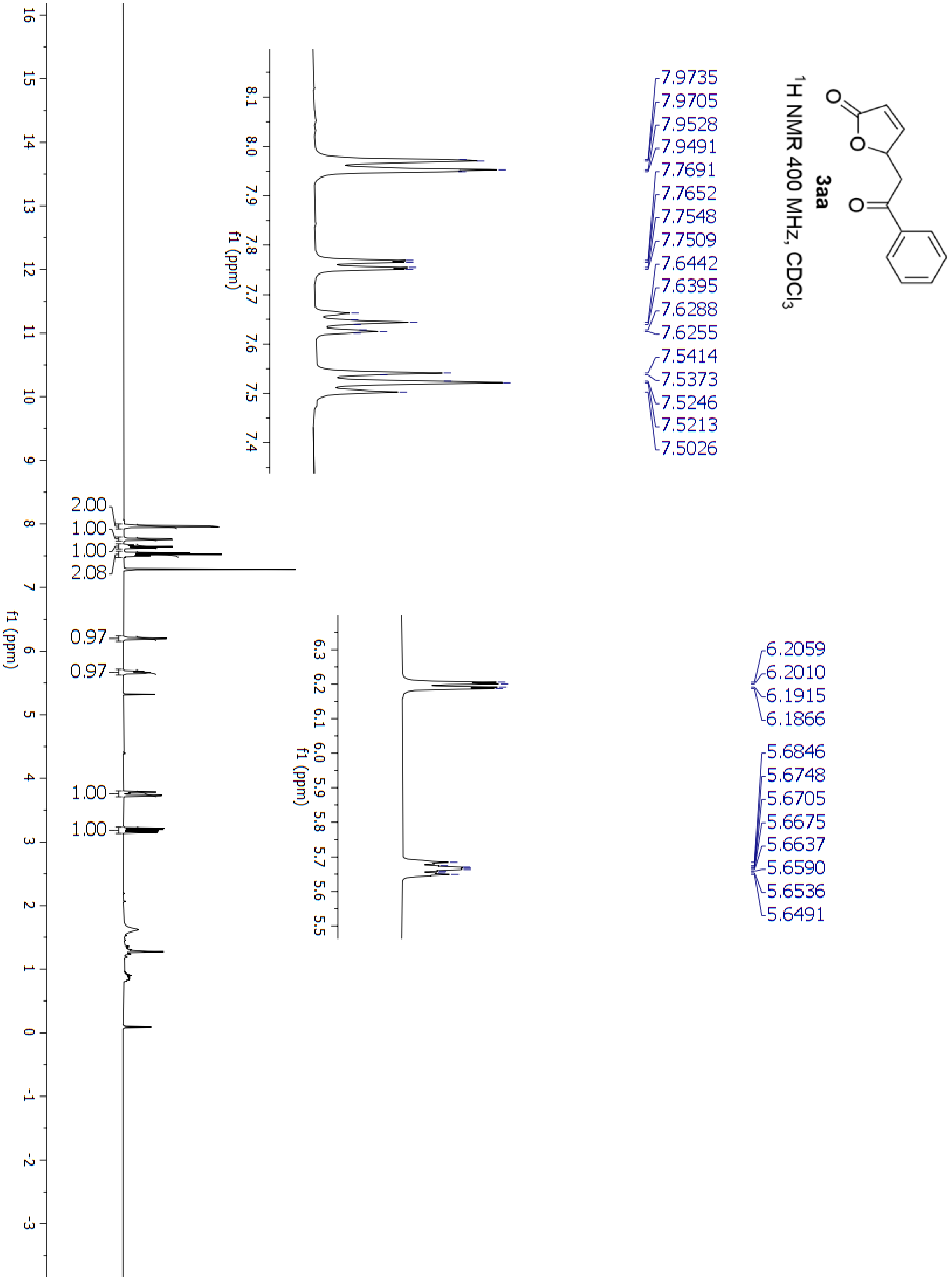

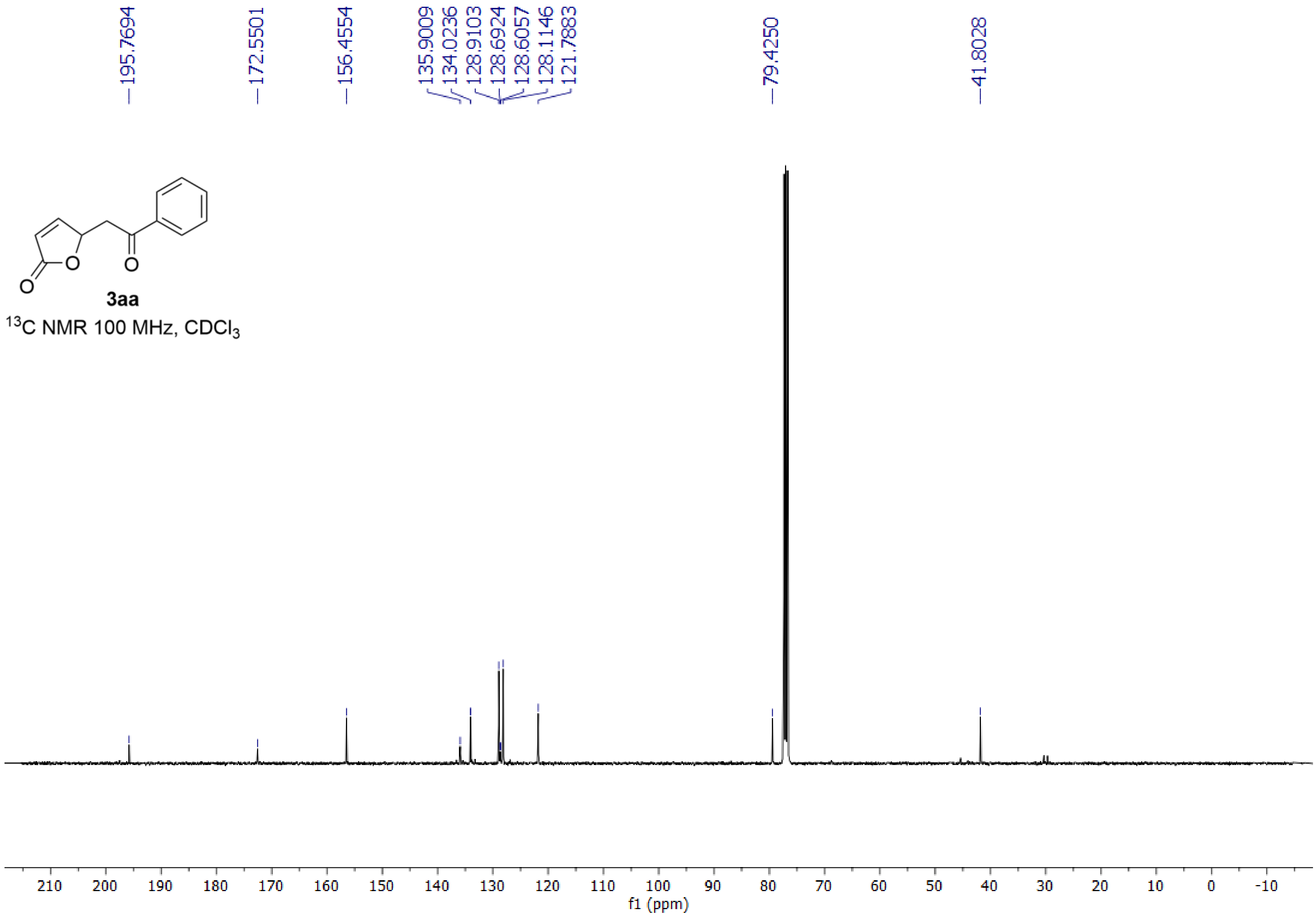
195.7694
172.5501
156.4554
135.9009
134.0236
128.9103
128.6924
128.6057
128.1146
121.7883
79.4250
41.8028
3aa
$^{13}C$ NMR 100 MHz, $CDCl_3$
210 200 190 180 170 160 150 140 130 120 110 100 90 80 70 60 50 40 30 20 10 0 -10
f1 (ppm)

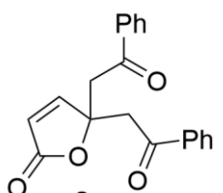

**8aa**

$^1$H NMR 400 MHz, $CDCl_3$

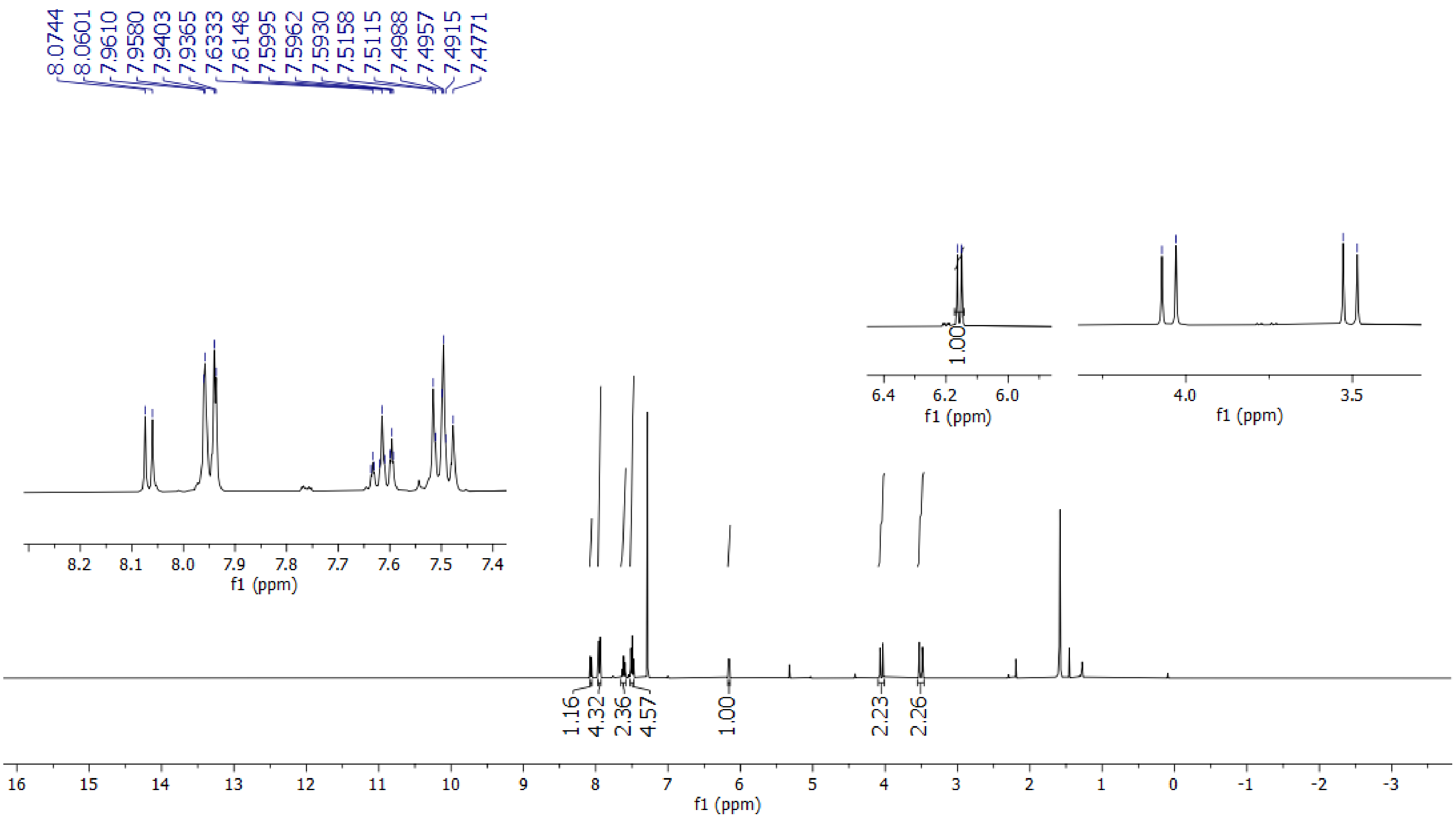

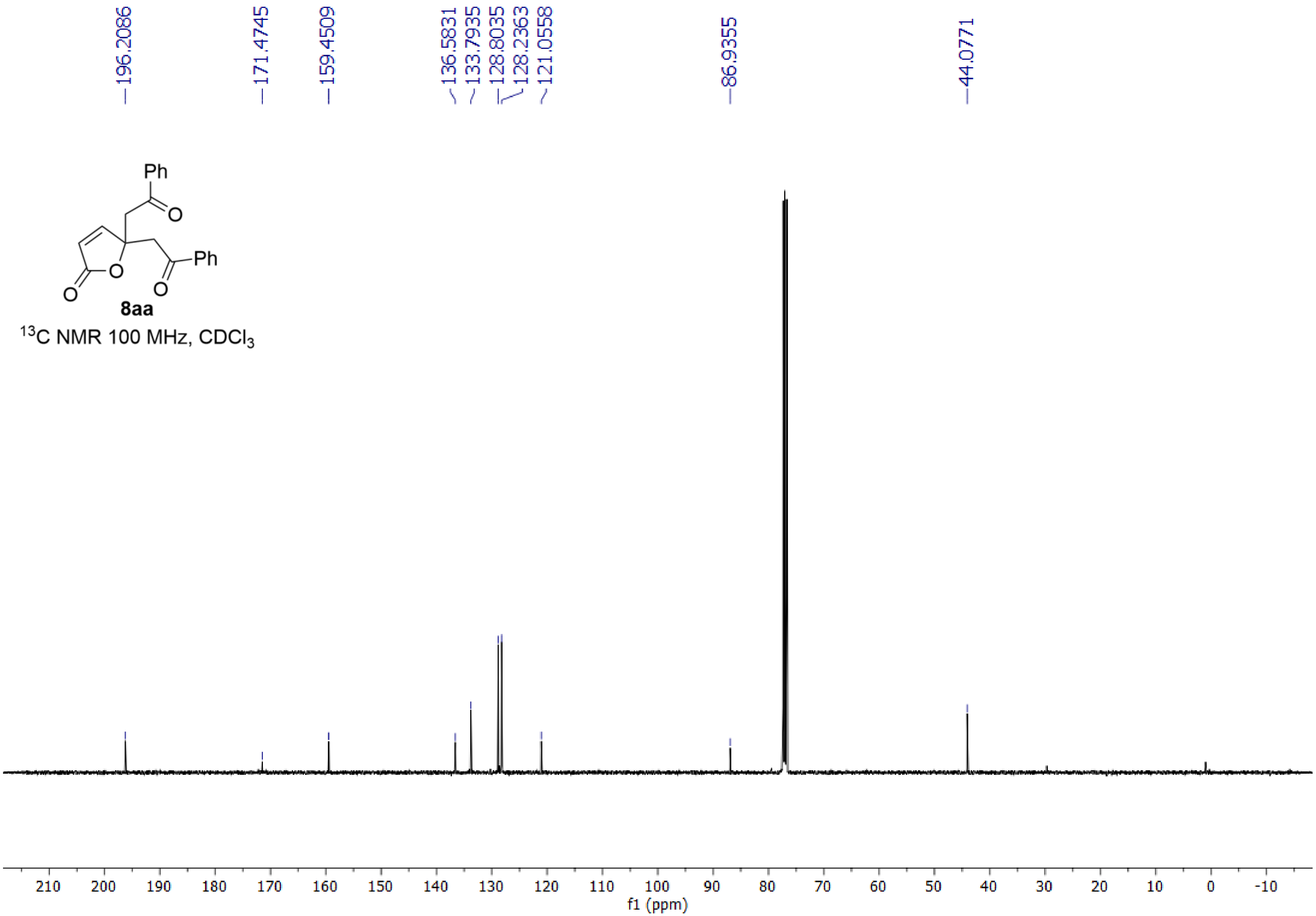
196.2086
171.4745
159.4509
136.5831
133.7935
128.8035
128.2363
121.0558
86.9355
44.0771
Ph
O
Ph
O
O
O
8aa
$^{13}$C NMR 100 MHz, $CDCl_3$
210 200 190 180 170 160 150 140 130 120 110 100 90 80 70 60 50 40 30 20 10 0 -10
f1 (ppm)

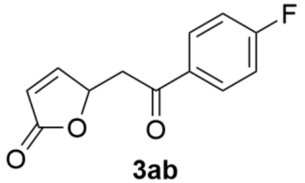

**3ab**

$^{1}H$ NMR 400 MHz, $CDCl_3$

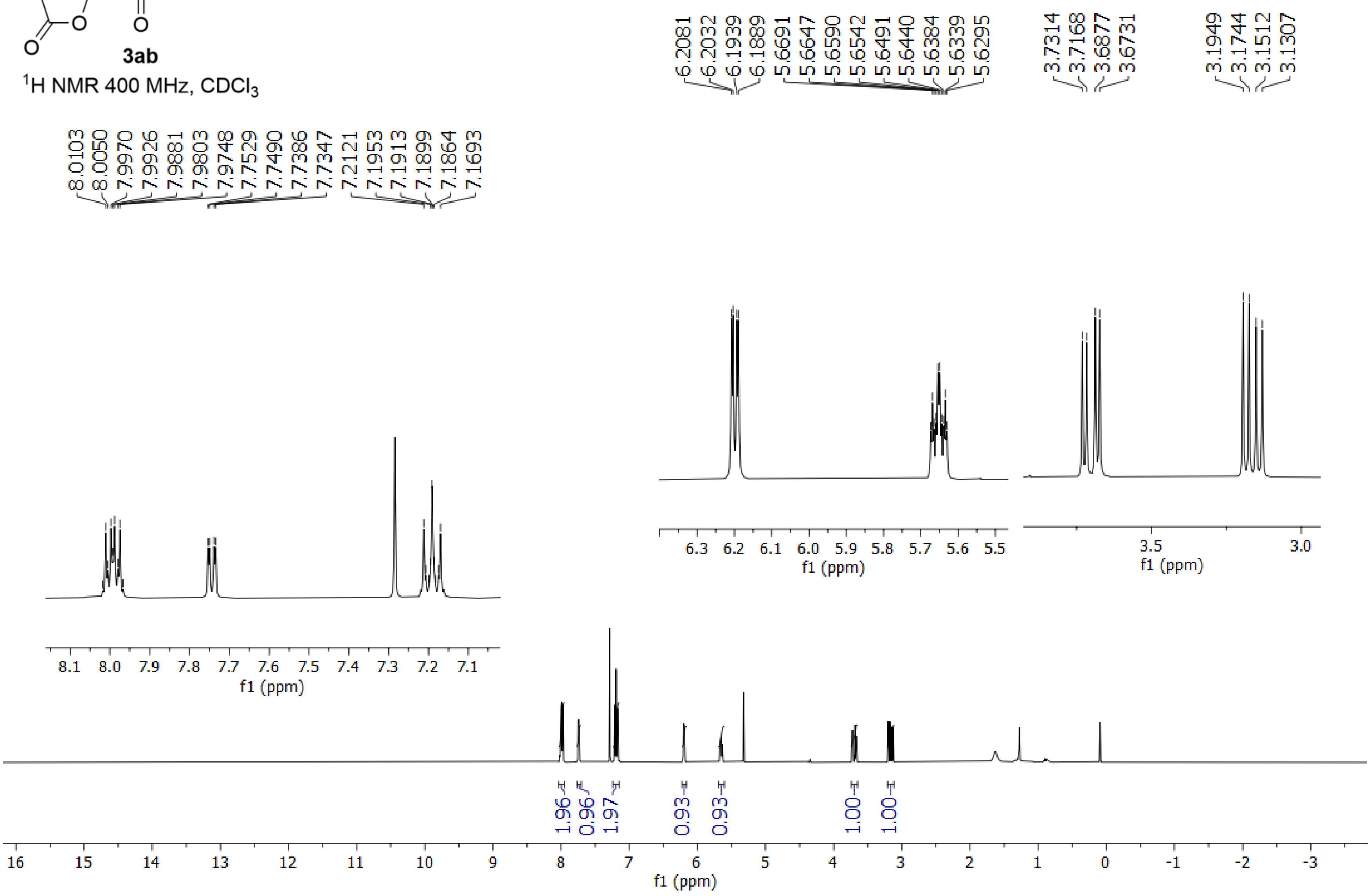

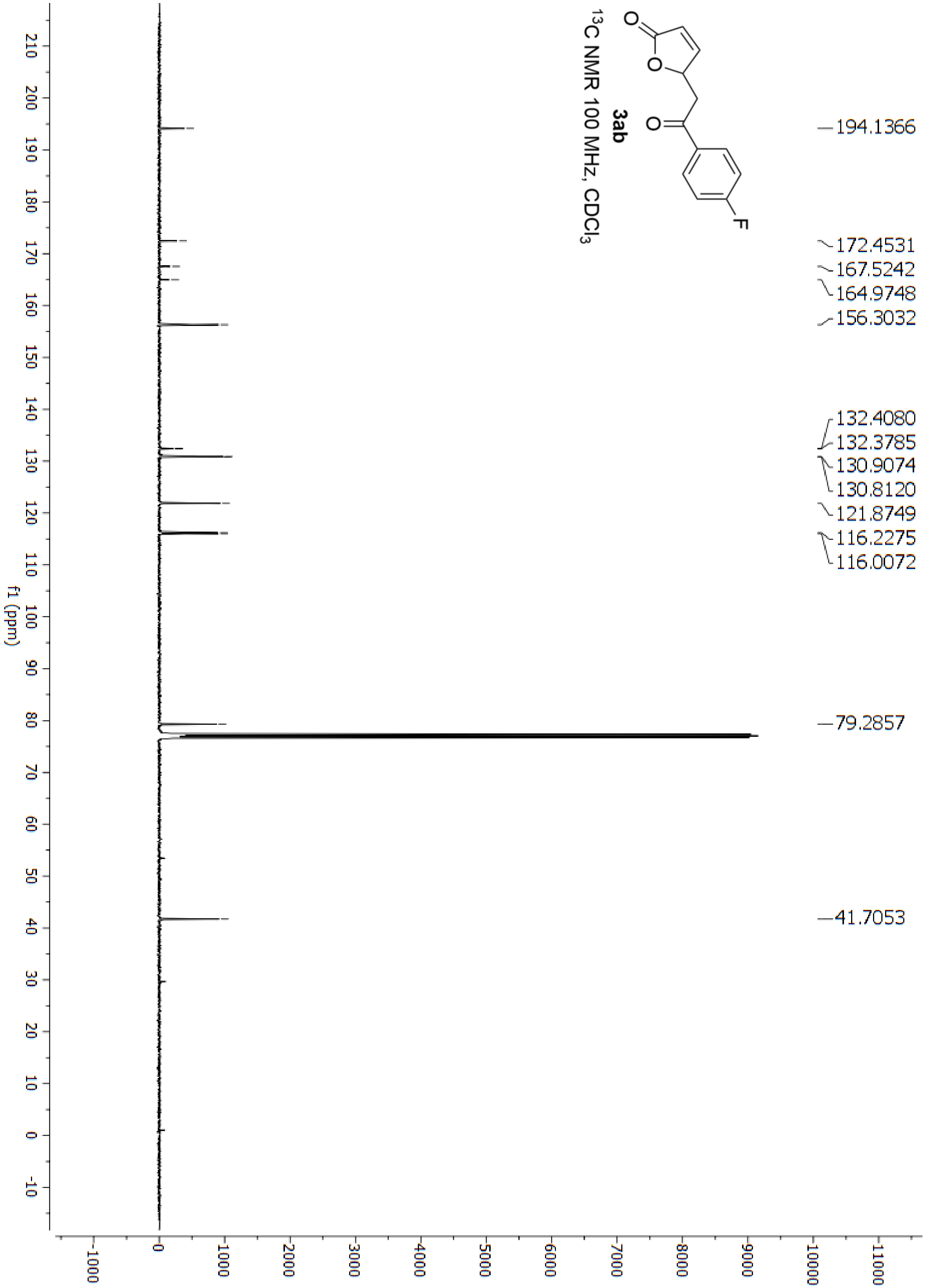
3ab
$^{13}$C NMR 100 MHz, CDCl$_3$
194.1366
172.4531
167.5242
164.9748
156.3032
132.4080
132.3785
130.9074
130.8120
121.8749
116.2275
116.0072
79.2857
41.7053
f1 (ppm)

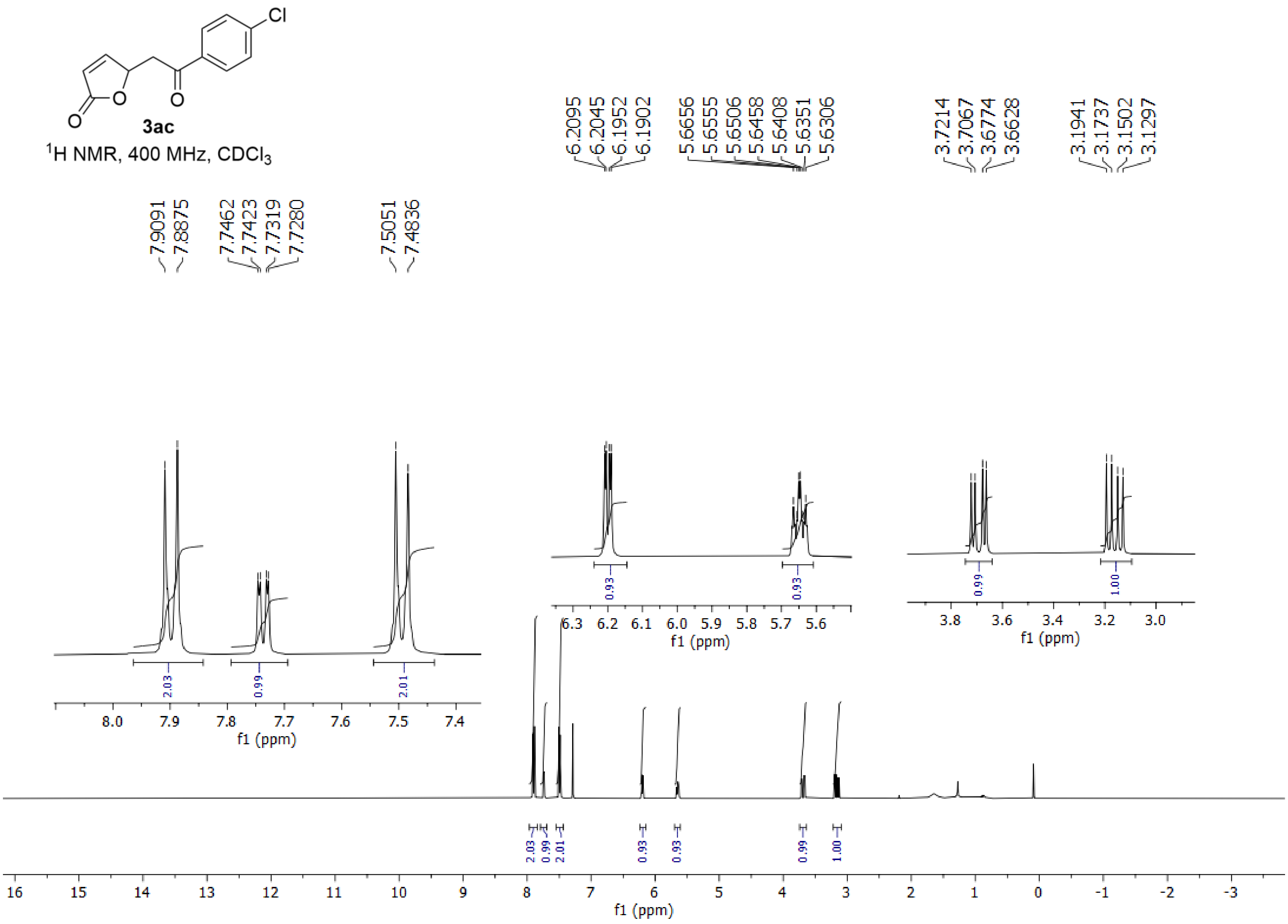
Cl
3ac
1H NMR, 400 MHz, CDCl3
7.9091
7.8875
7.7462
7.7423
7.7319
7.7280
7.5051
7.4836
6.2095
6.2045
6.1952
6.1902
5.6656
5.6555
5.6506
5.6458
5.6408
5.6351
5.6306
3.7214
3.7067
3.6774
3.6628
3.1941
3.1737
3.1502
3.1297
f1 (ppm)

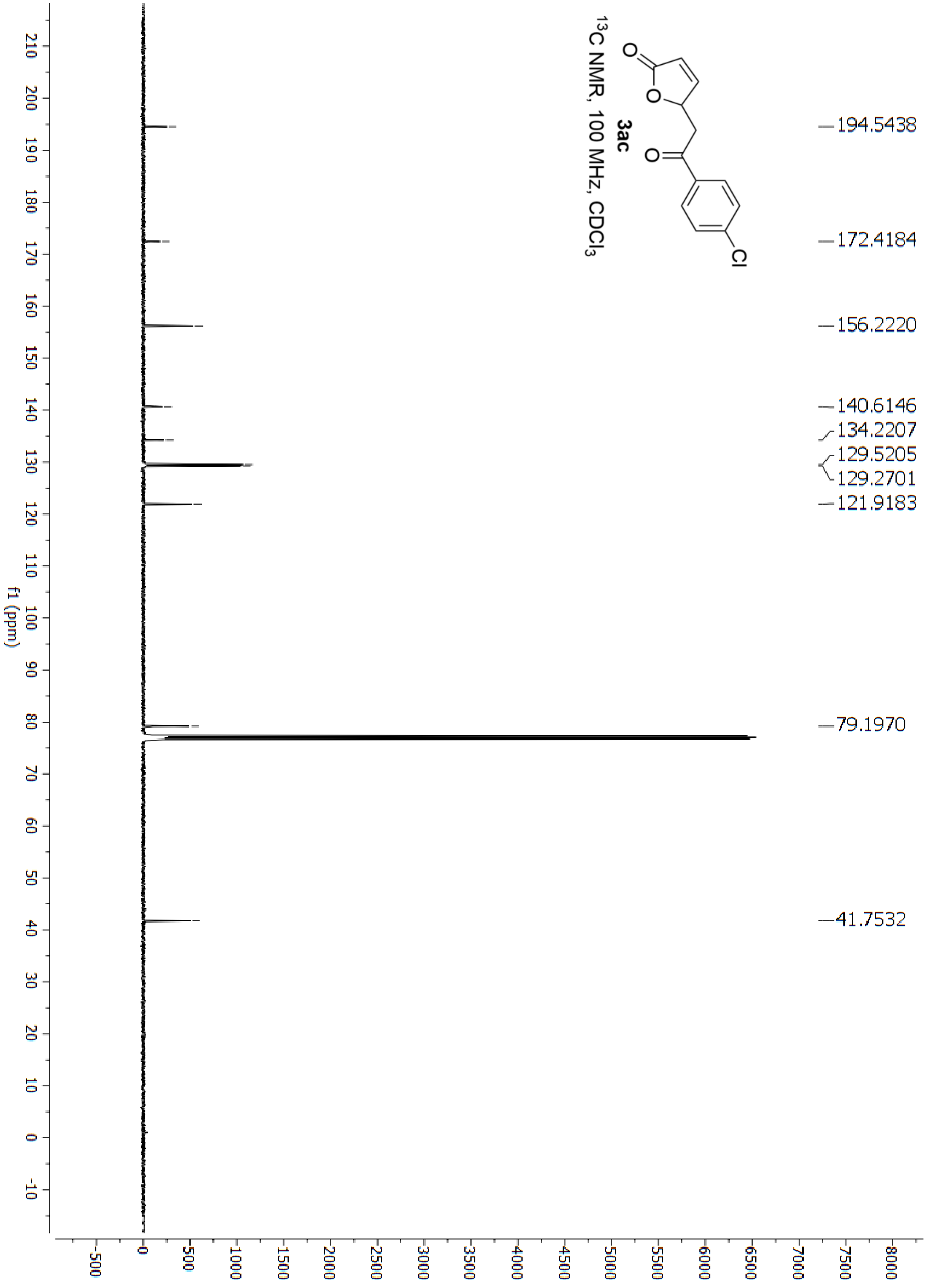

3ac
13C NMR, 100 MHz, CDCl3
194.5438
172.4184
156.2220
140.6146
134.2207
129.5205
129.2701
121.9183
79.1970
41.7532
f1 (ppm)

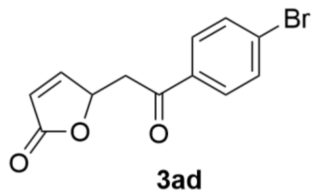

**3ad**

$^1$H NMR 400 MHz, $CDCl_3$

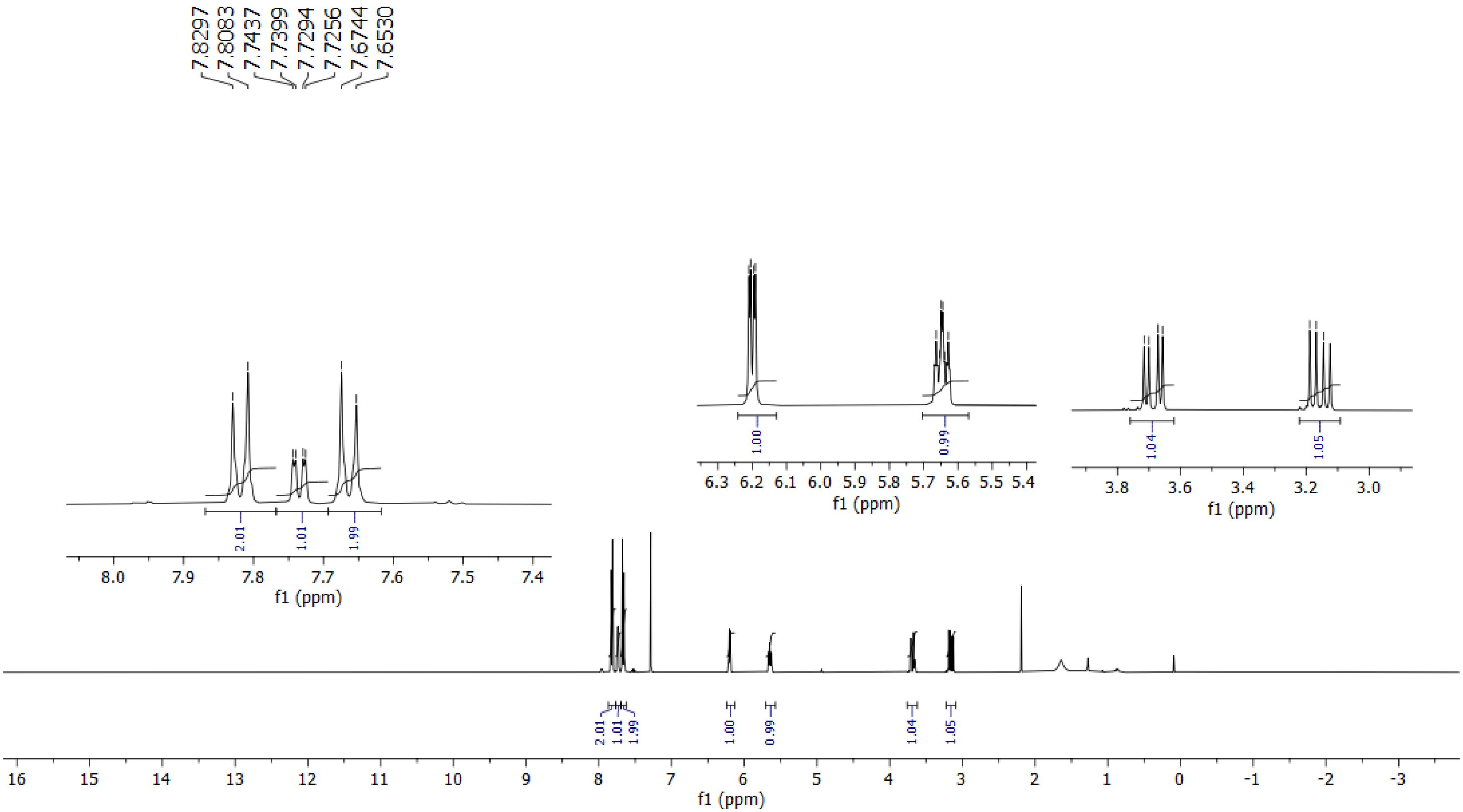

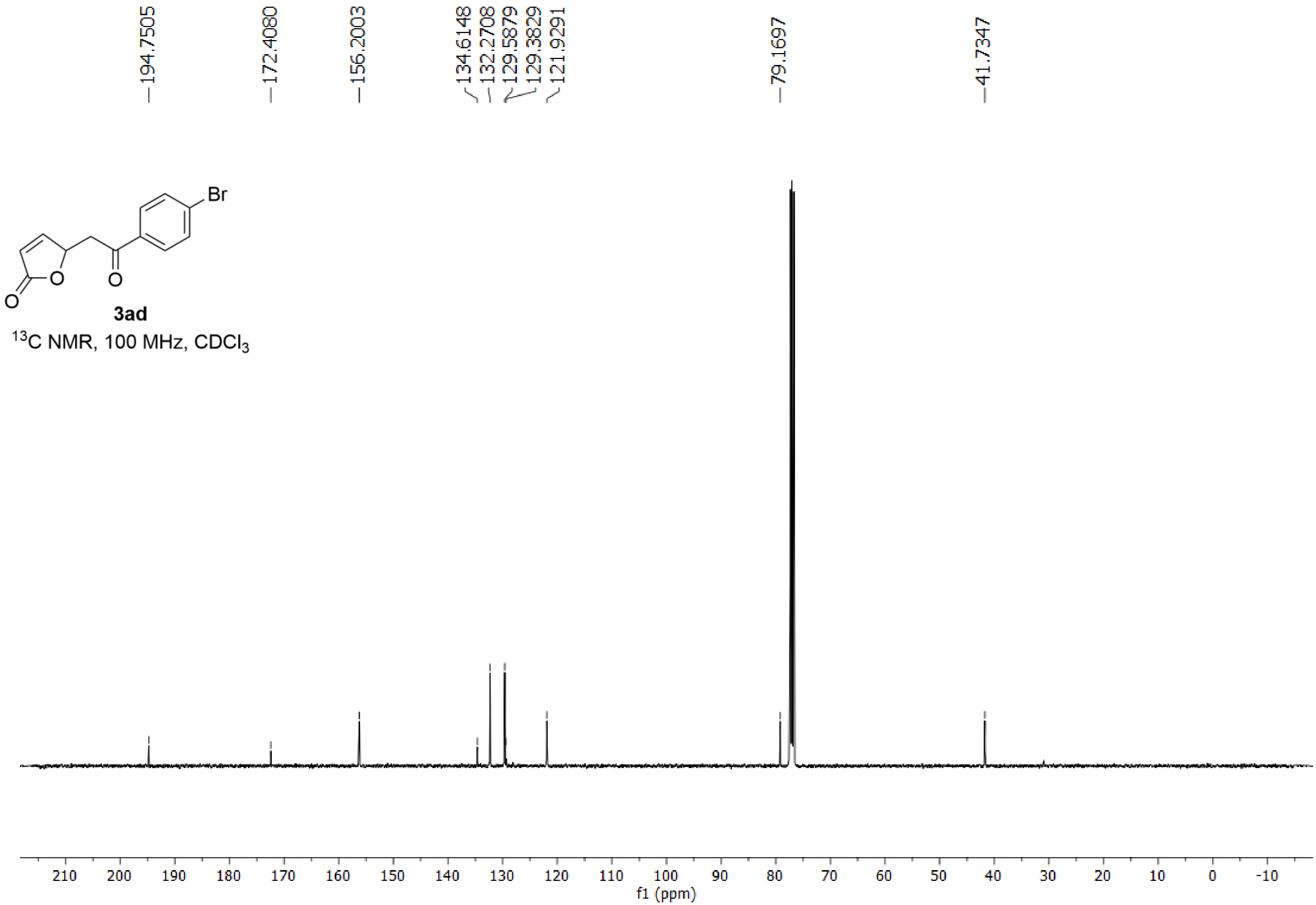
194.7505
172.4080
156.2003
134.6148
132.2708
129.5879
129.3829
121.9291
79.1697
41.7347
Br
O
3ad
$^{13}$C NMR, 100 MHz, $CDCl_3$
210 200 190 180 170 160 150 140 130 120 110 100 90 80 70 60 50 40 30 20 10 0 -10
f1 (ppm)

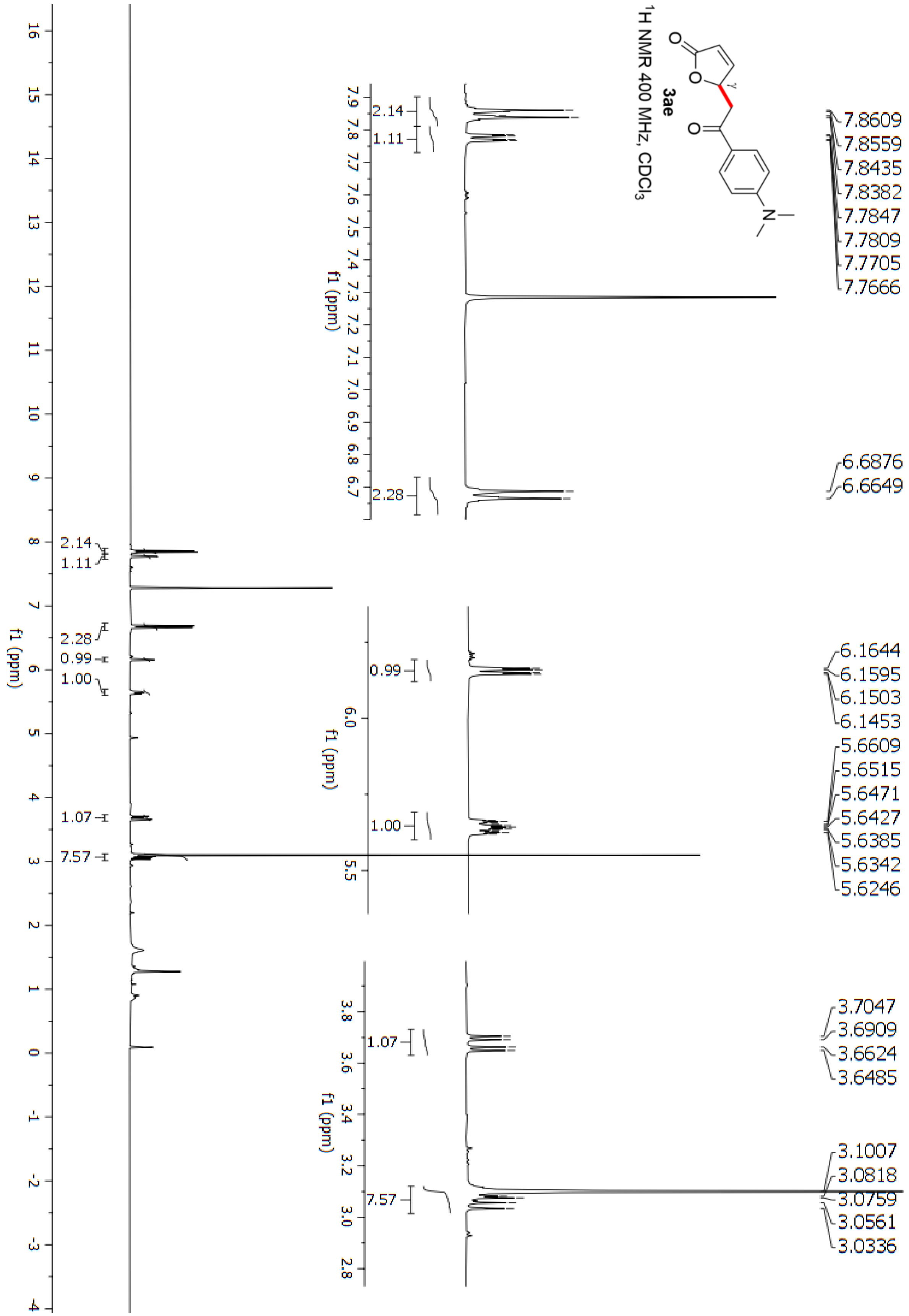
3ae
$^1$H NMR 400 MHz, CDCl$_3$
7.8609
7.8559
7.8435
7.8382
7.7847
7.7809
7.7705
7.7666
6.6876
6.6649
6.1644
6.1595
6.1503
6.1453
5.6609
5.6515
5.6471
5.6427
5.6385
5.6342
5.6246
3.7047
3.6909
3.6624
3.6485
3.1007
3.0818
3.0759
3.0561
3.0336
2.14
1.11
2.28
0.99
1.00
1.07
7.57
f1 (ppm)

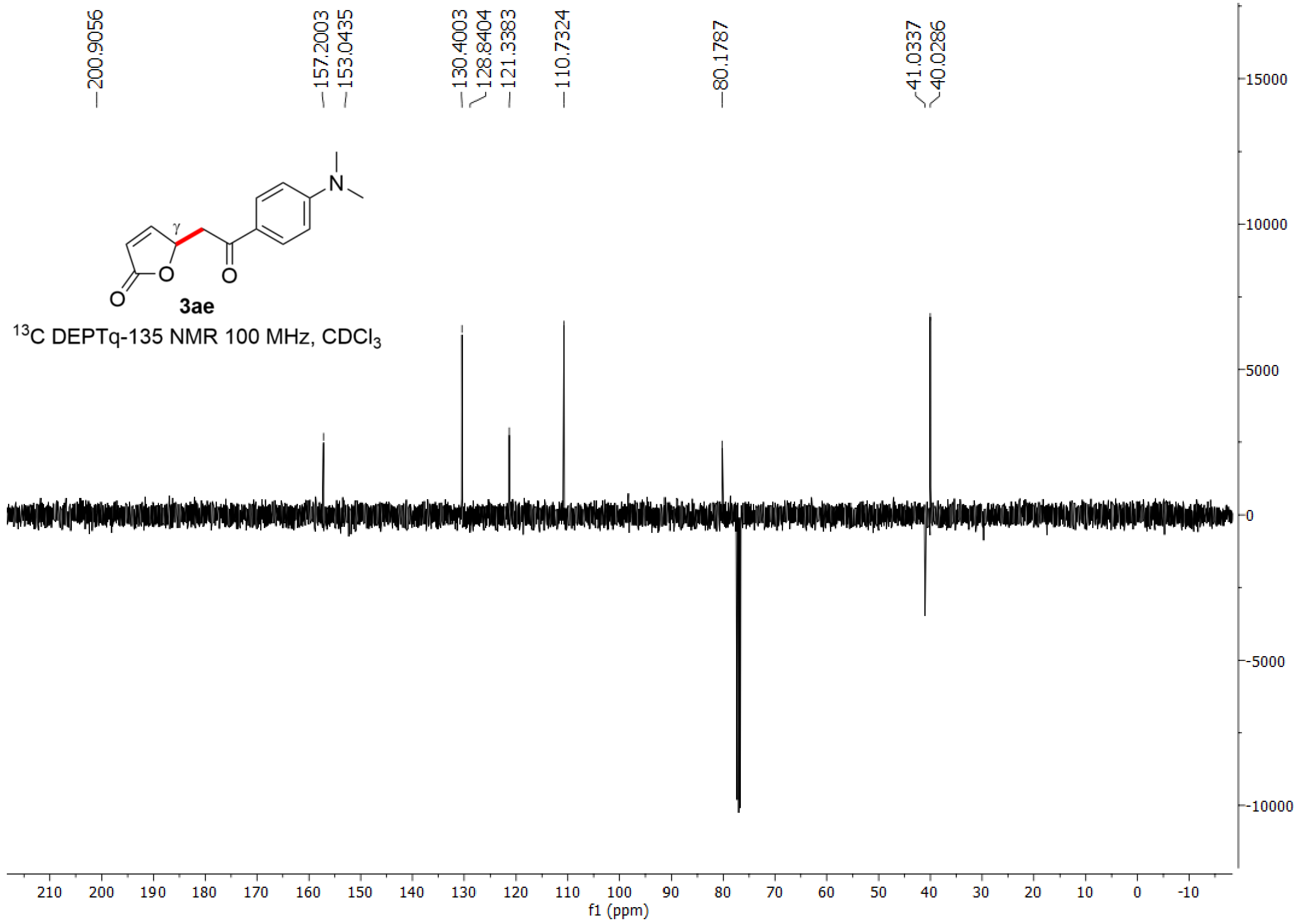
200.9056
157.2003
153.0435
130.4003
128.8404
121.3383
110.7324
80.1787
41.0337
40.0286
3ae
13C DEPTq-135 NMR 100 MHz, CDCl3
f1 (ppm)

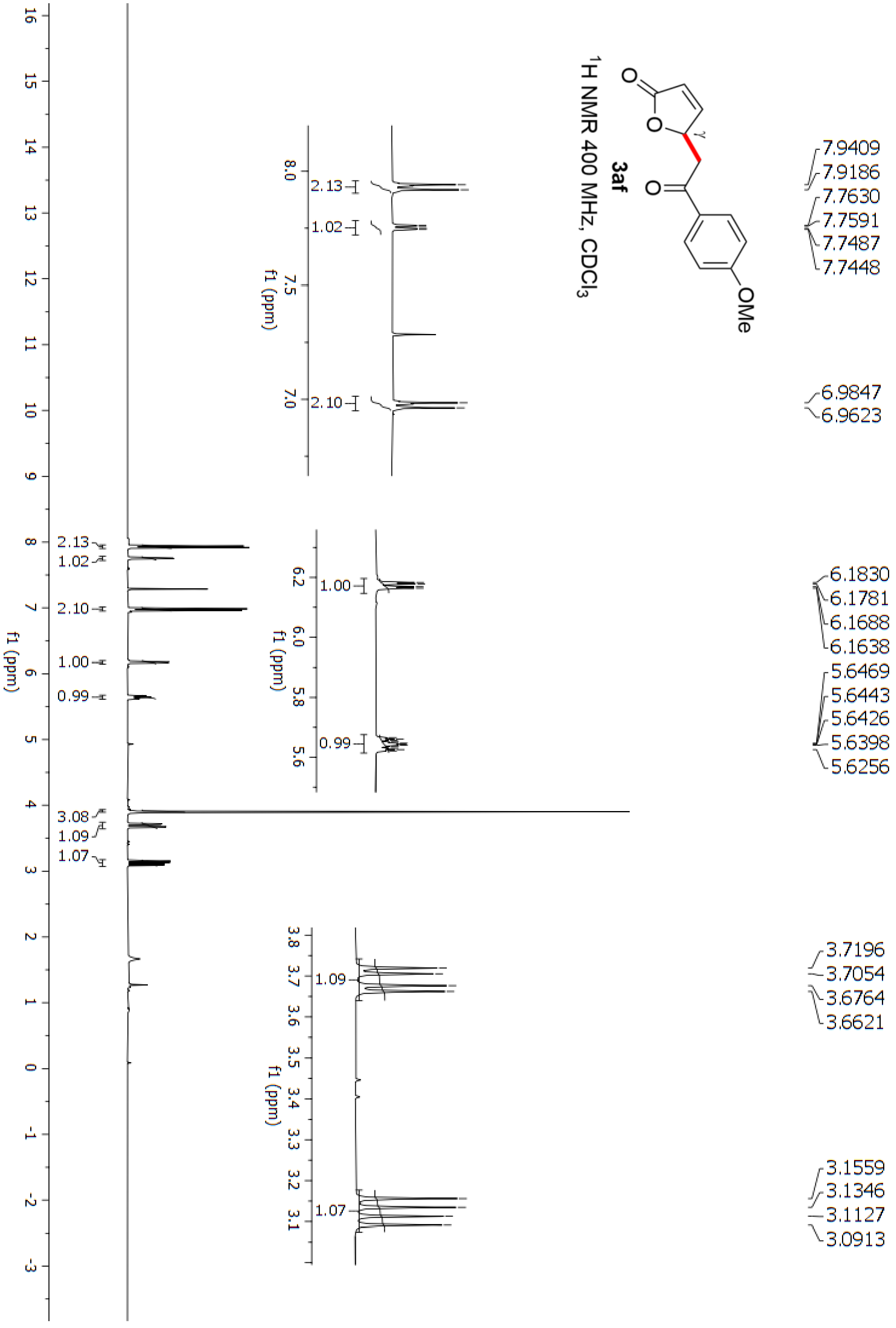

OMe
3af
1H NMR 400 MHz, CDCl3
7.9409
7.9186
7.7630
7.7591
7.7487
7.7448
6.9847
6.9623
6.1830
6.1781
6.1688
6.1638
5.6469
5.6443
5.6426
5.6398
5.6256
3.7196
3.7054
3.6764
3.6621
3.1559
3.1346
3.1127
3.0913
f1 (ppm)

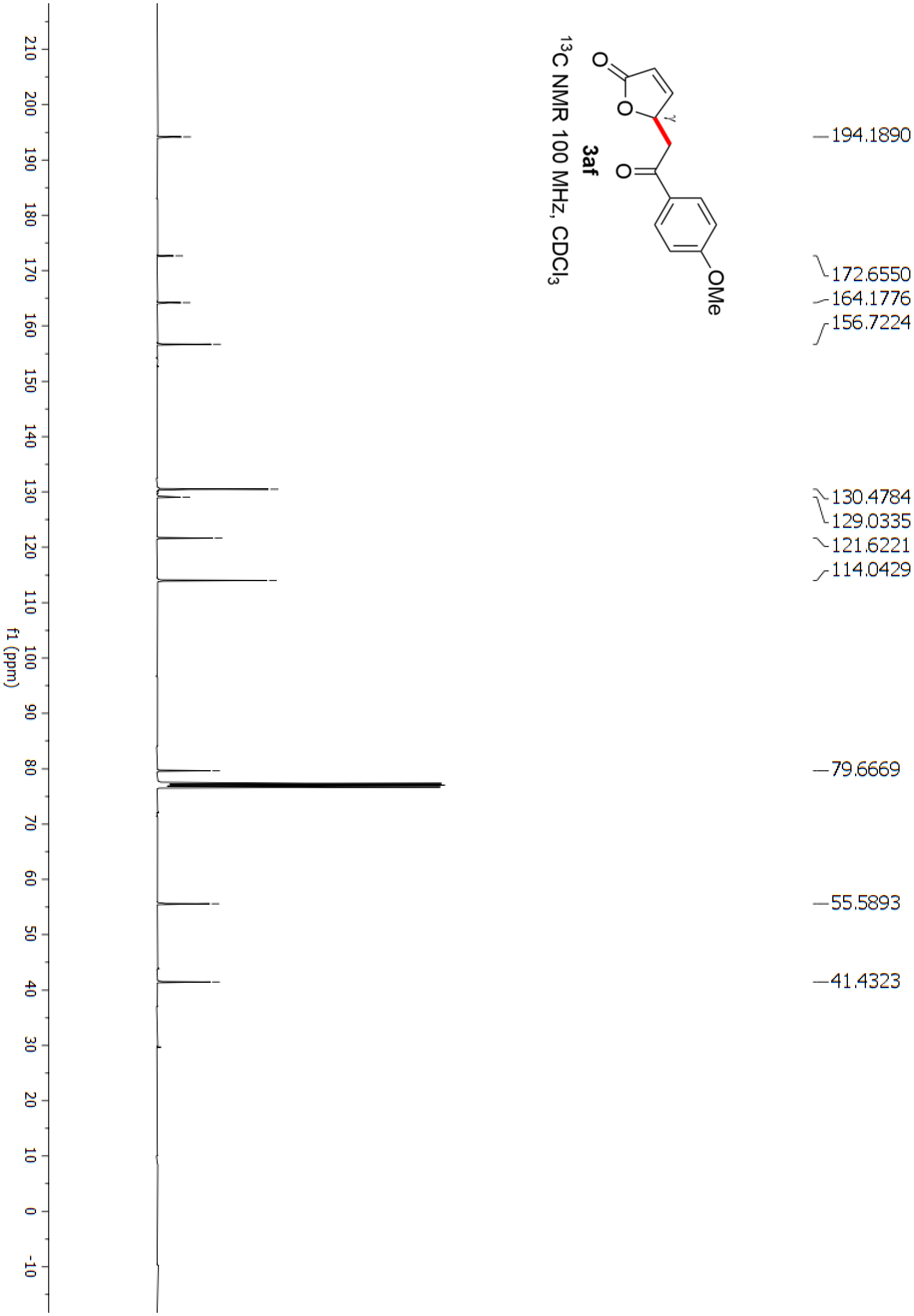
194.1890
172.6550
164.1776
156.7224
130.4784
129.0335
121.6221
114.0429
79.6669
55.5893
41.4323
OMe
3af
13C NMR 100 MHz, CDCl3
f1 (ppm)

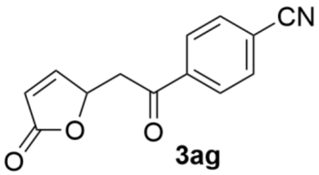

**3ag**

$^{1}$H NMR 400 MHz, $CDCl_3$

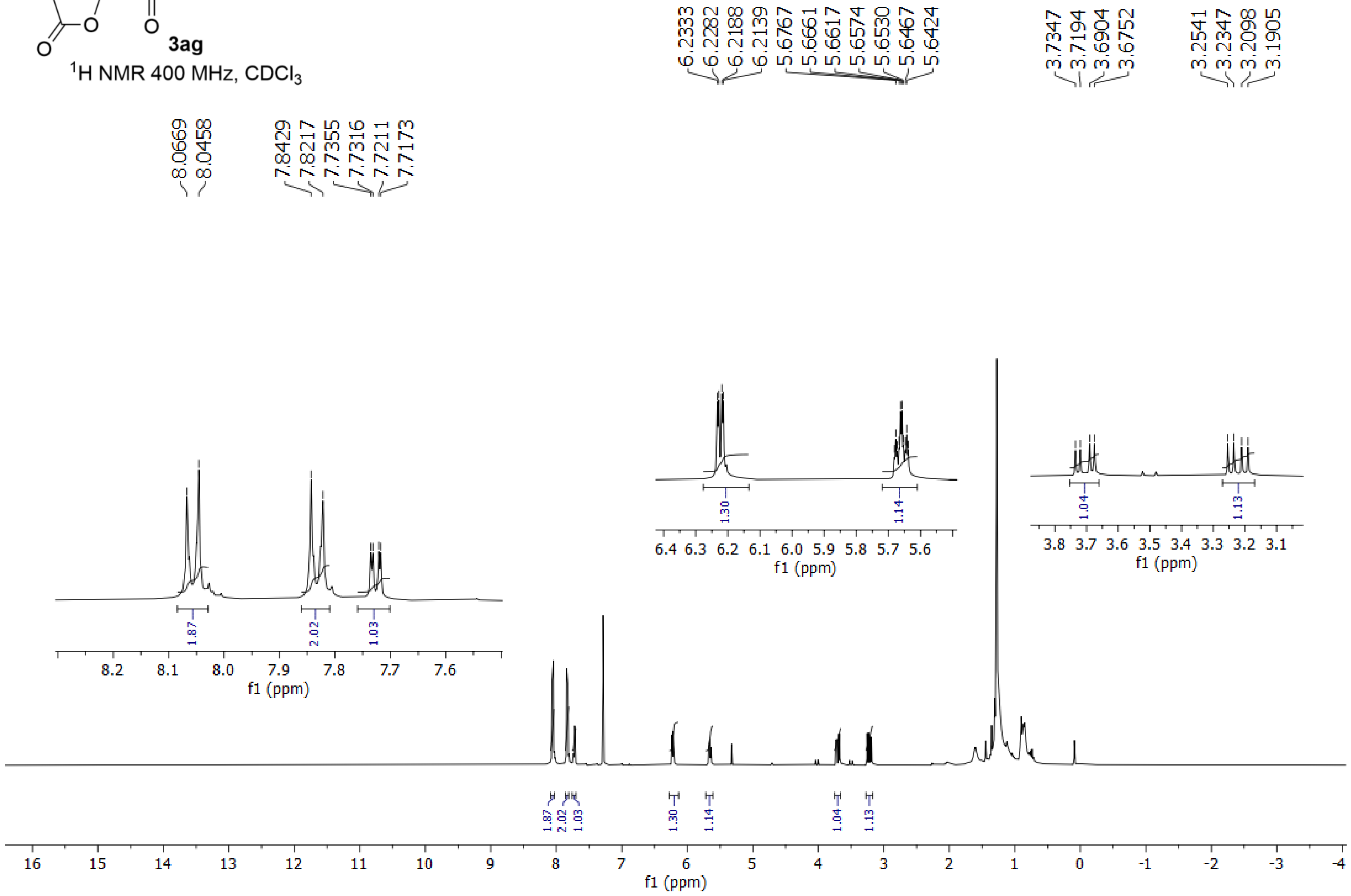

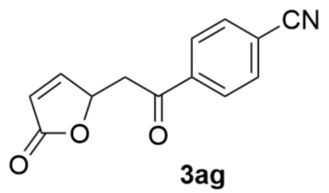

**3ag**

$^{13}$C-DEPTq-135 NMR, 100 MHz, $CDCl_3$

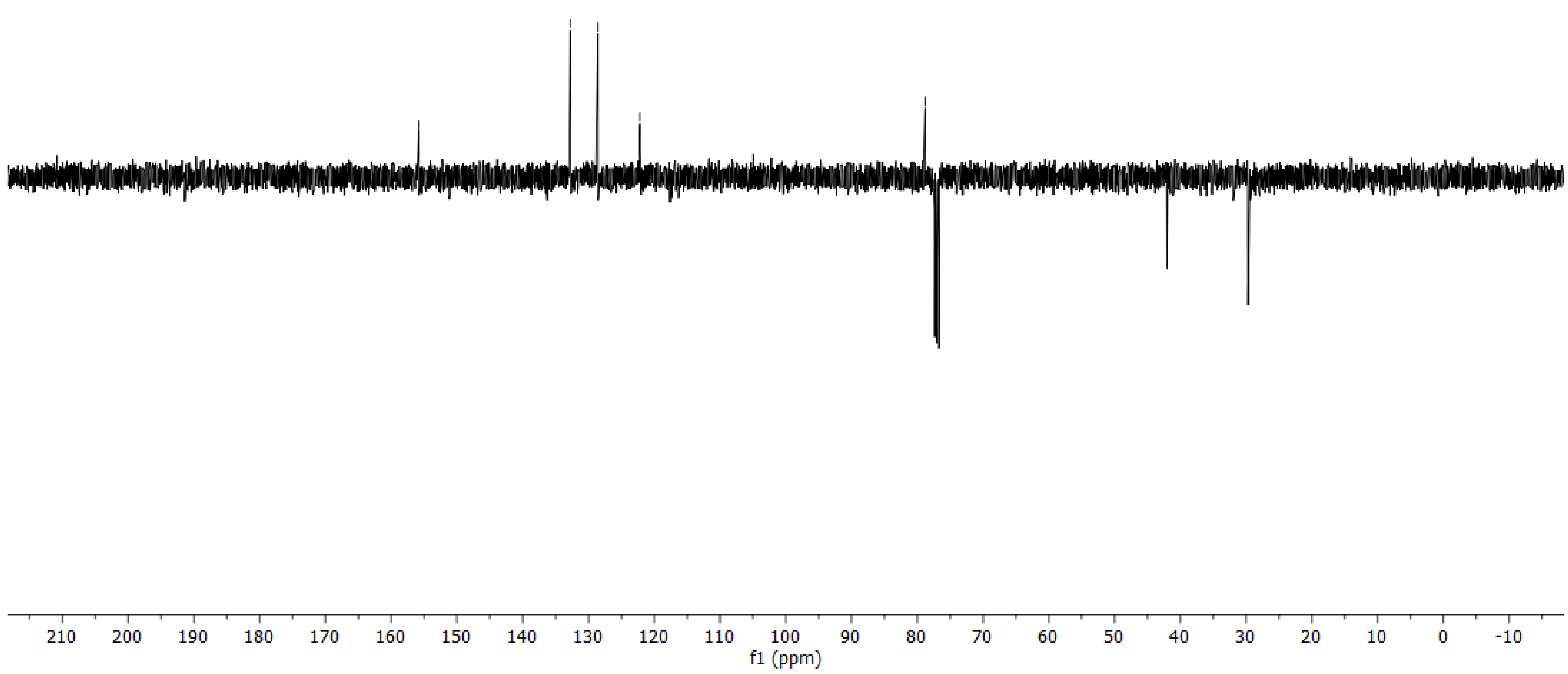

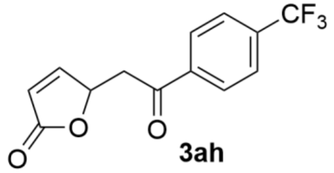

**3ah**

$^{1}H$ NMR 400 MHz, $CDCl_3$

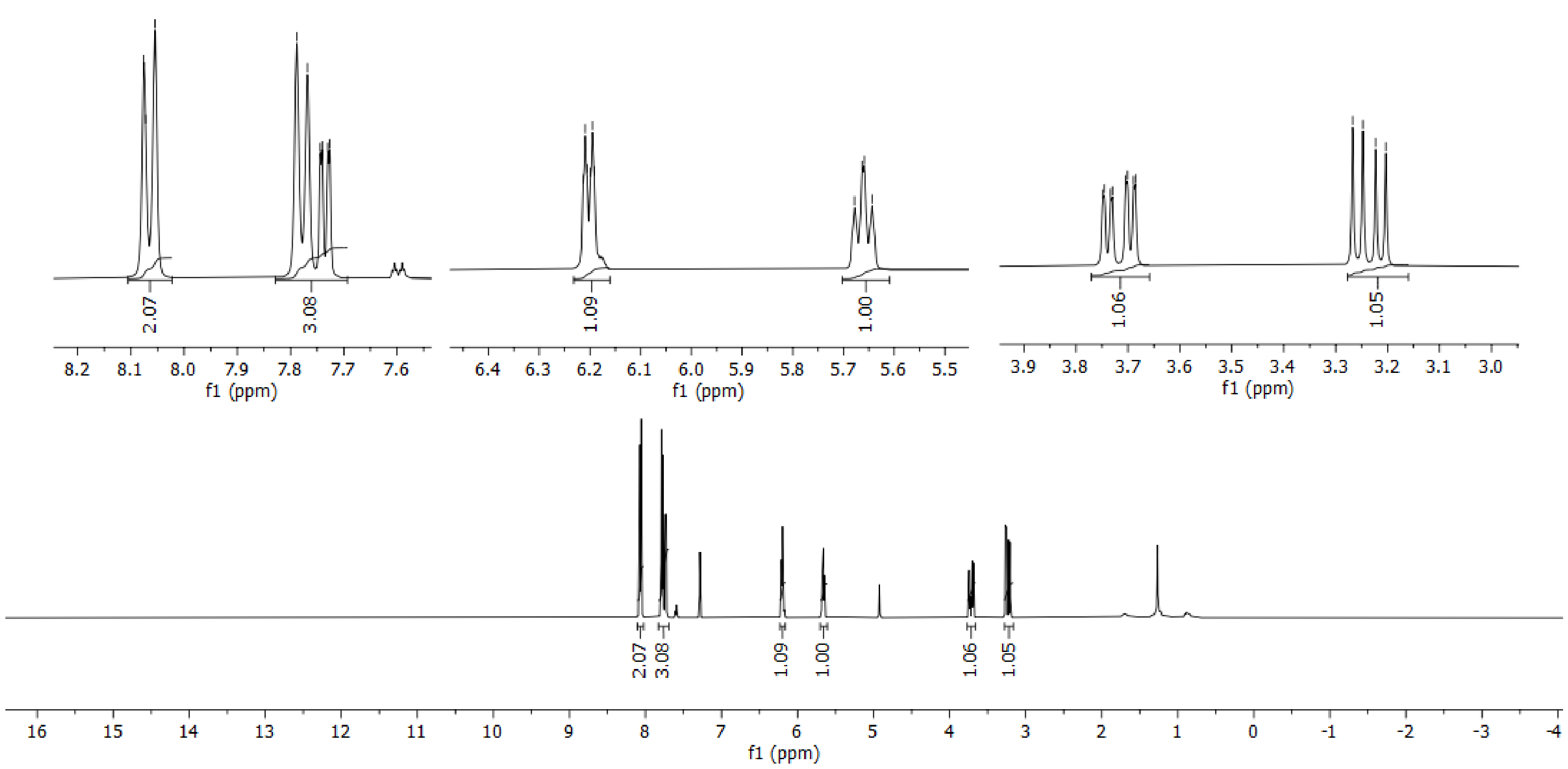

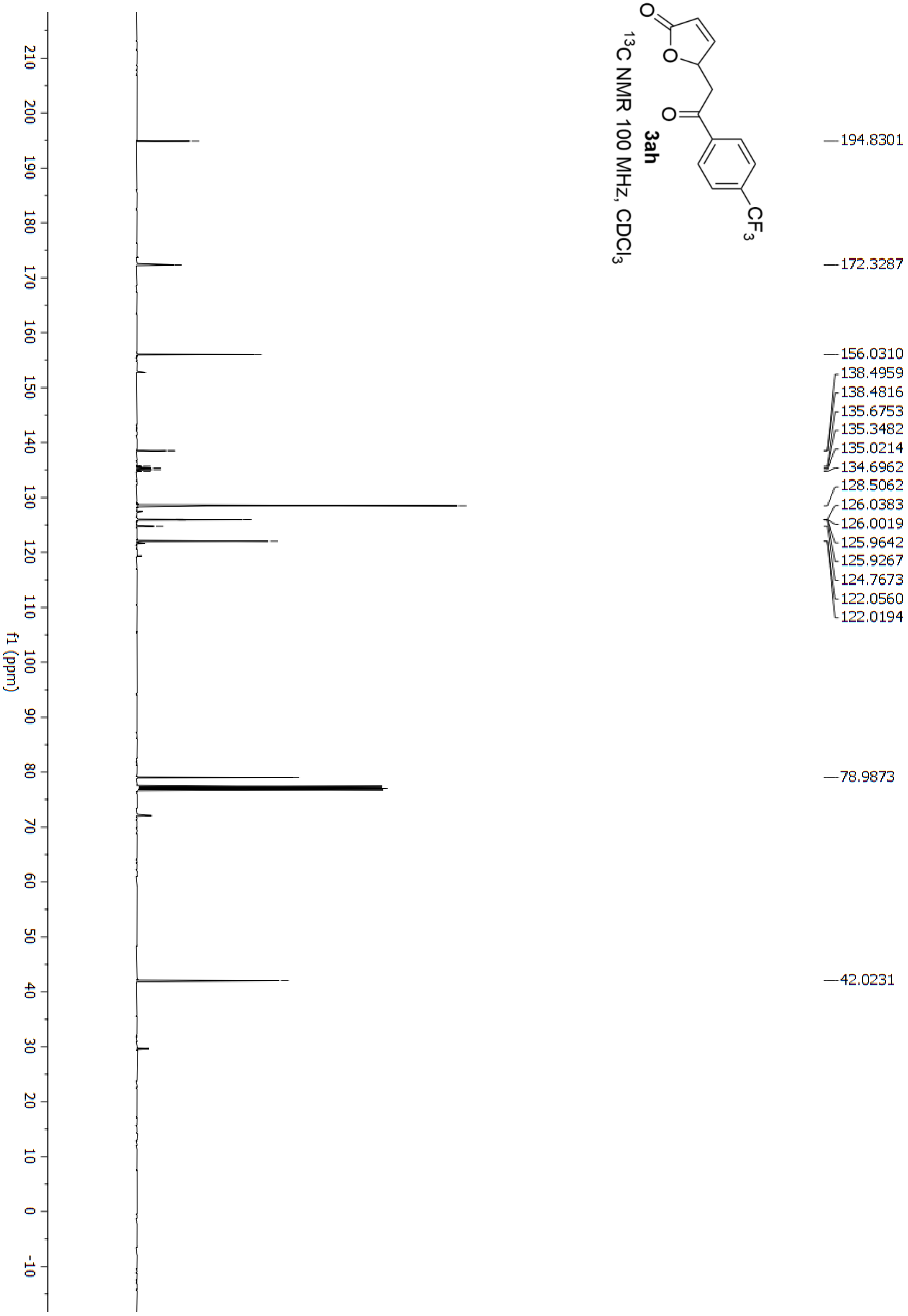
3ah
$^{13}C$ NMR 100 MHz, $CDCl_3$
194.8301
172.3287
156.0310
138.4959
138.4816
135.6753
135.3482
135.0214
134.6962
128.5062
126.0383
126.0019
125.9642
125.9267
124.7673
122.0560
122.0194
78.9873
42.0231
f1 (ppm)

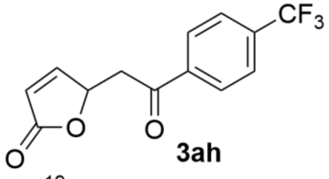

**3ah**

$^{19}F$ NMR 564 MHz, $CDCl_3$

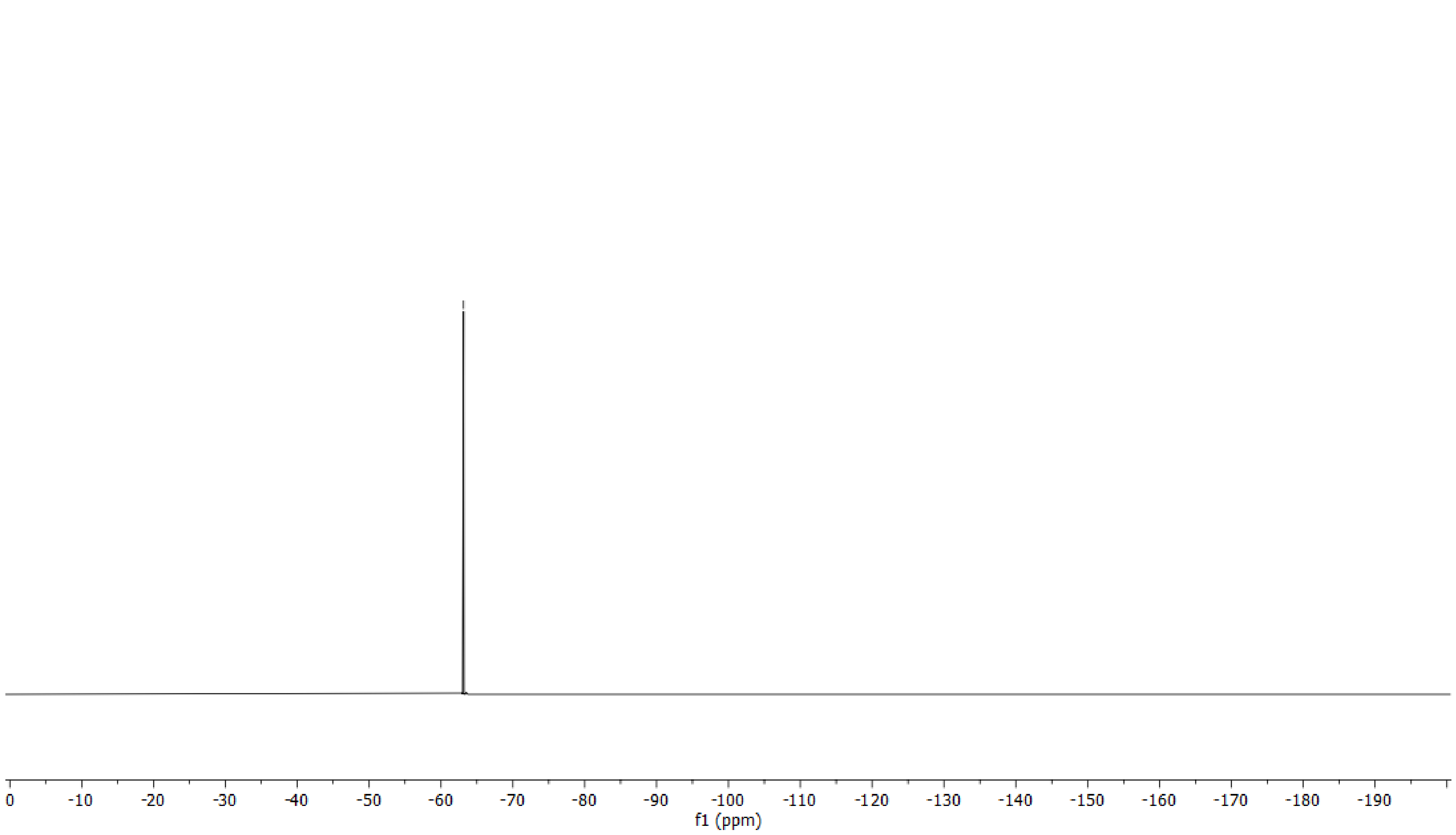

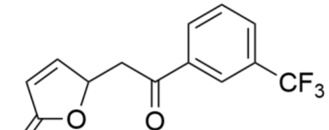

**3ai**

$^1H$ NMR 400 MHz, $CDCl_3$

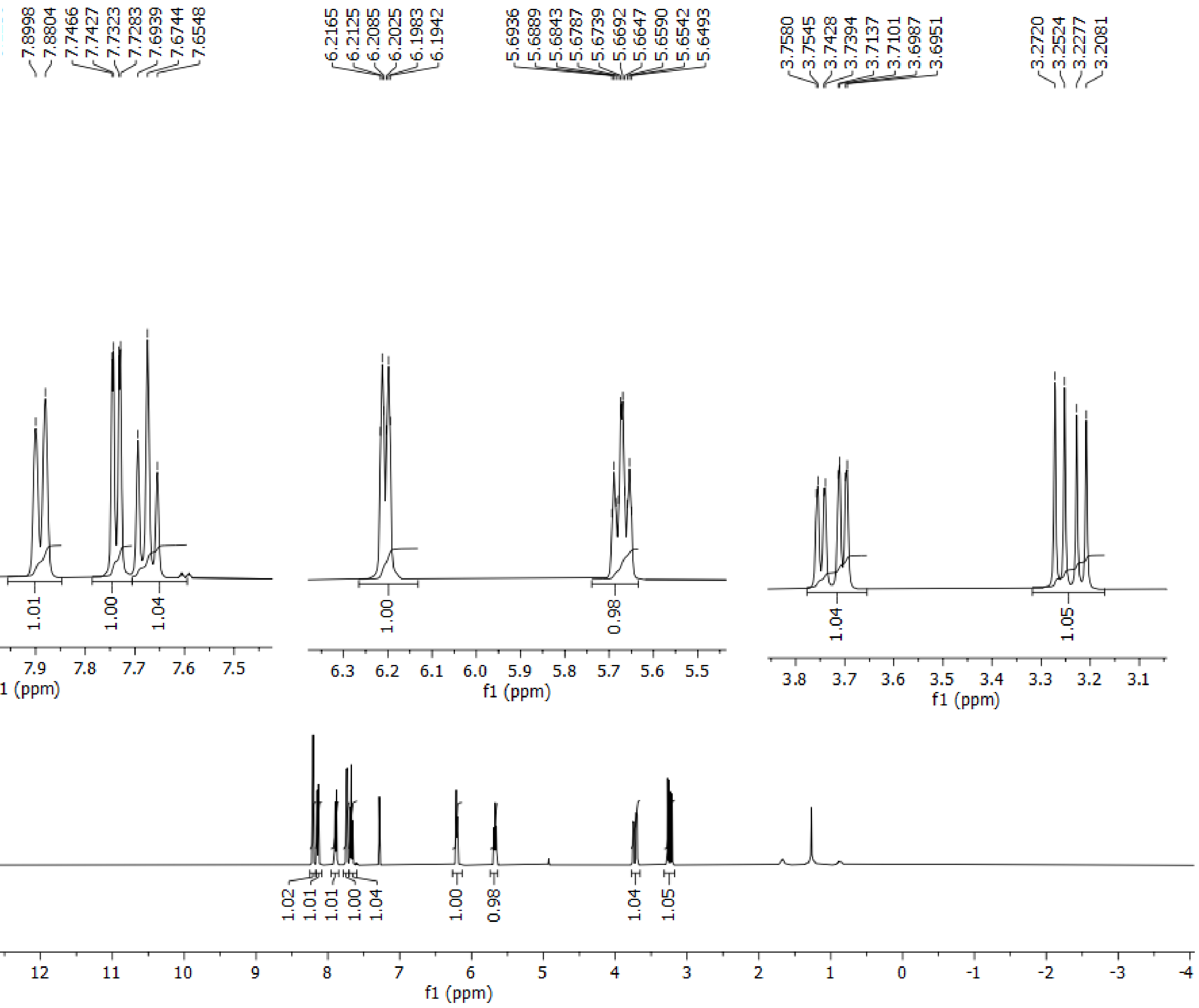

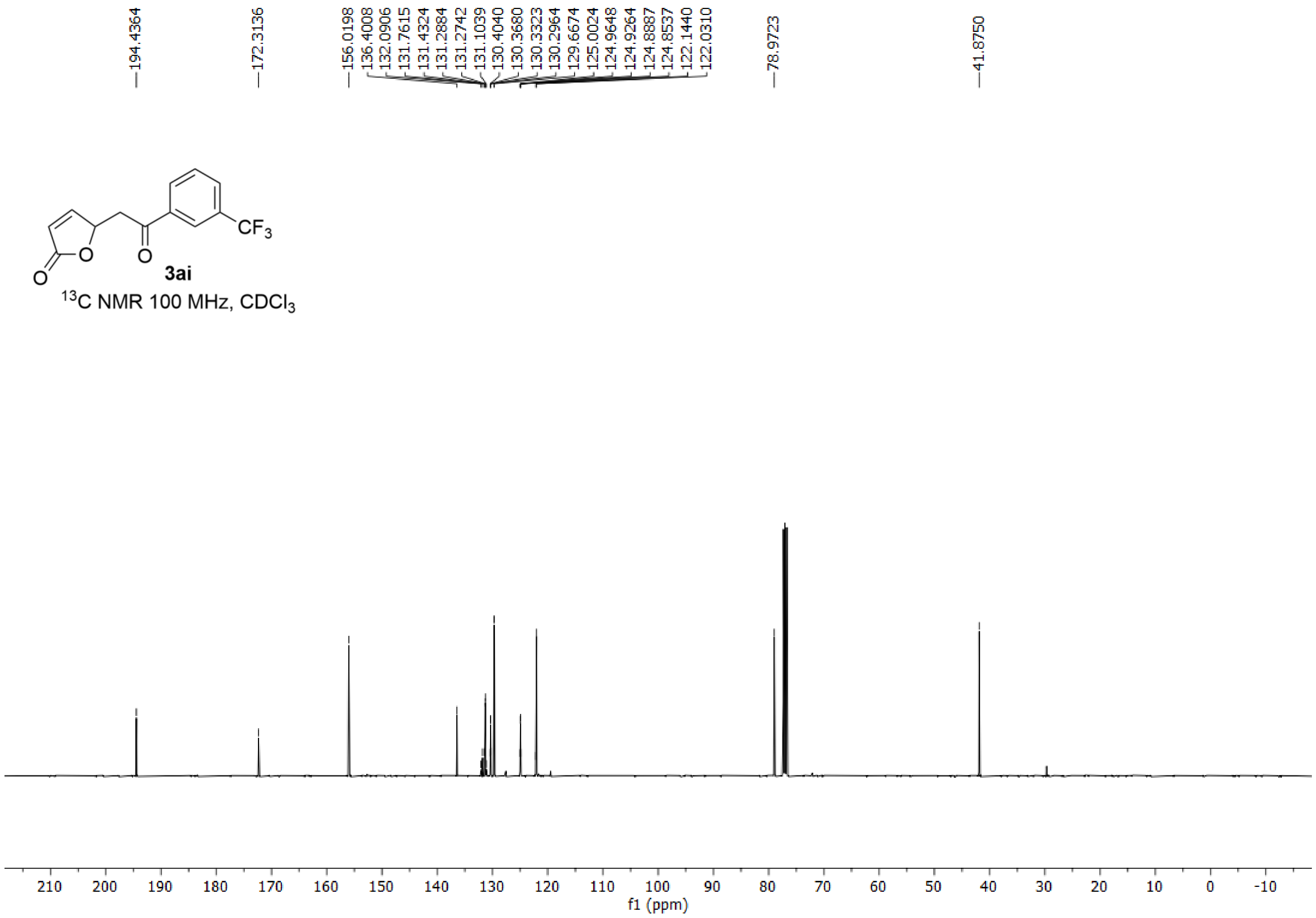
194.4364
172.3136
156.0198
136.4008
132.0906
131.7615
131.4324
131.2884
131.2742
131.1039
130.4040
130.3680
130.3323
130.2964
129.6674
125.0024
124.9648
124.9264
124.8887
124.8537
122.1440
122.0310
78.9723
41.8750
$CF_3$
O
O
O
3ai
$^{13}C$ NMR 100 MHz, $CDCl_3$
210 200 190 180 170 160 150 140 130 120 110 100 90 80 70 60 50 40 30 20 10 0 -10
f1 (ppm)

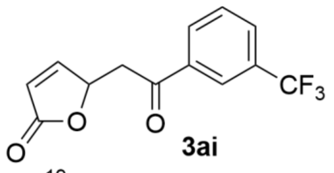


**3ai**

$^{19}F$ NMR 564 MHz, $CDCl_3$

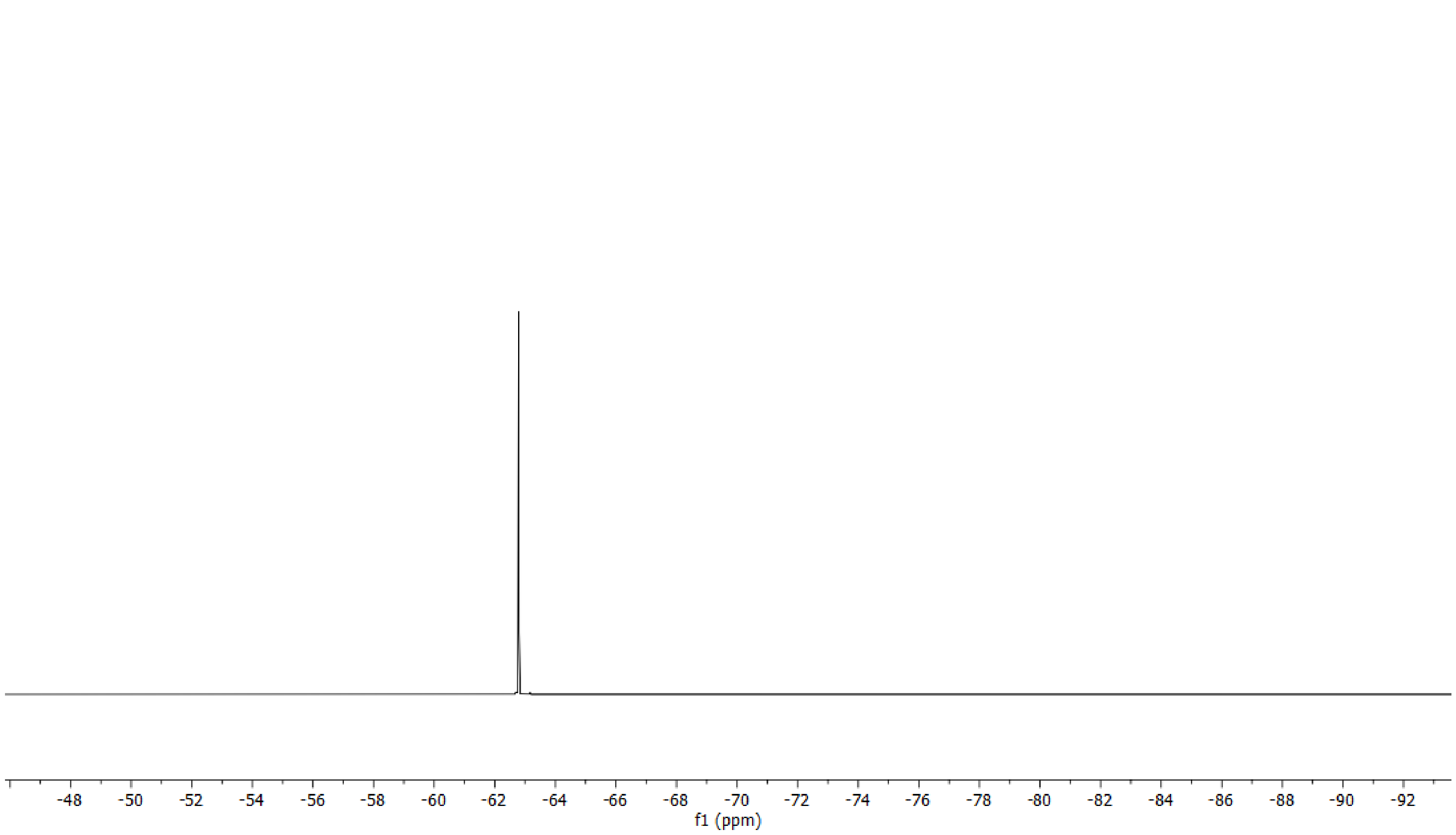

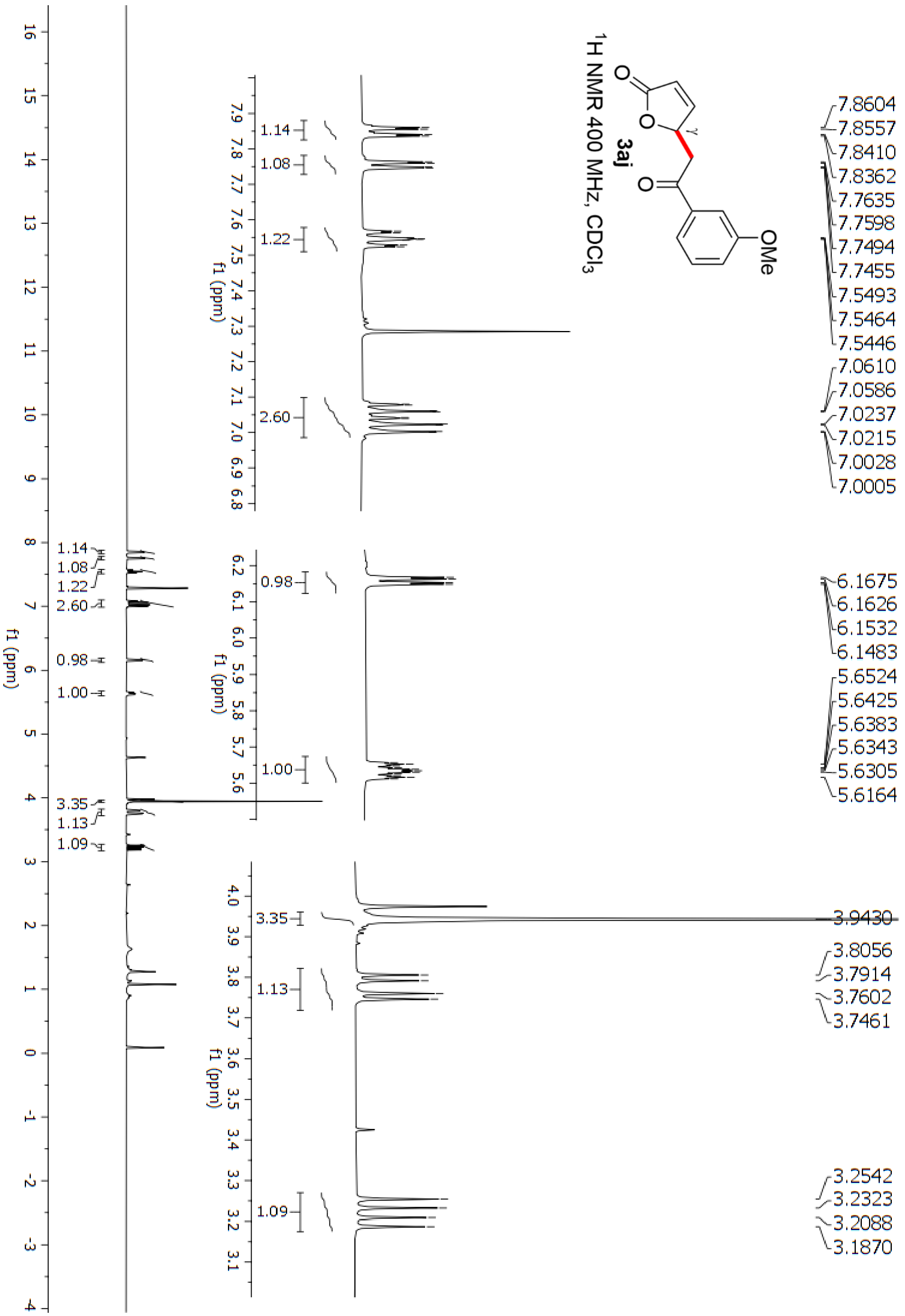

OMe
3aj
1H NMR 400 MHz, CDCl3
7.8604
7.8557
7.8410
7.8362
7.7635
7.7598
7.7494
7.7455
7.5493
7.5464
7.5446
7.0610
7.0586
7.0237
7.0215
7.0028
7.0005
6.1675
6.1626
6.1532
6.1483
5.6524
5.6425
5.6383
5.6343
5.6305
5.6164
3.9430
3.8056
3.7914
3.7602
3.7461
3.2542
3.2323
3.2088
3.1870
f1 (ppm)

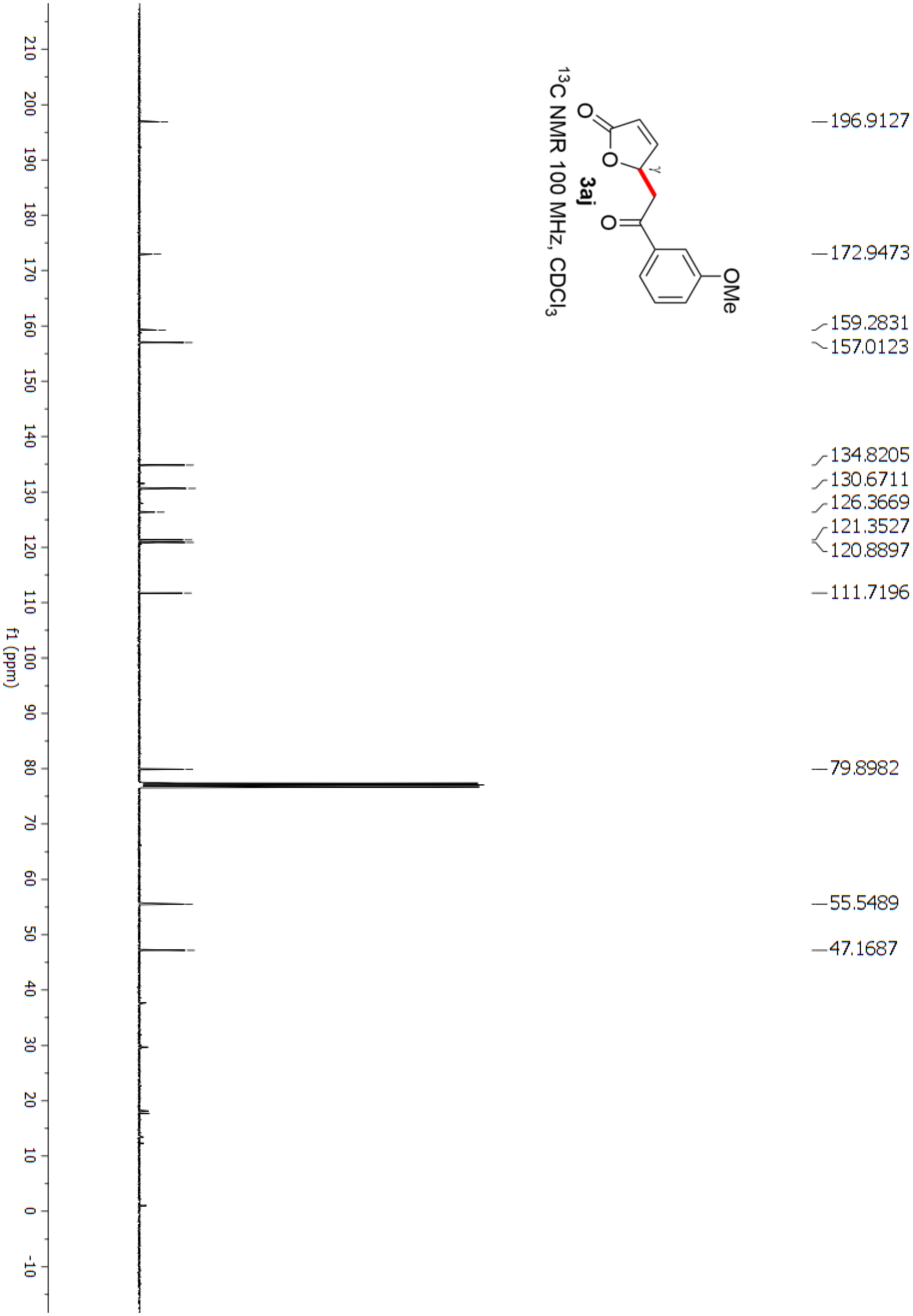

3aj
13C NMR 100 MHz, CDCl3
OMe
196.9127
172.9473
159.2831
157.0123
134.8205
130.6711
126.3669
121.3527
120.8897
111.7196
79.8982
55.5489
47.1687
f1 (ppm)

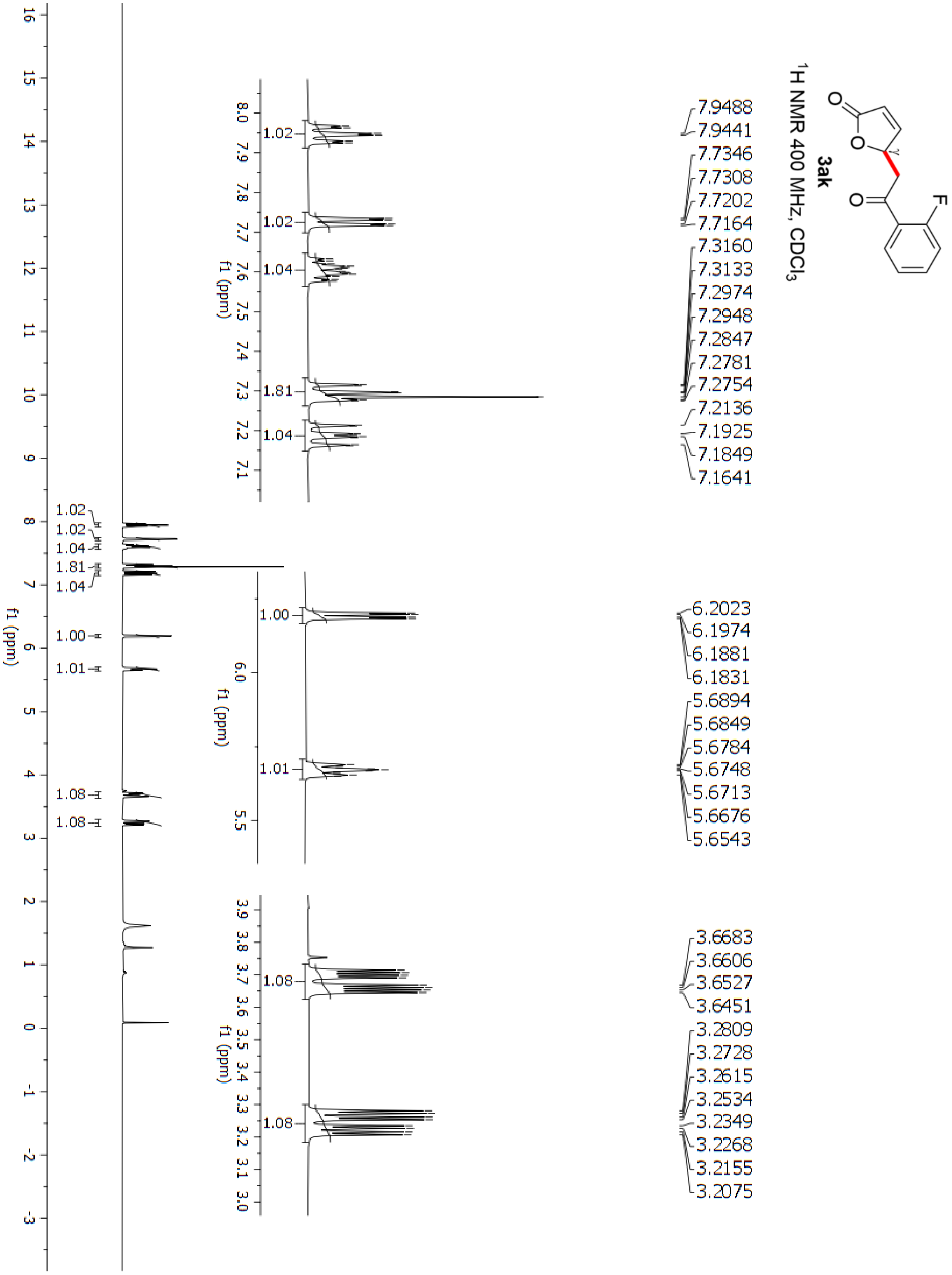

3ak
¹H NMR 400 MHz, CDCl3
7.9488
7.9441
7.7346
7.7308
7.7202
7.7164
7.3160
7.3133
7.2974
7.2948
7.2847
7.2781
7.2754
7.2136
7.1925
7.1849
7.1641
6.2023
6.1974
6.1881
6.1831
5.6894
5.6849
5.6784
5.6748
5.6713
5.6676
5.6543
3.6683
3.6606
3.6527
3.6451
3.2809
3.2728
3.2615
3.2534
3.2349
3.2268
3.2155
3.2075
f1 (ppm)

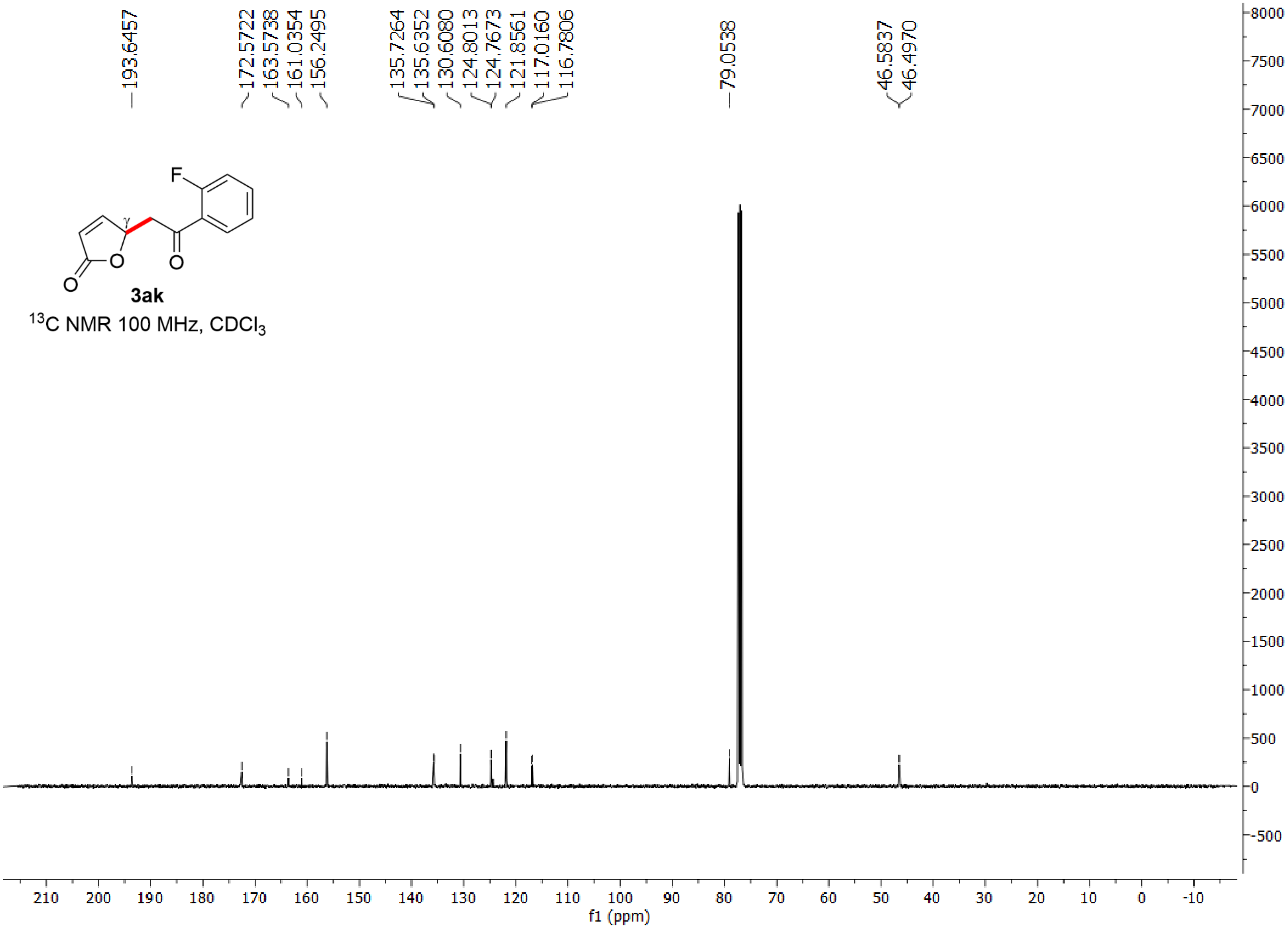
193.6457
172.5722
163.5738
161.0354
156.2495
135.7264
135.6352
130.6080
124.8013
124.7673
121.8561
117.0160
116.7806
79.0538
46.5837
46.4970
F
O
O
O
γ
3ak
13C NMR 100 MHz, CDCl3
f1 (ppm)

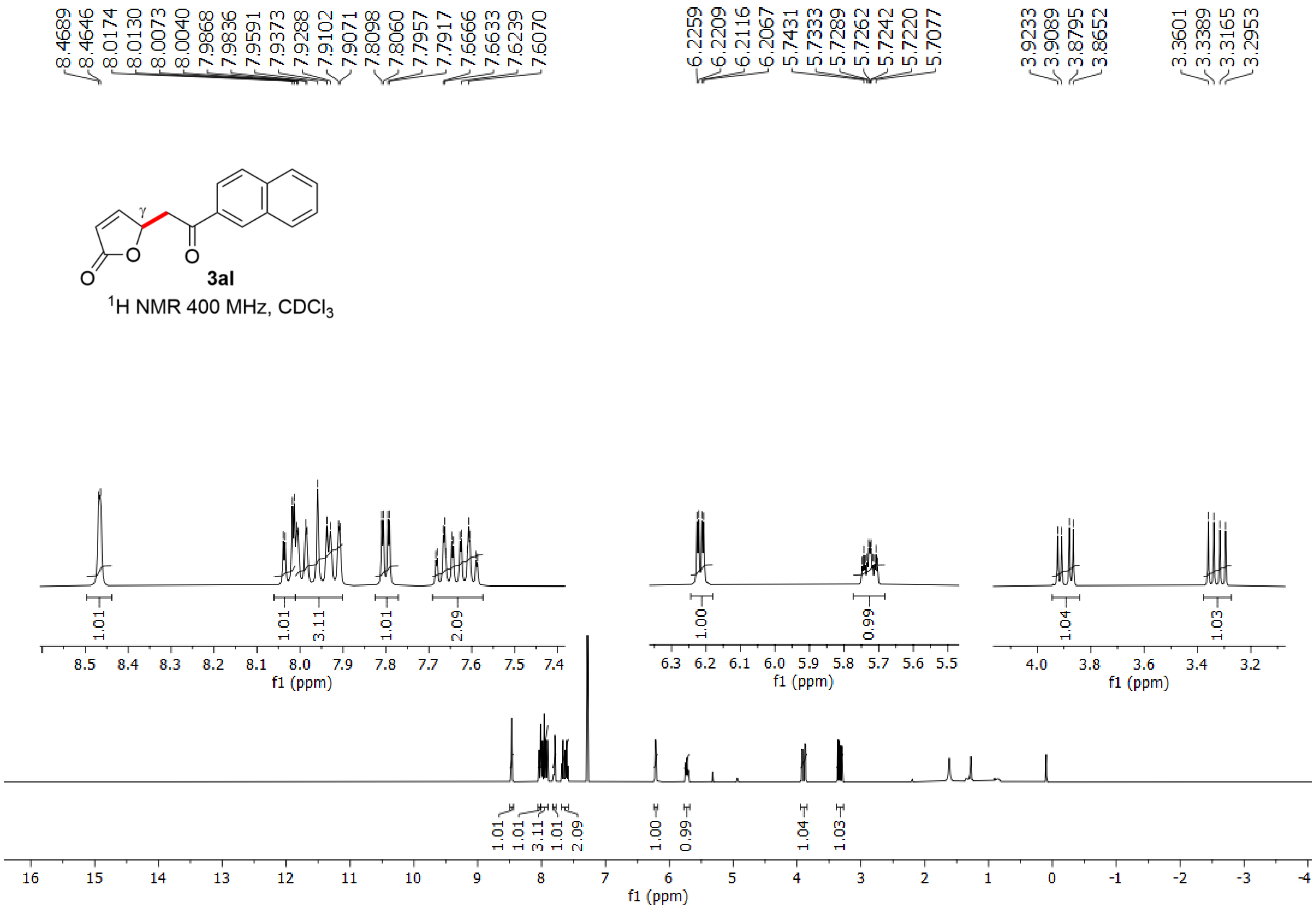
8.4689
8.4646
8.0174
8.0130
8.0073
8.0040
7.9868
7.9836
7.9591
7.9373
7.9288
7.9102
7.9071
7.8098
7.8060
7.7957
7.7917
7.6666
7.6633
7.6239
7.6070
6.2259
6.2209
6.2116
6.2067
5.7431
5.7333
5.7289
5.7262
5.7242
5.7220
5.7077
3.9233
3.9089
3.8795
3.8652
3.3601
3.3389
3.3165
3.2953
γ
O
O
O
3al
$^1$H NMR 400 MHz, $CDCl_3$
1.01
1.01
3.11
1.01
2.09
1.00
0.99
1.04
1.03
f1 (ppm)

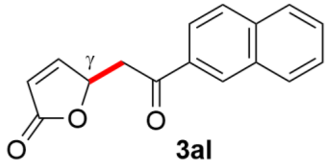

**3al**

$^{13}C$ DEPTq-135 NMR 100 MHz, $CDCl_3$

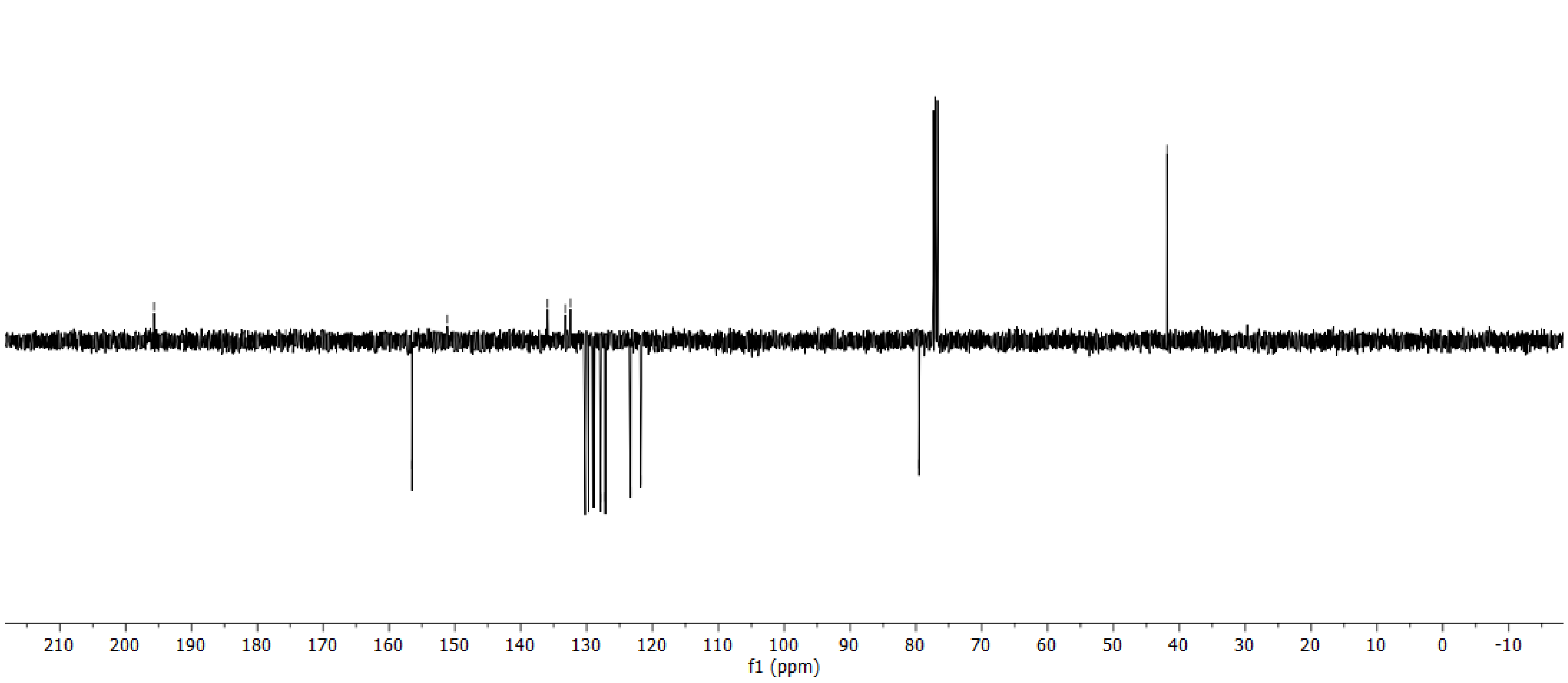

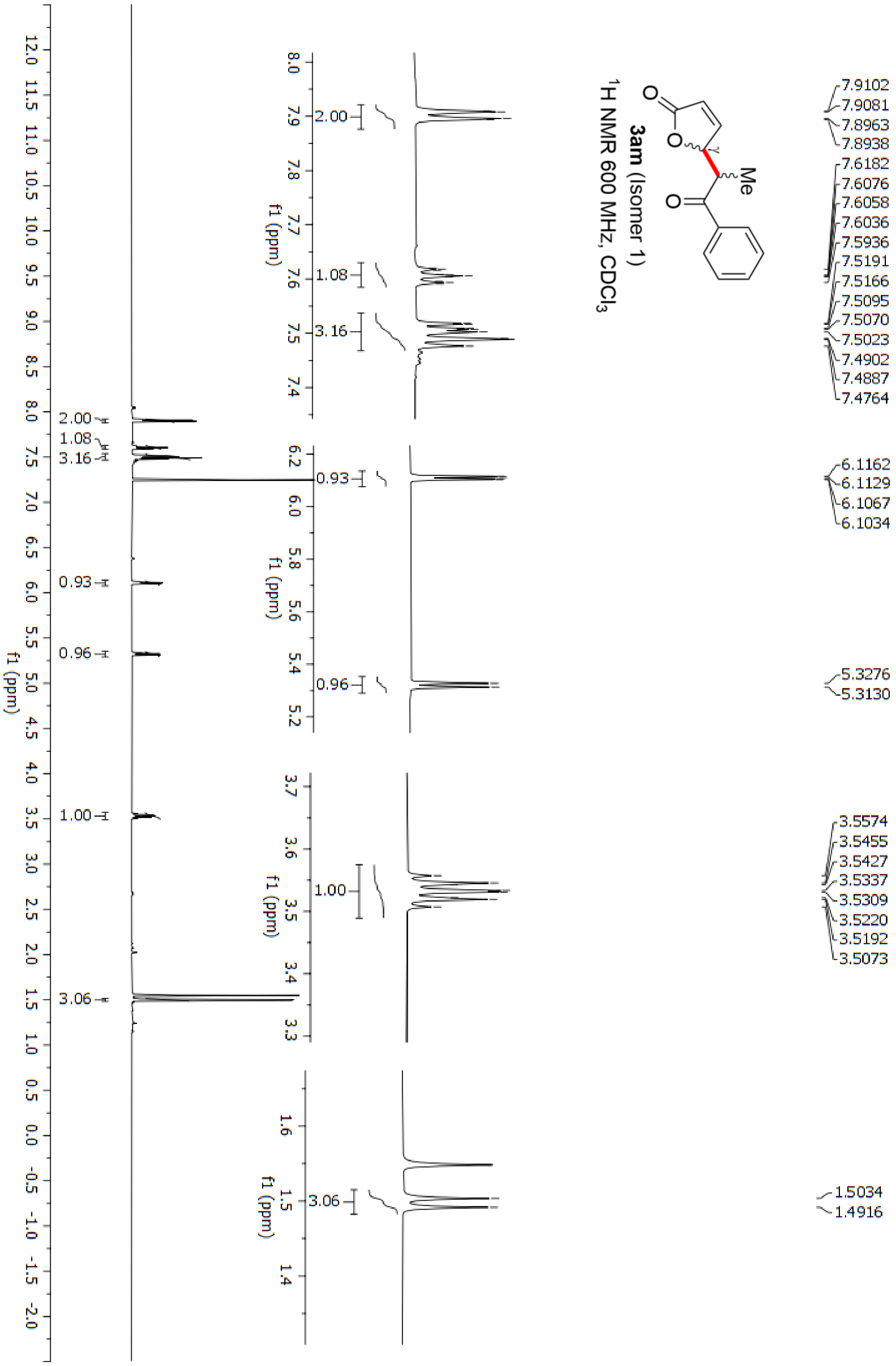
3am (Isomer 1)
1H NMR 600 MHz, CDCl3
Me
7.9102
7.9081
7.8963
7.8938
7.6182
7.6076
7.6058
7.6036
7.5936
7.5191
7.5166
7.5095
7.5070
7.5023
7.4902
7.4887
7.4764
6.1162
6.1129
6.1067
6.1034
5.3276
5.3130
3.5574
3.5455
3.5427
3.5337
3.5309
3.5220
3.5192
3.5073
1.5034
1.4916
f1 (ppm)

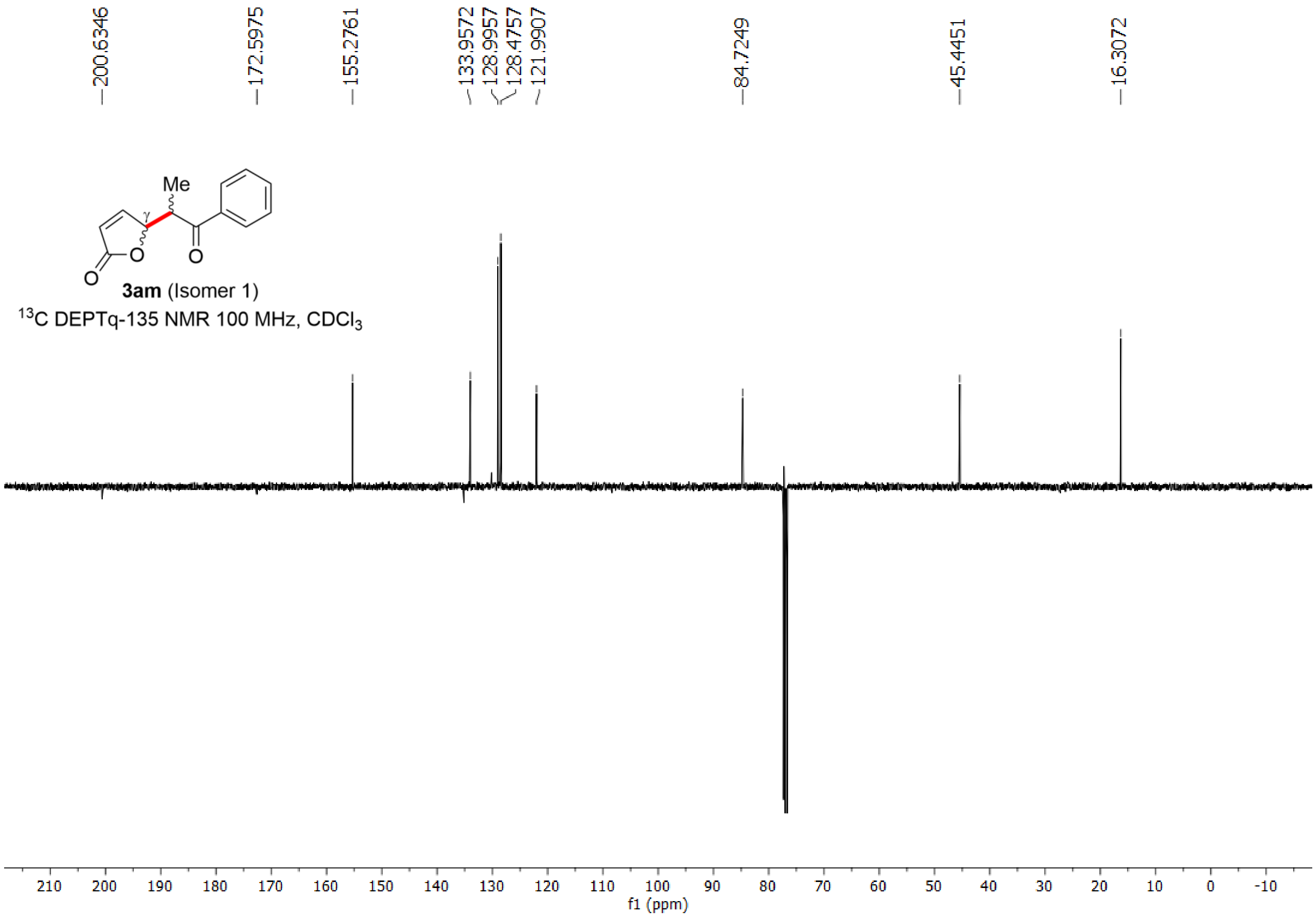
200.6346
172.5975
155.2761
133.9572
128.9957
128.4757
121.9907
84.7249
45.4451
16.3072
Me
γ
O
O
O
3am (Isomer 1)
$^{13}C$ DEPTq-135 NMR 100 MHz, $CDCl_3$
210 200 190 180 170 160 150 140 130 120 110 100 90 80 70 60 50 40 30 20 10 0 -10
f1 (ppm)

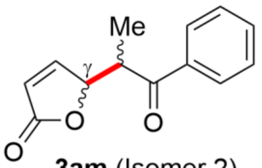

**3am** (Isomer 2)

$^1H$ NMR 400 MHz, $CDCl_3$

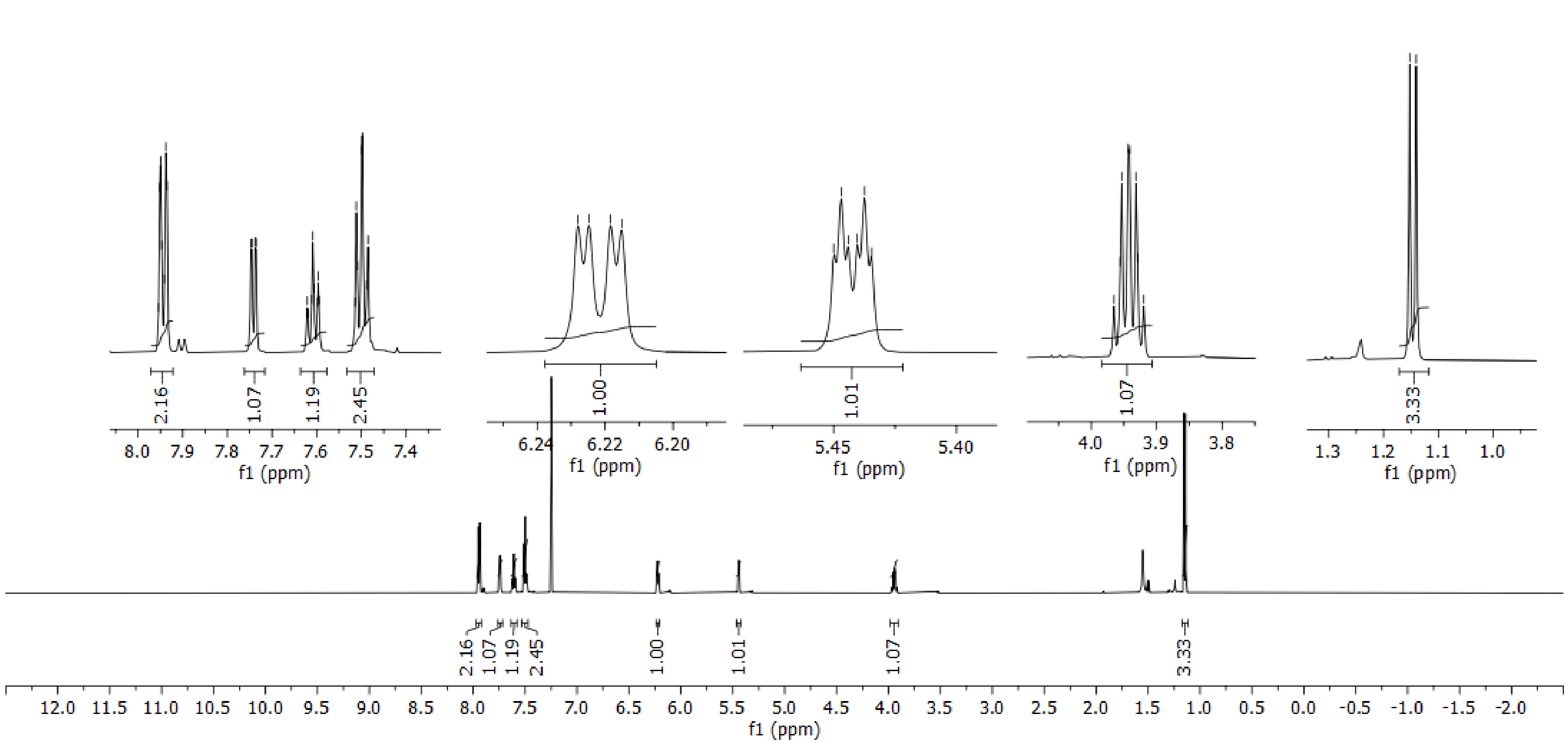

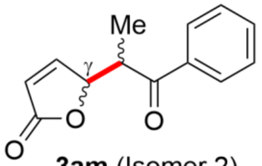


**3am** (Isomer 2)

$^{13}C$ DEPTq-135 NMR 100 MHz, $CDCl_3$

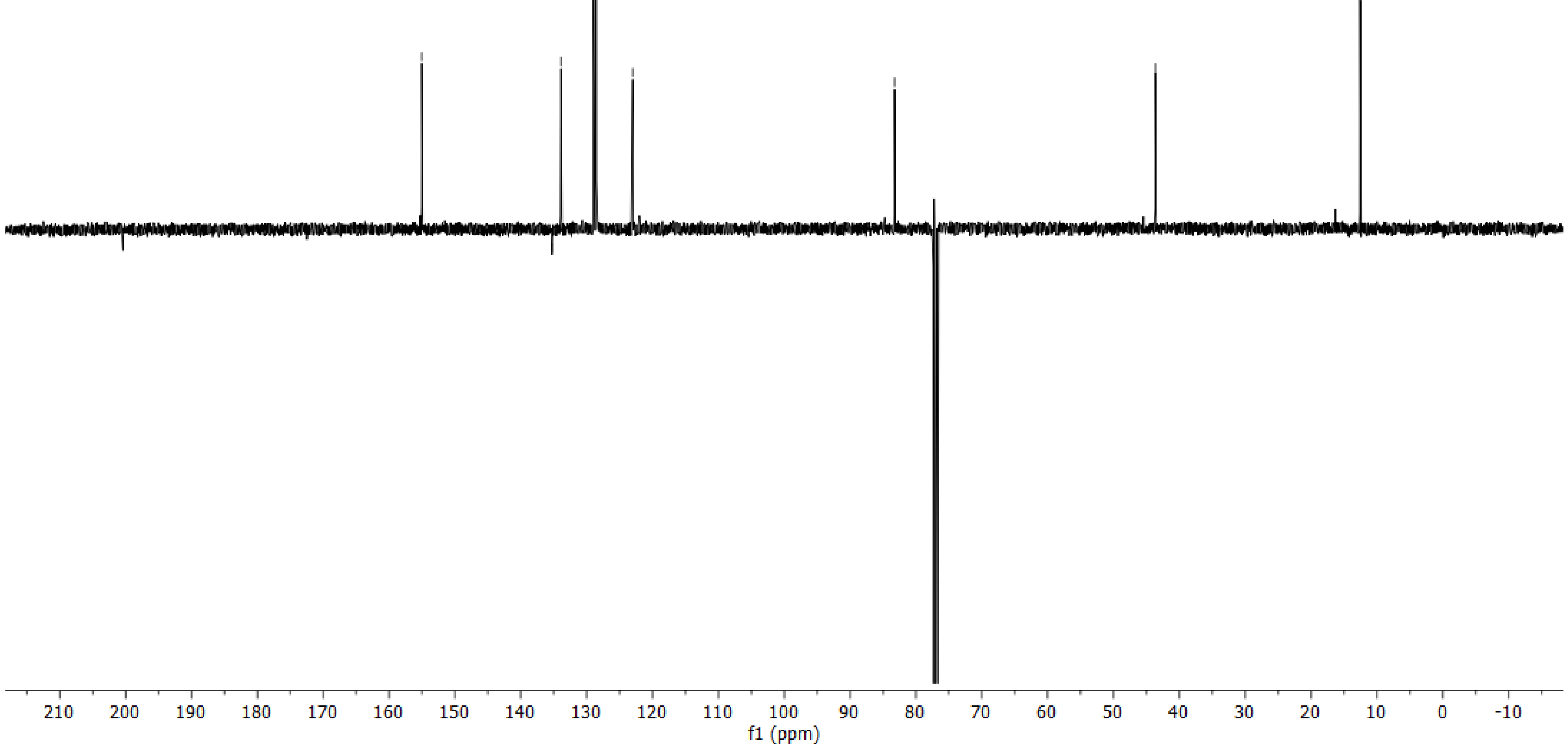

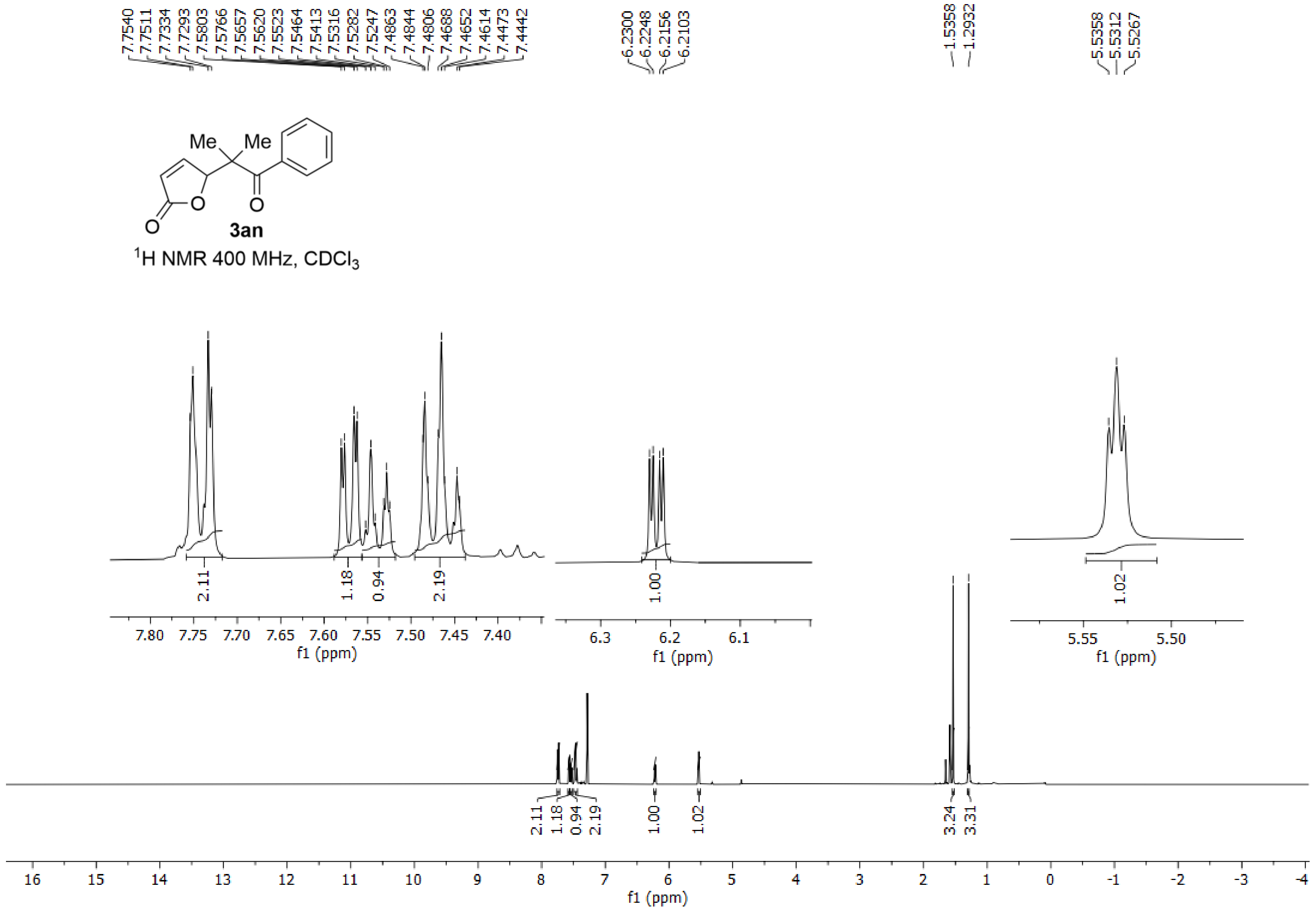

7.7540
7.7511
7.7334
7.7293
7.5803
7.5766
7.5657
7.5620
7.5523
7.5464
7.5413
7.5316
7.5282
7.5247
7.4863
7.4844
7.4806
7.4688
7.4652
7.4614
7.4473
7.4442
6.2300
6.2248
6.2156
6.2103
1.5358
1.2932
5.5358
5.5312
5.5267
Me
Me
O
O
O
3an
¹H NMR 400 MHz, CDCl3
2.11
1.18
0.94
2.19
1.00
1.02
3.24
3.31
f1 (ppm)

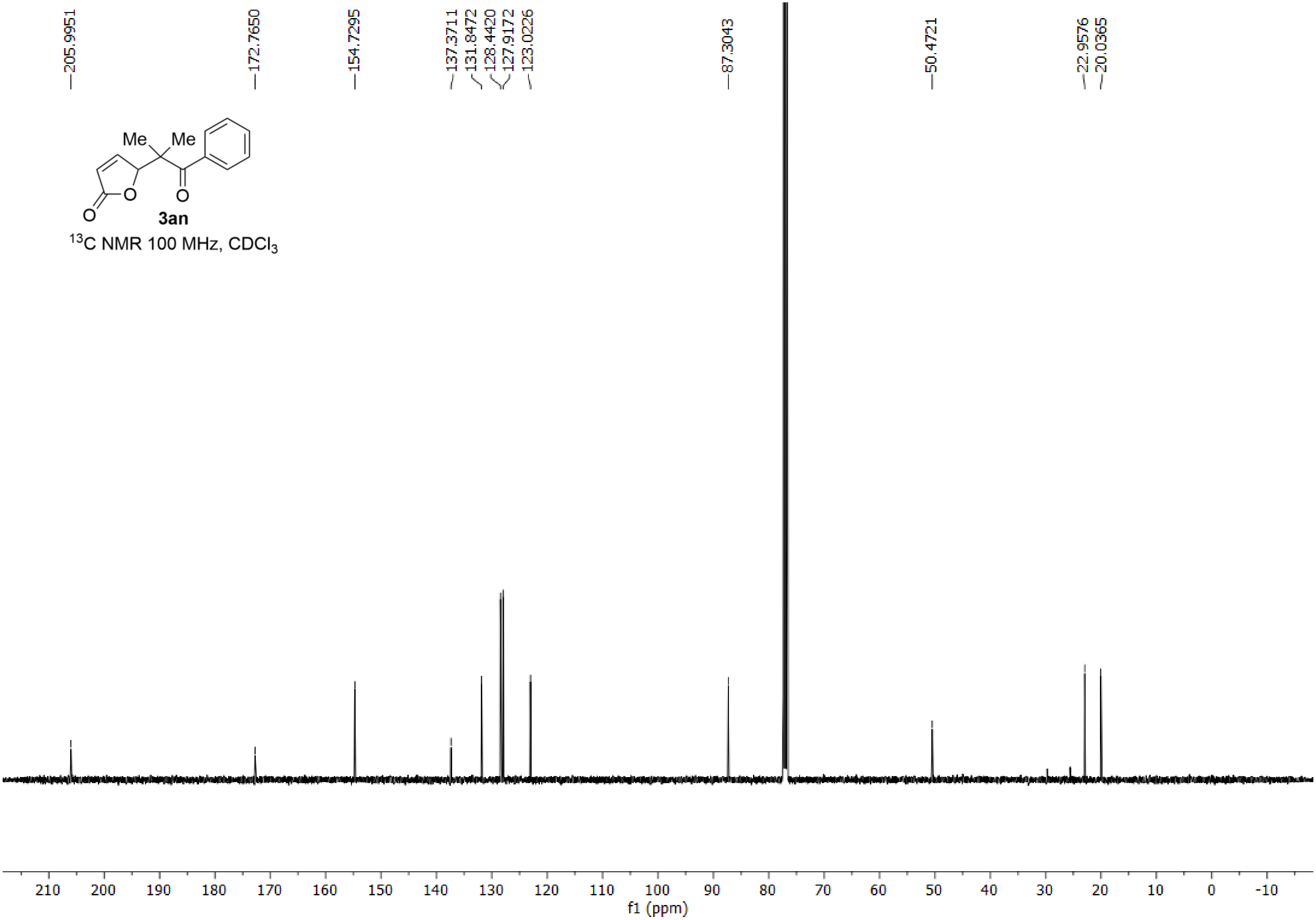
205.9951
172.7650
154.7295
137.3711
131.8472
128.4420
127.9172
123.0226
87.3043
50.4721
22.9576
20.0365
Me
Me
O
O
O
3an
$^{13}$C NMR 100 MHz, $CDCl_3$
210
200
190
180
170
160
150
140
130
120
110
100
90
80
70
60
50
40
30
20
10
0
-10
f1 (ppm)

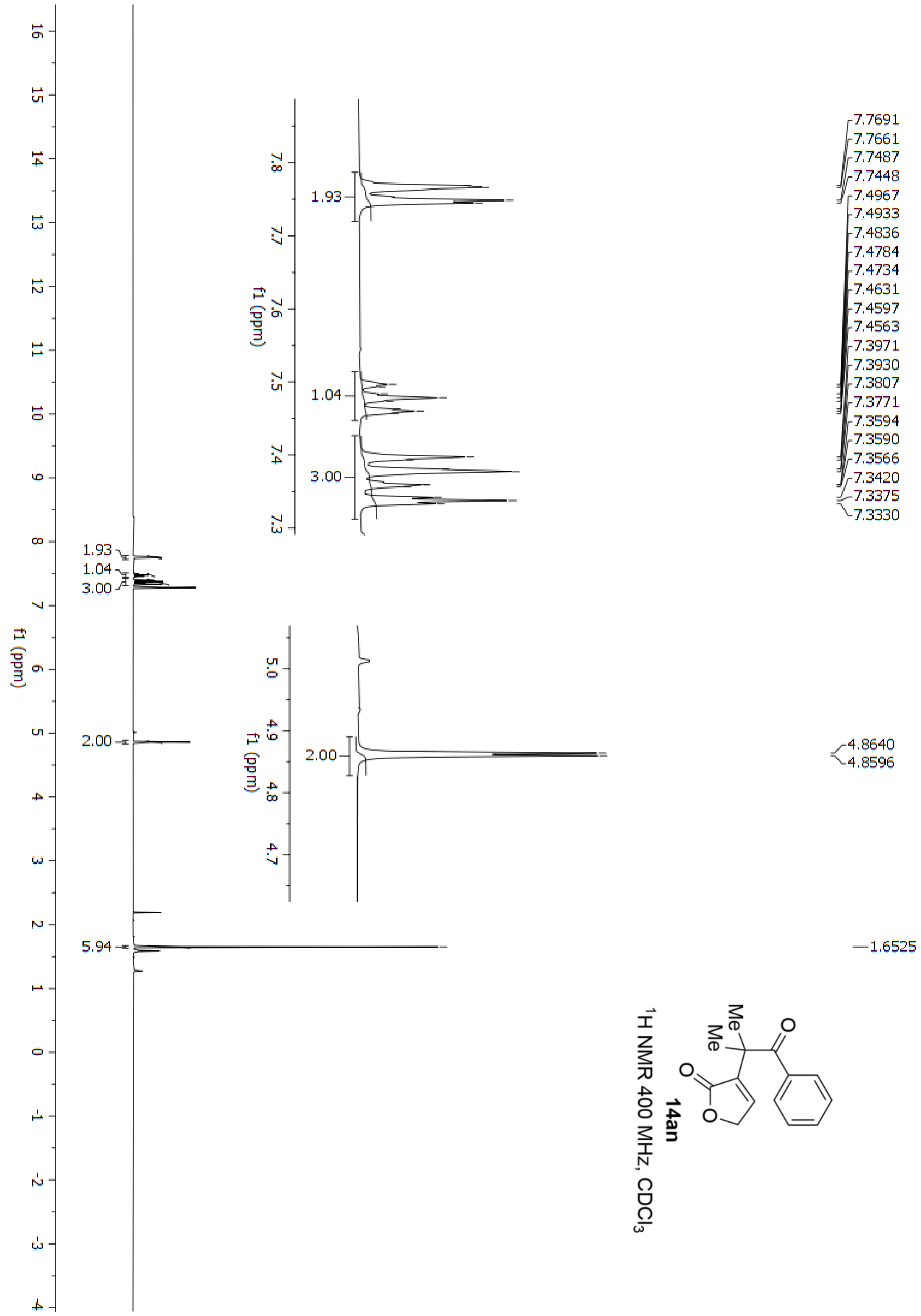

7.7691
7.7661
7.7487
7.7448
7.4967
7.4933
7.4836
7.4784
7.4734
7.4631
7.4597
7.4563
7.3971
7.3930
7.3807
7.3771
7.3594
7.3590
7.3566
7.3420
7.3375
7.3330
4.8640
4.8596
1.6525
1.93
1.04
3.00
2.00
5.94
f1 (ppm)
Me
Me
O
O
O
14an
1H NMR 400 MHz, CDCl3

**14an**

$^{13}C$ NMR 100 MHz, $CDCl_3$

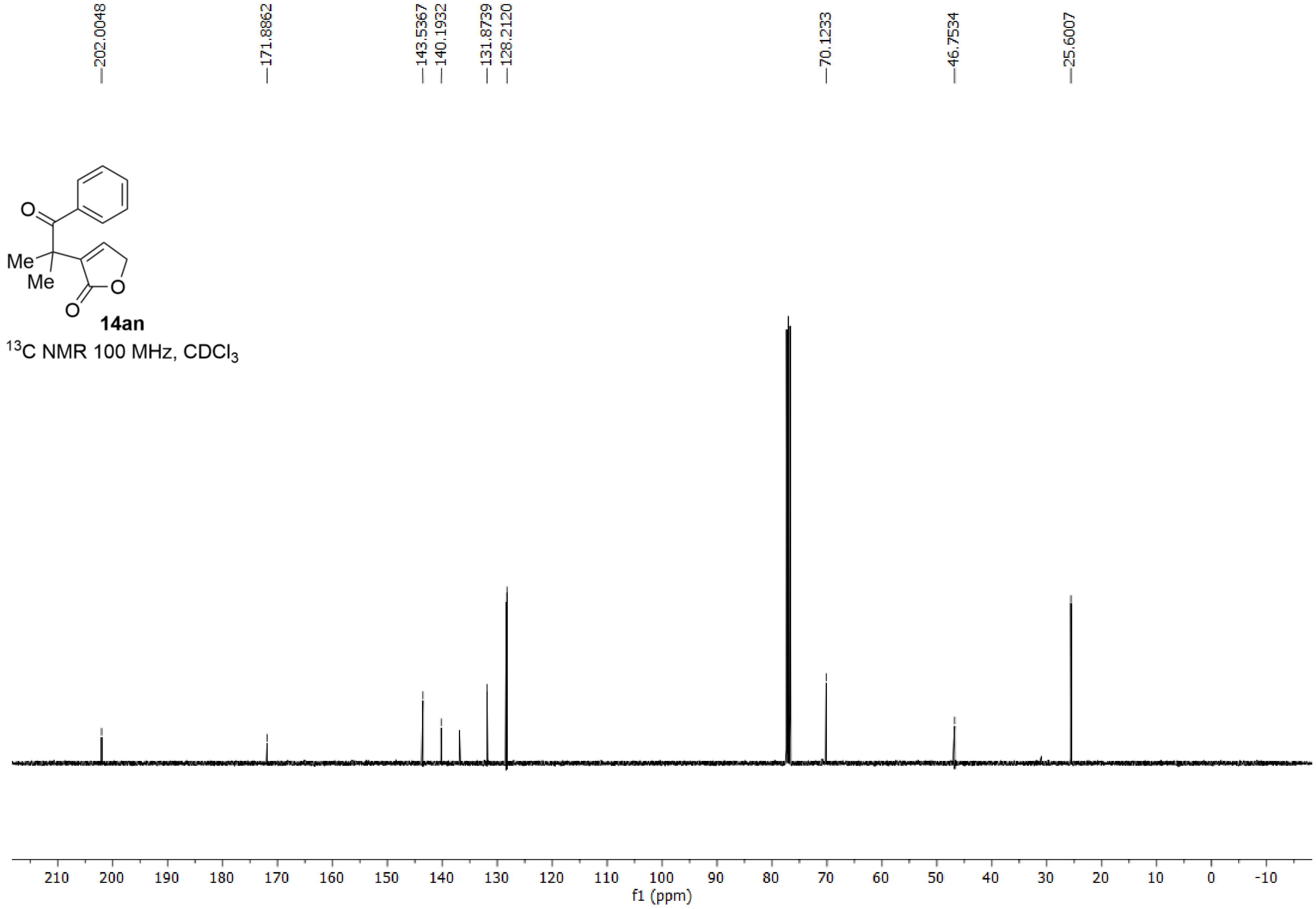

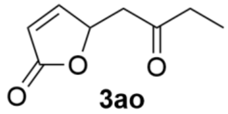

**3ao**

$^{1}$H NMR 400 MHz, $CDCl_3$

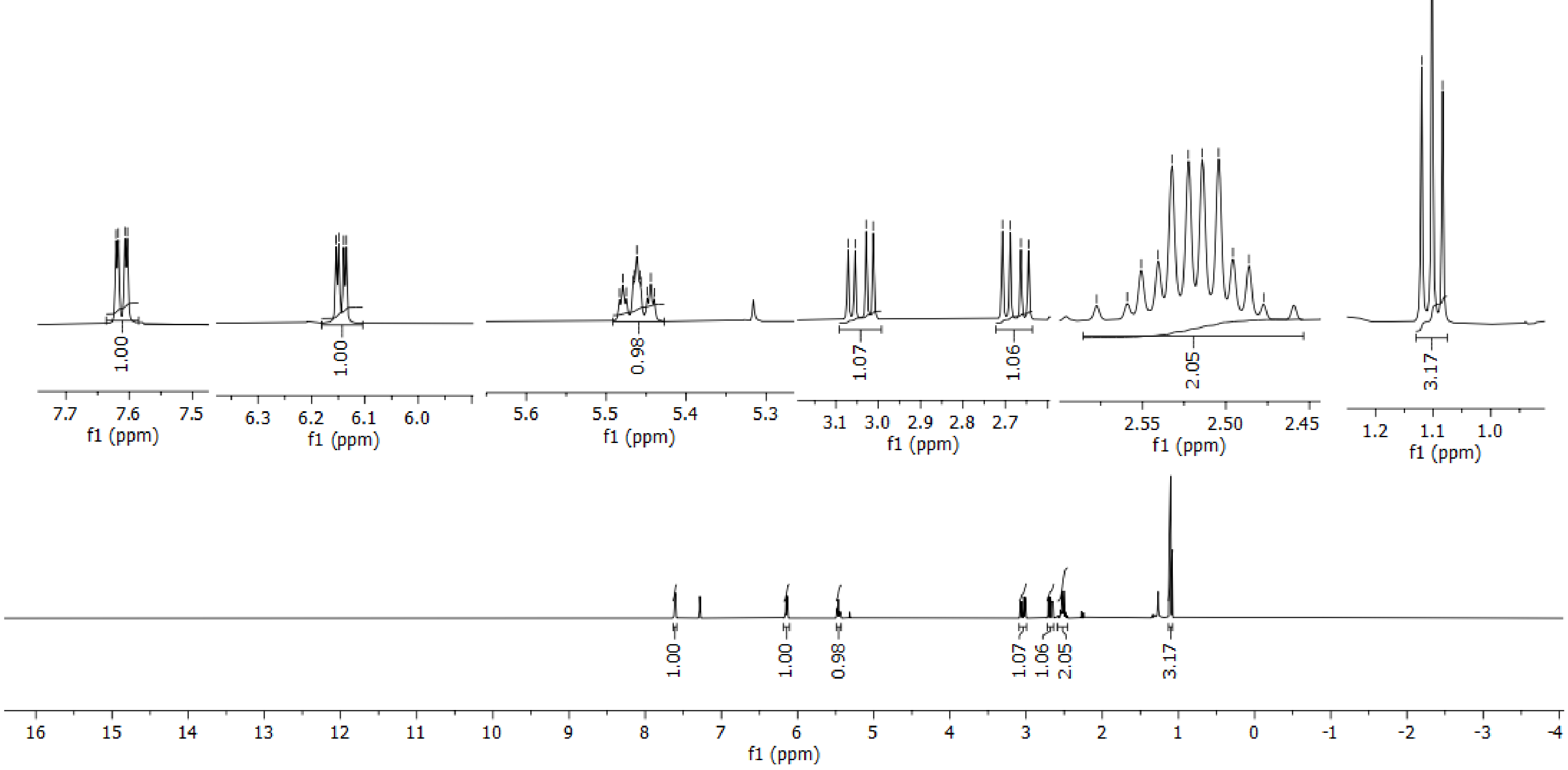

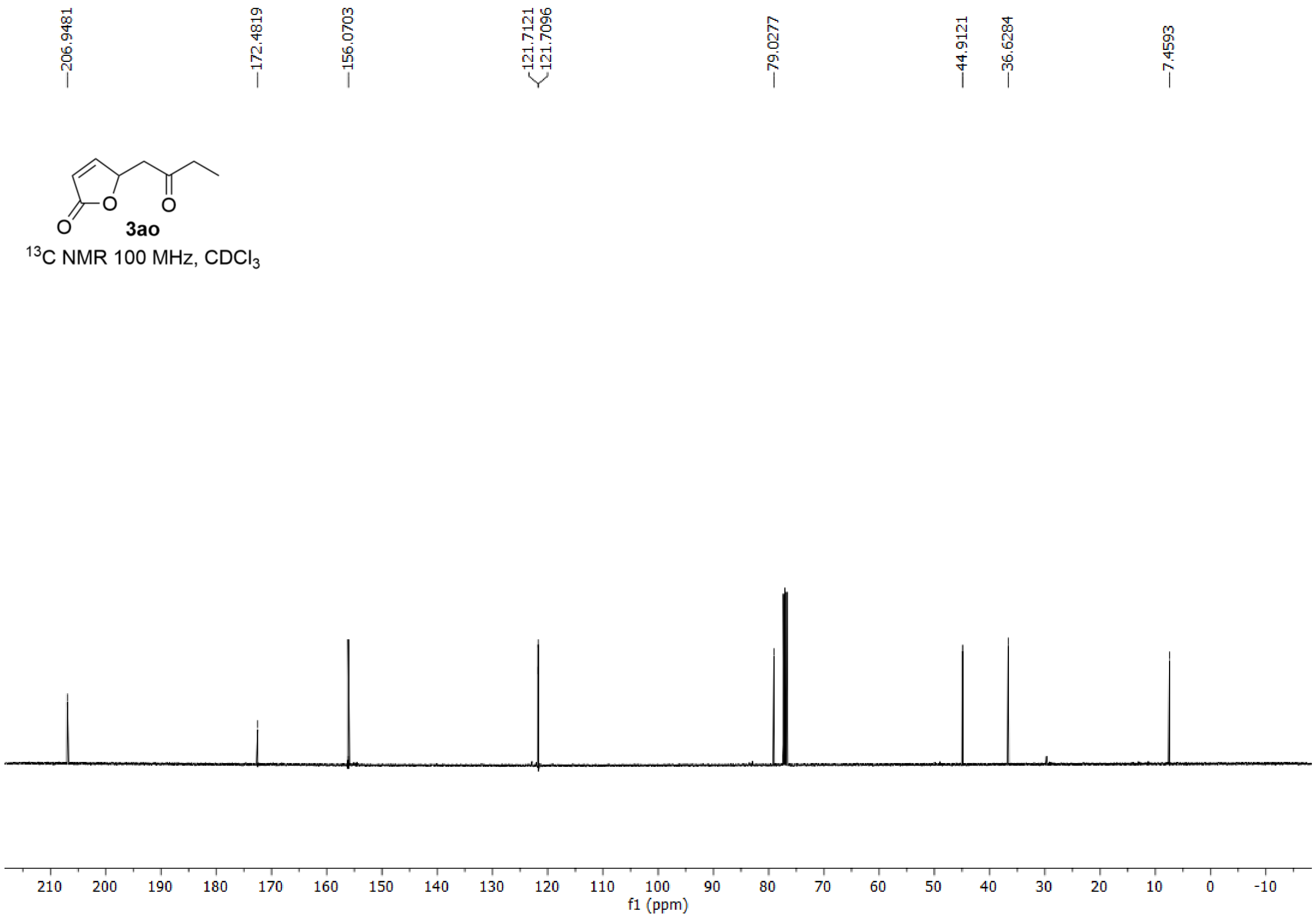
206.9481
172.4819
156.0703
121.7121
121.7096
79.0277
44.9121
36.6284
7.4593
3ao
13C NMR 100 MHz, CDCl3
210 200 190 180 170 160 150 140 130 120 110 100 90 80 70 60 50 40 30 20 10 0 -10
f1 (ppm)

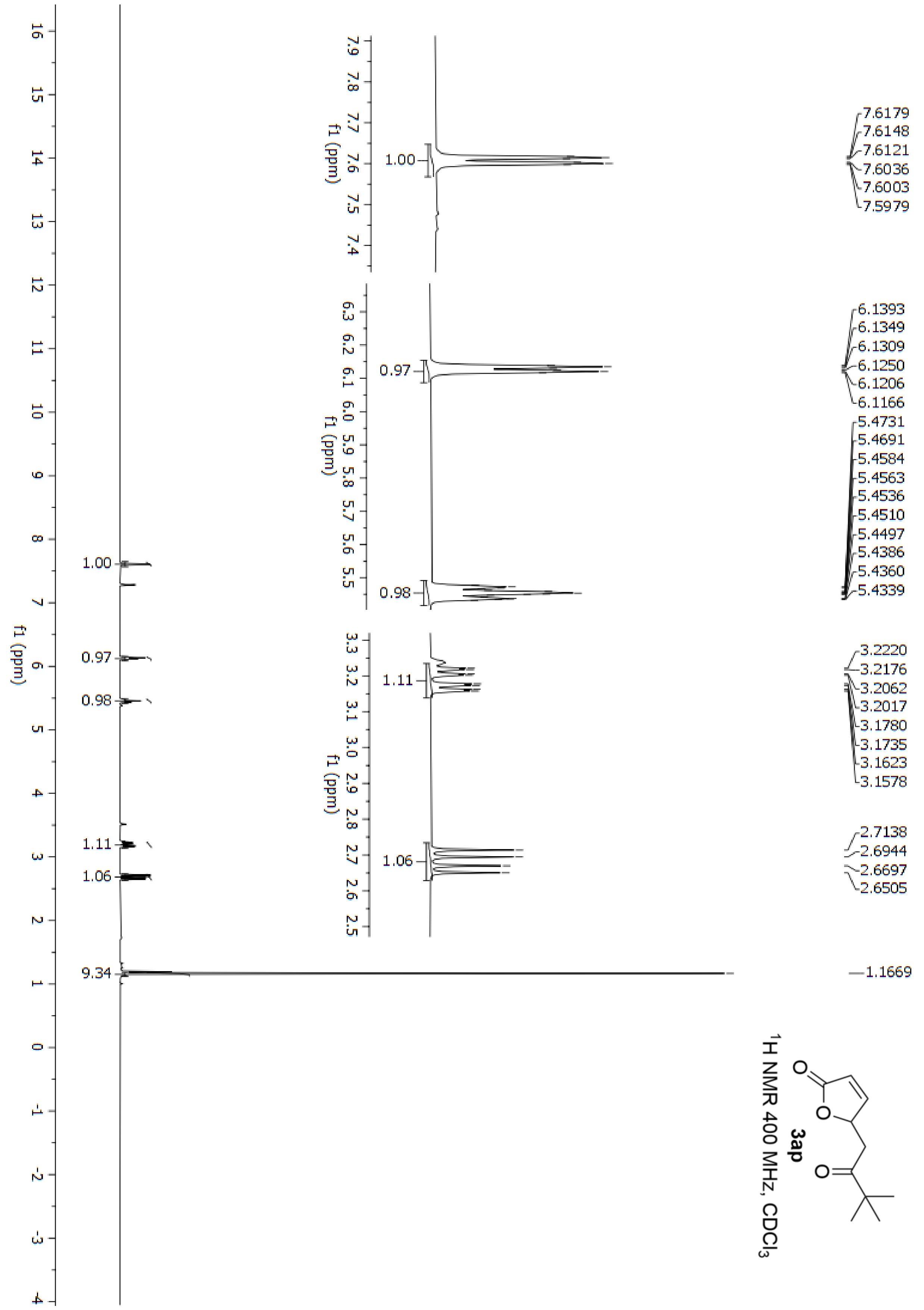


**3ap**

$^1$H NMR 400 MHz, $CDCl_3$

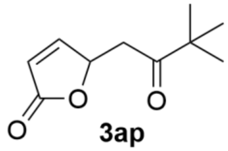

**3ap**

$^{13}C$ NMR 100 MHz, $CDCl_3$

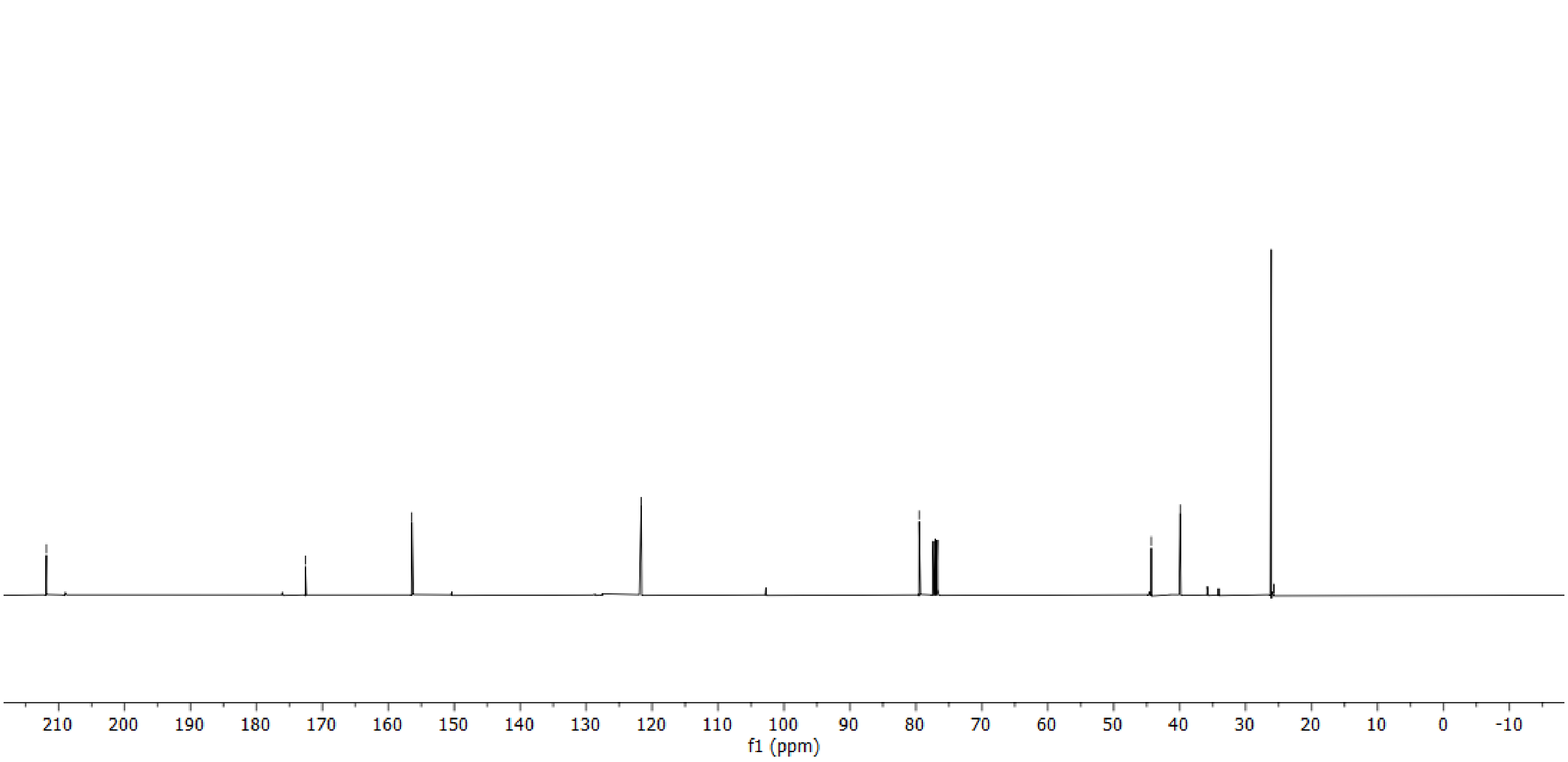

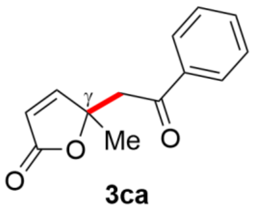


**3ca**

$^1$H NMR 600 MHz, $CDCl_3$

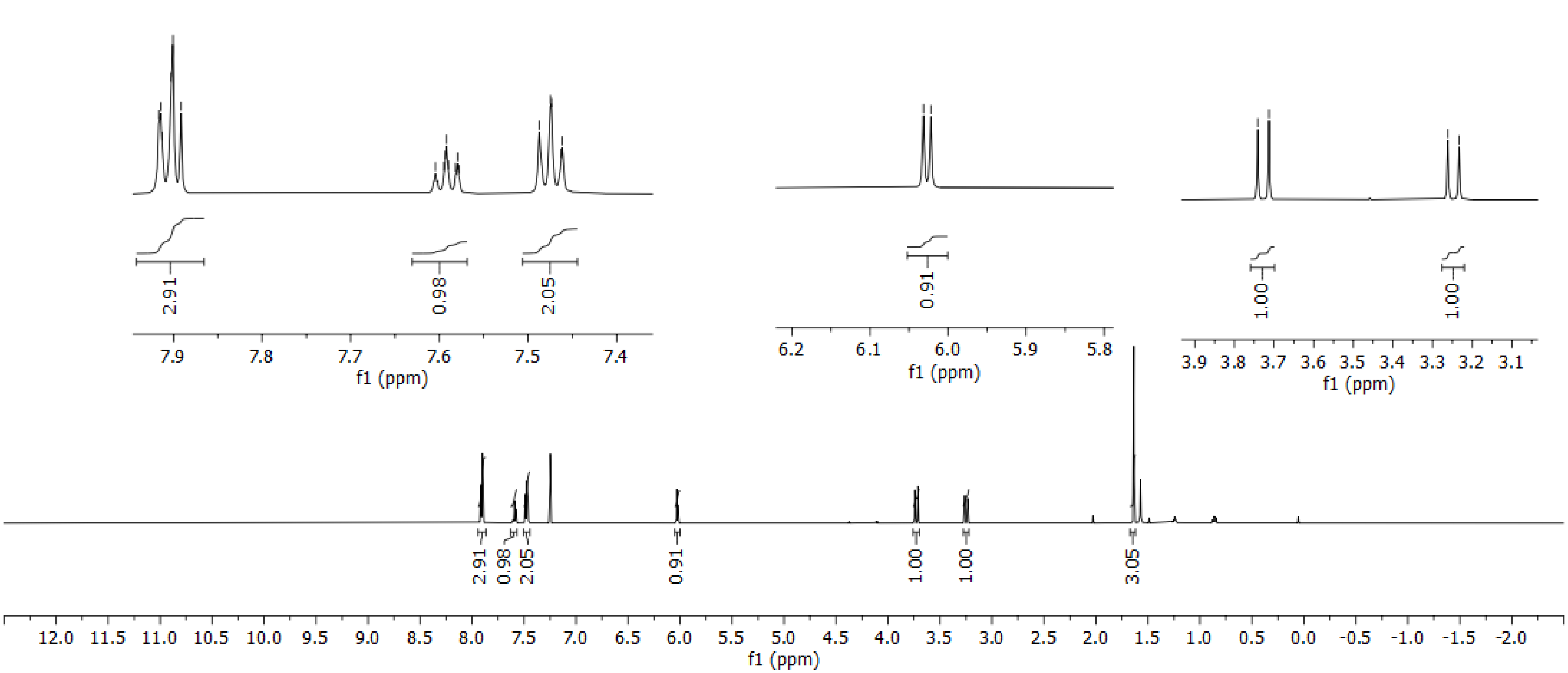

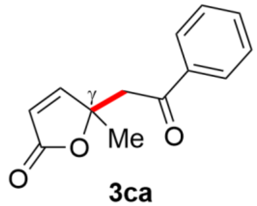


**3ca**

$^{13}C$ NMR 150 MHz, $CDCl_3$

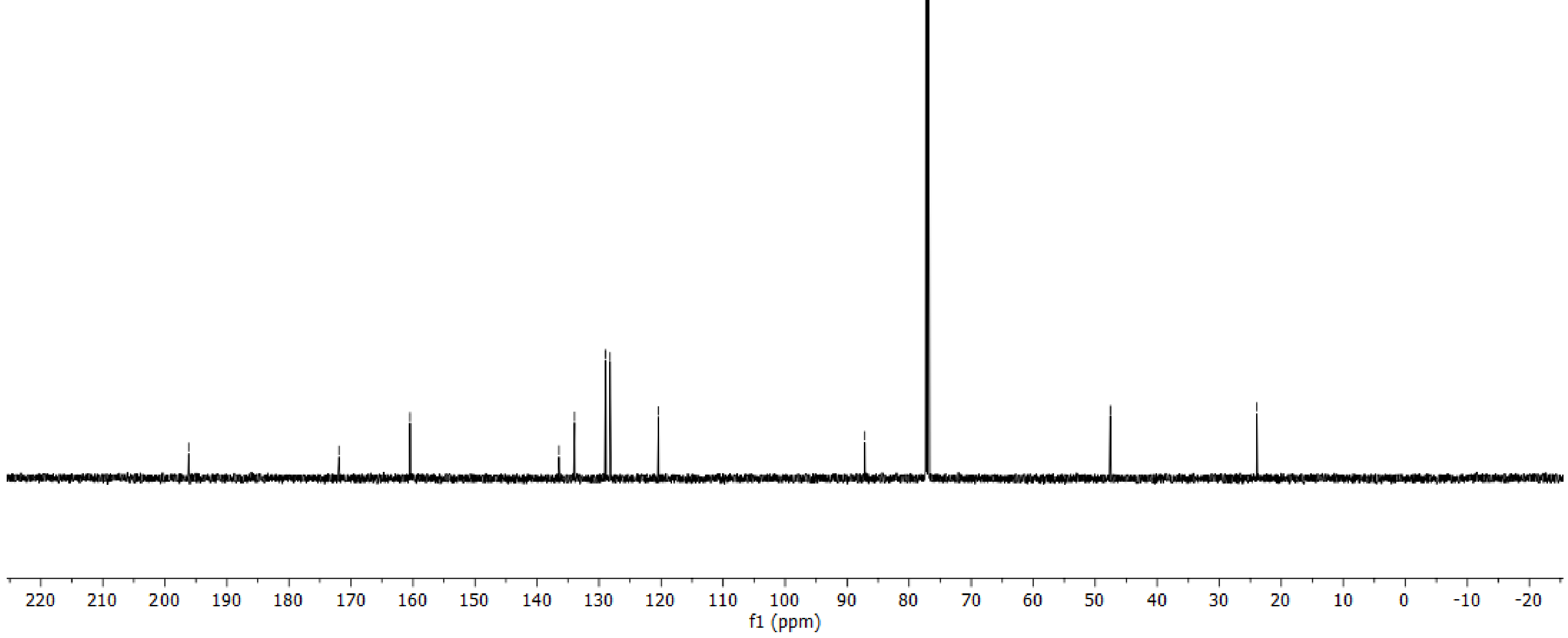

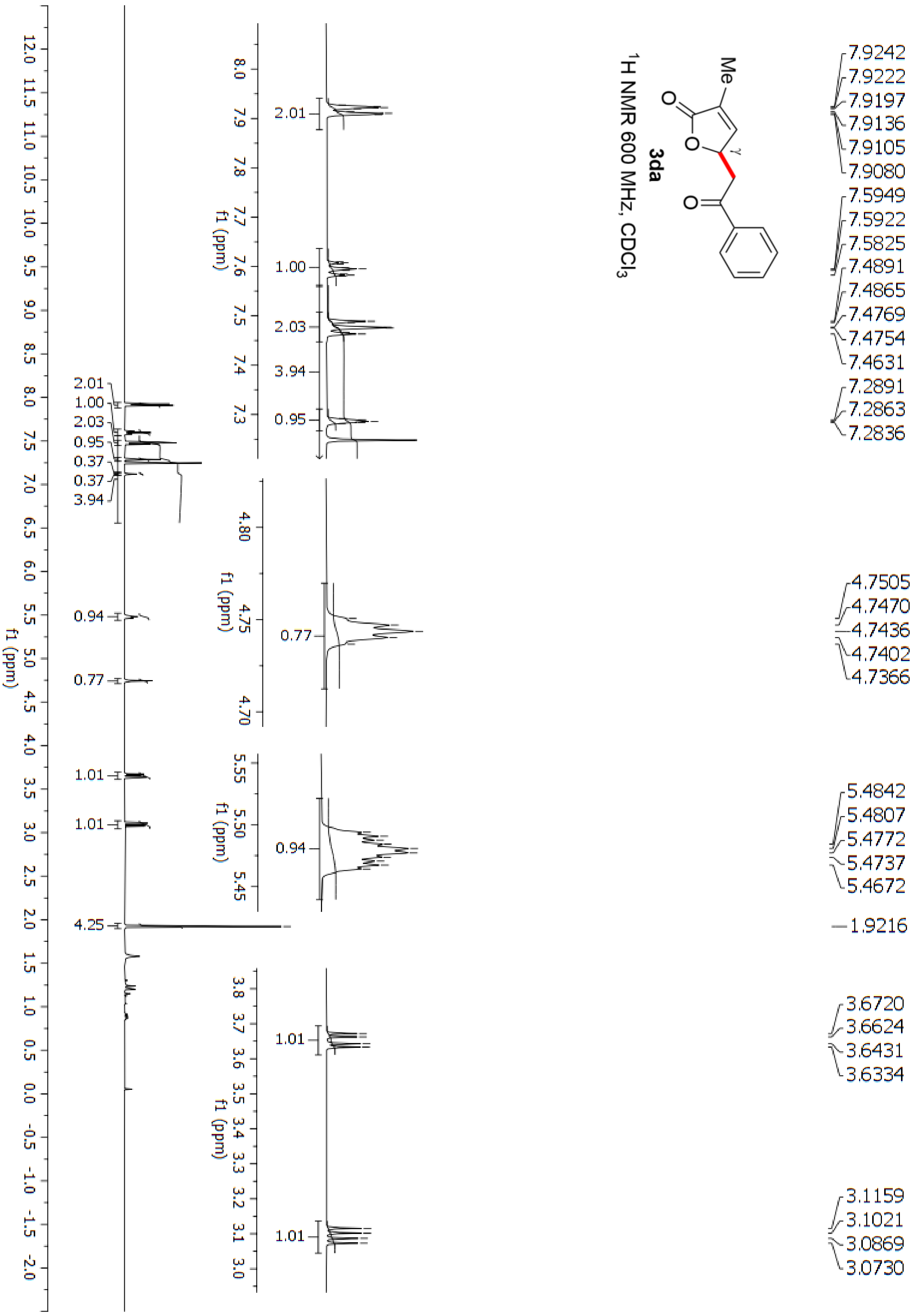

3da
1H NMR 600 MHz, CDCl3
7.9242
7.9222
7.9197
7.9136
7.9105
7.9080
7.5949
7.5922
7.5825
7.4891
7.4865
7.4769
7.4754
7.4631
7.2891
7.2863
7.2836
4.7505
4.7470
4.7436
4.7402
4.7366
5.4842
5.4807
5.4772
5.4737
5.4672
1.9216
3.6720
3.6624
3.6431
3.6334
3.1159
3.1021
3.0869
3.0730
f1 (ppm)

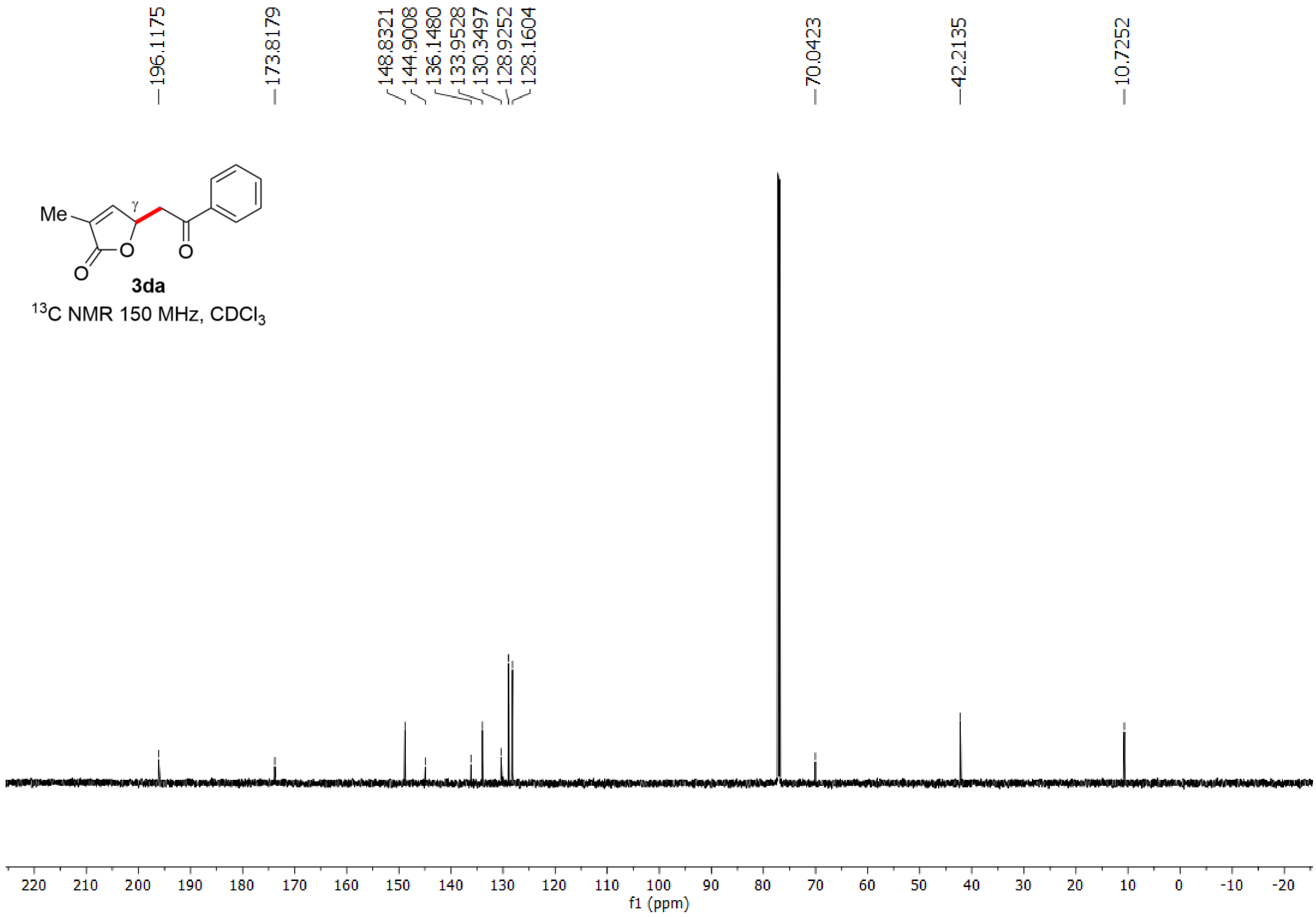
196.1175
173.8179
148.8321
144.9008
136.1480
133.9528
130.3497
128.9252
128.1604
70.0423
42.2135
10.7252
Me
γ
O
O
O
3da
$^{13}$C NMR 150 MHz, $CDCl_3$
220 210 200 190 180 170 160 150 140 130 120 110 100 90 80 70 60 50 40 30 20 10 0 -10 -20
f1 (ppm)

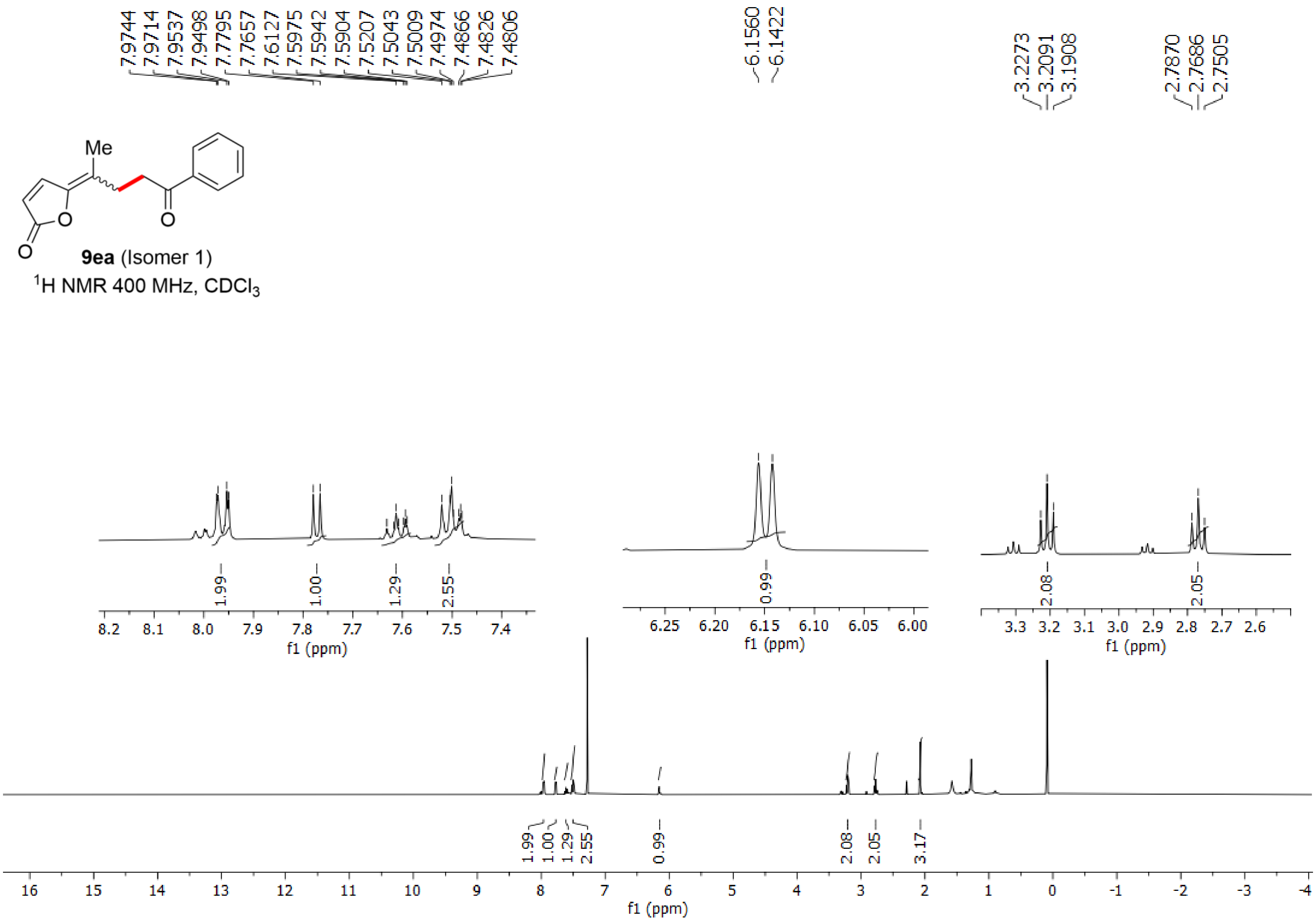
Me
O
O
O
9ea (Isomer 1)
$^1$H NMR 400 MHz, $CDCl_3$
7.9744
7.9714
7.9537
7.9498
7.7795
7.7657
7.6127
7.5975
7.5942
7.5904
7.5207
7.5043
7.5009
7.4974
7.4866
7.4826
7.4806
6.1560
6.1422
3.2273
3.2091
3.1908
2.7870
2.7686
2.7505
1.99
1.00
1.29
2.55
0.99
2.08
2.05
3.17
f1 (ppm)

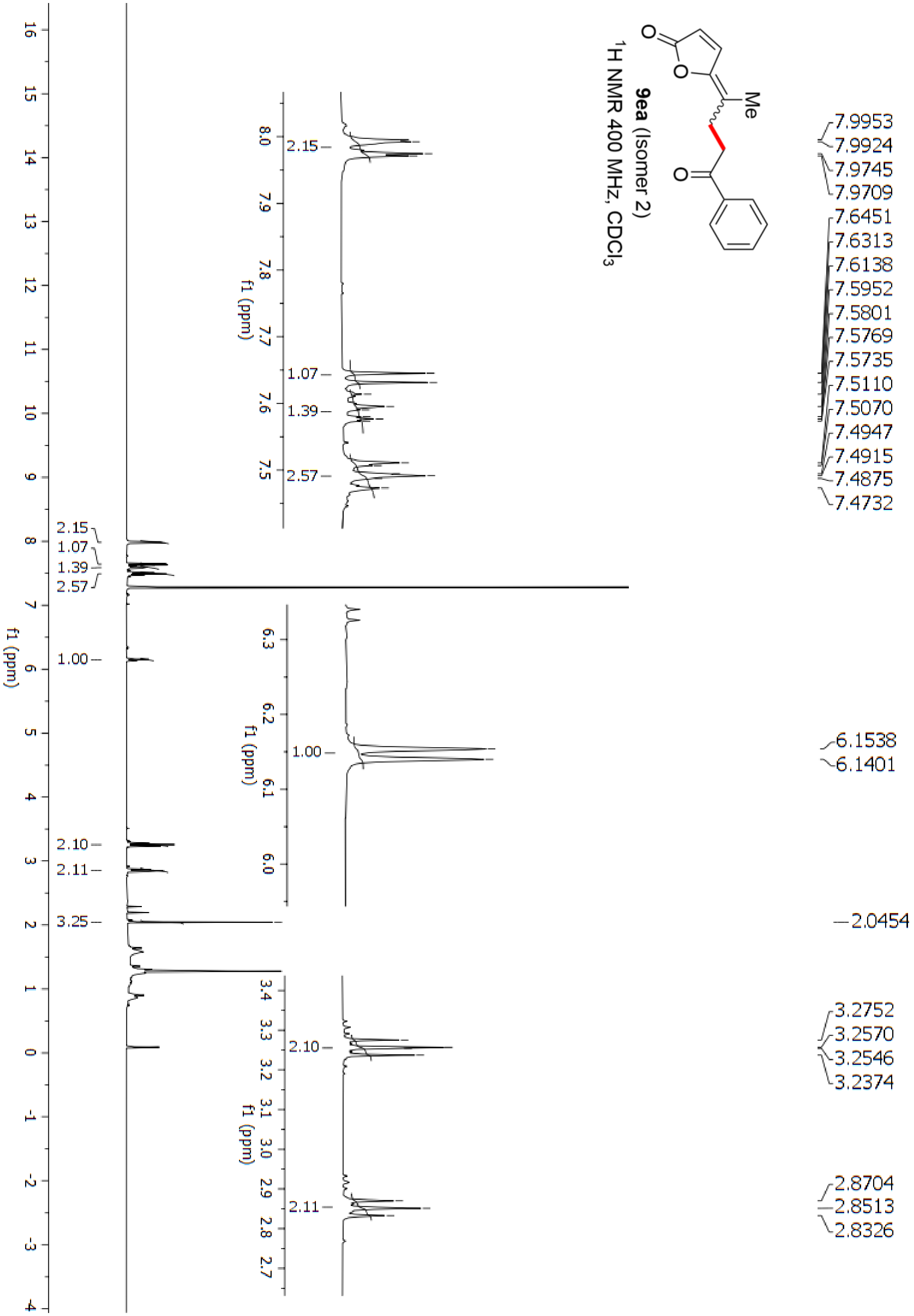

9ea (Isomer 2)
1H NMR 400 MHz, CDCl3
Me
O
7.9953
7.9924
7.9745
7.9709
7.6451
7.6313
7.6138
7.5952
7.5801
7.5769
7.5735
7.5110
7.5070
7.4947
7.4915
7.4875
7.4732
6.1538
6.1401
2.0454
3.2752
3.2570
3.2546
3.2374
2.8704
2.8513
2.8326
2.15
1.07
1.39
2.57
1.00
2.10
2.11
3.25
f1 (ppm)

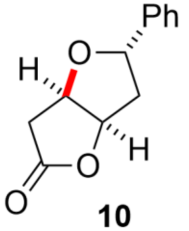


**10**

$^{1}$H NMR 400 MHz, $CDCl_3$

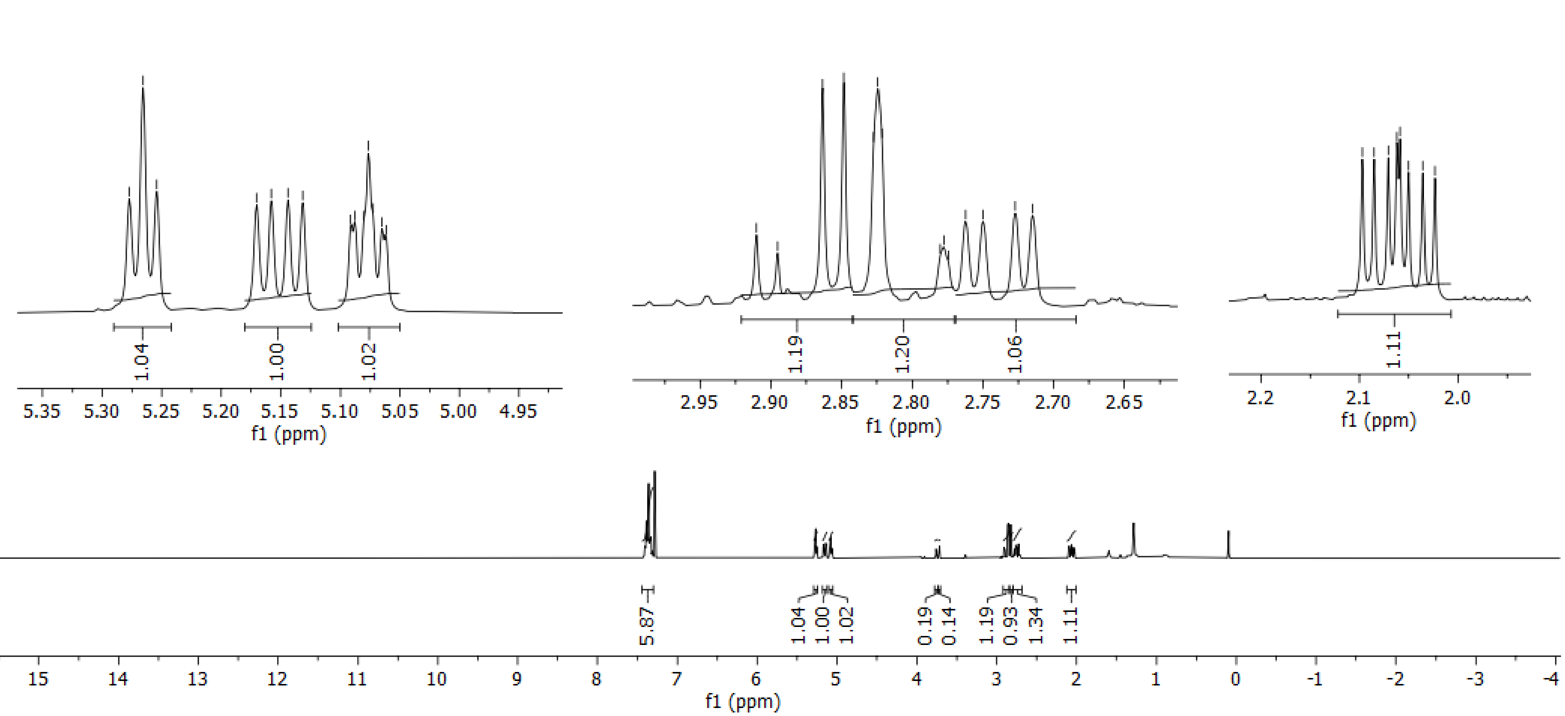

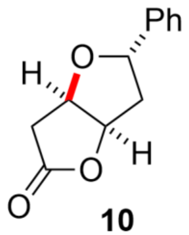


**10**

$^{13}C$ DEPTq-135 NMR 101 MHz, $CDCl_3$

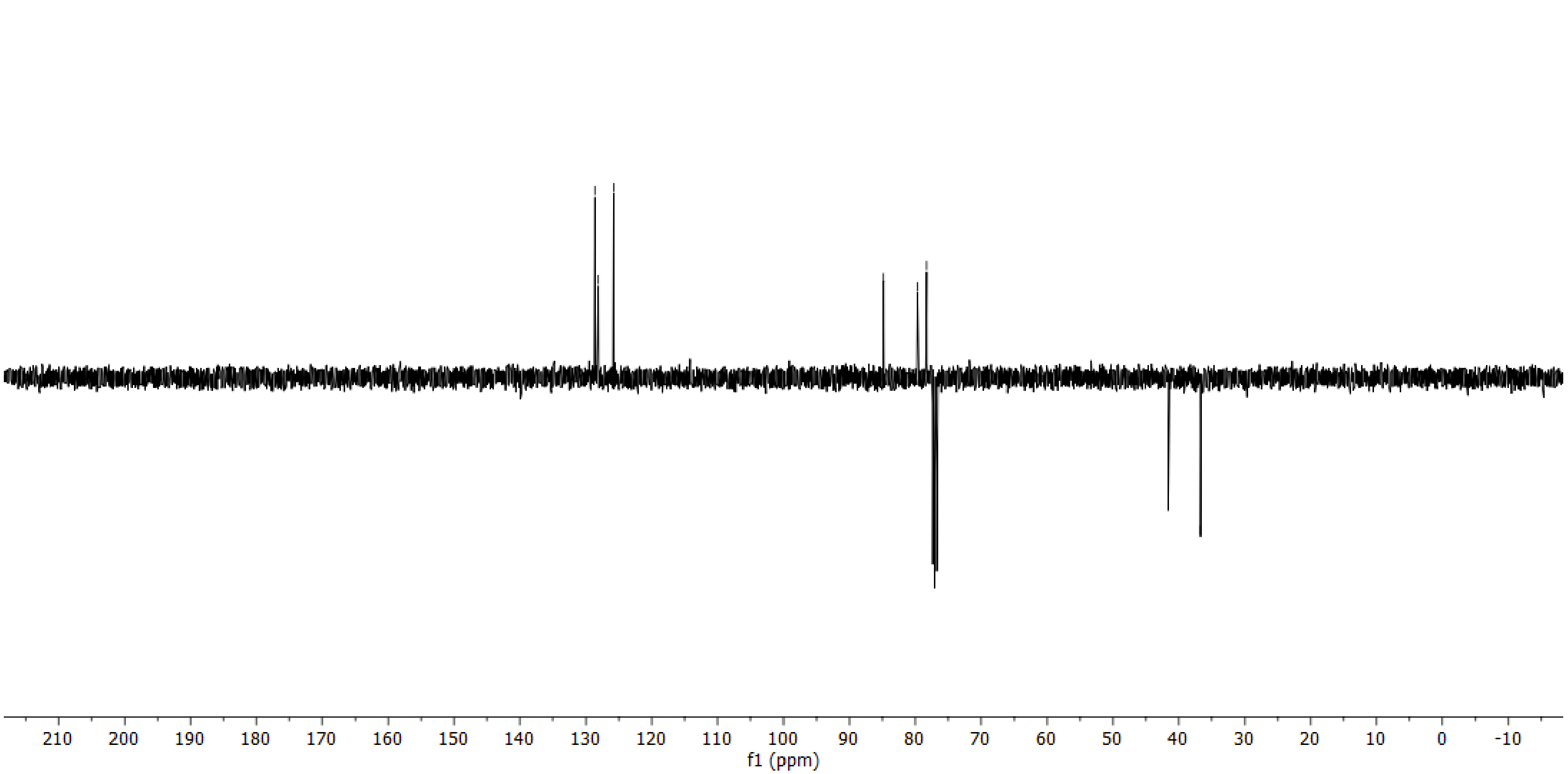

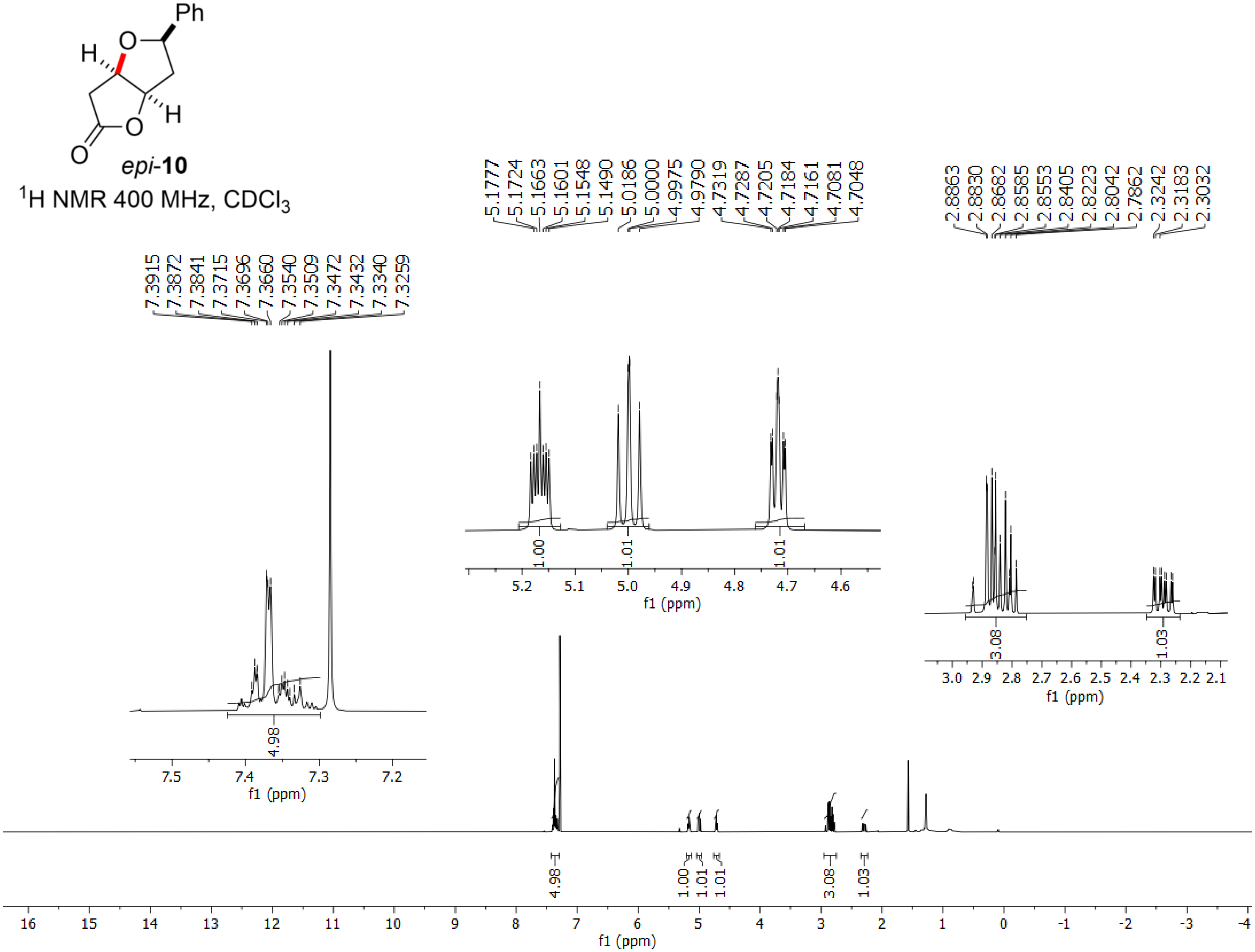


*epi*-**10**

$^1$H NMR 400 MHz, $CDCl_3$

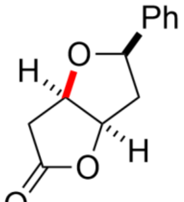

*epi*-**10**

$^{13}C$ DEPTq-135 NMR 101 MHz, $CDCl_3$

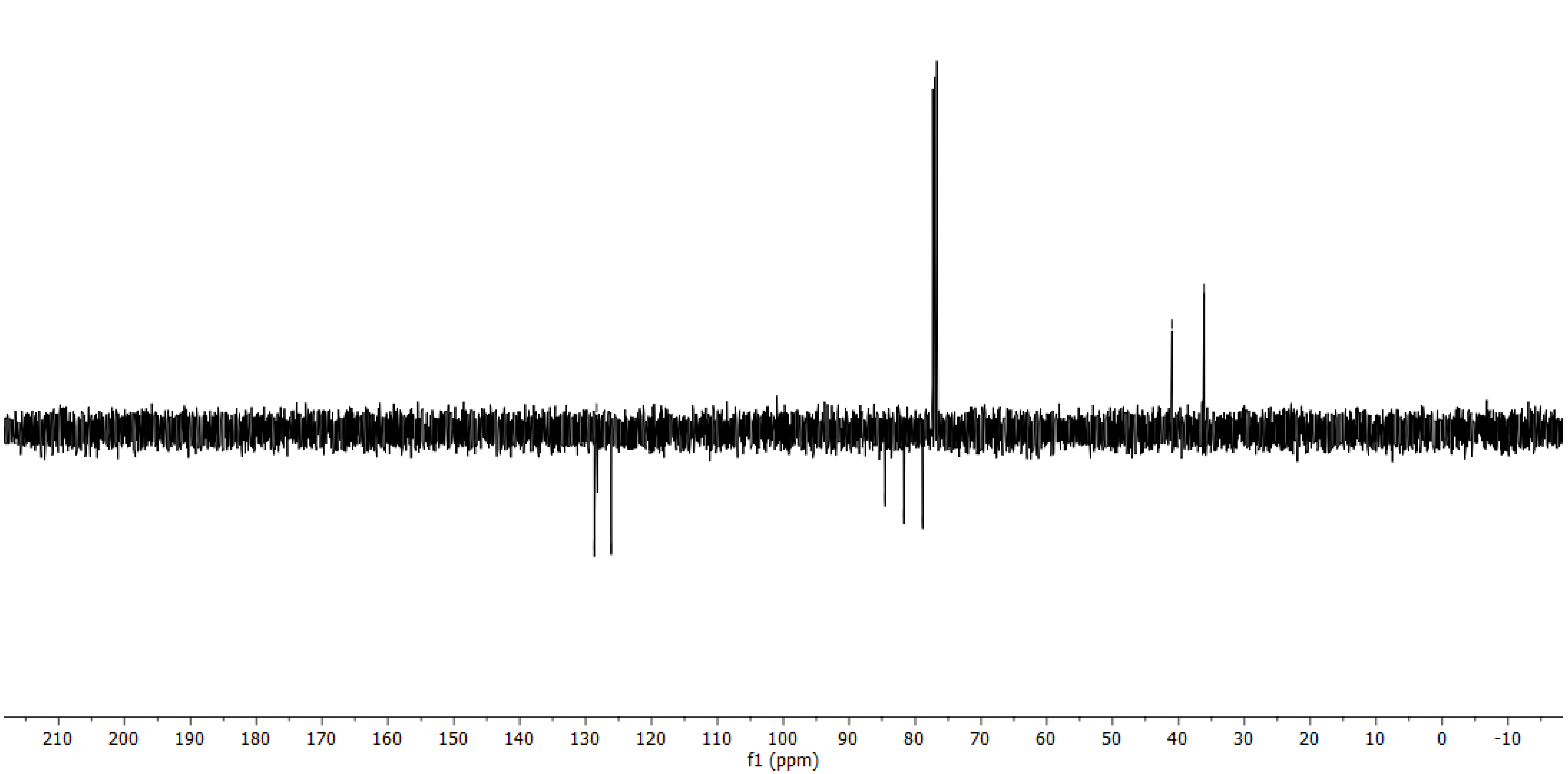

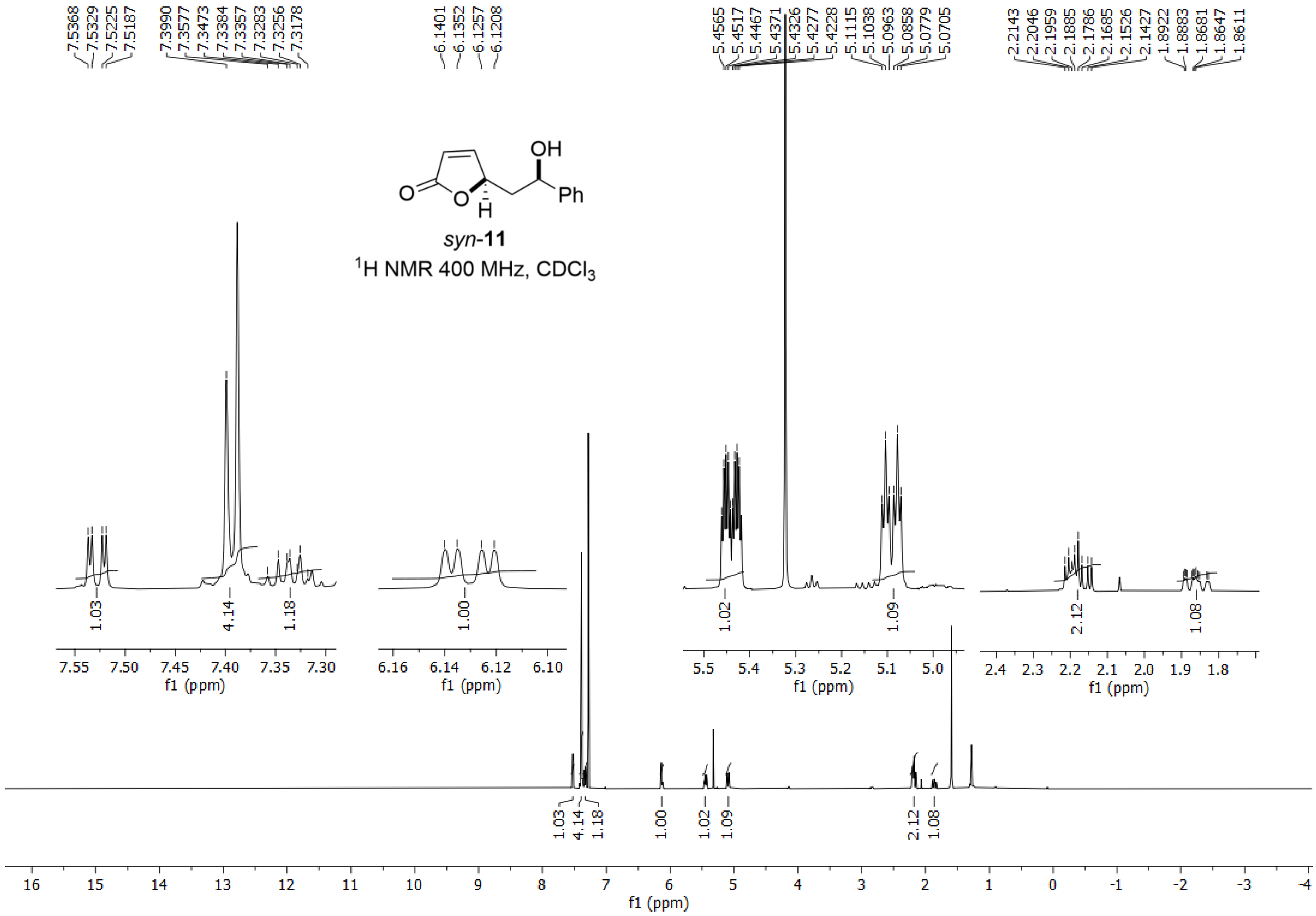
7.5368
7.5329
7.5225
7.5187
7.3990
7.3577
7.3473
7.3384
7.3357
7.3283
7.3256
7.3178
6.1401
6.1352
6.1257
6.1208
5.4565
5.4517
5.4467
5.4371
5.4326
5.4277
5.4228
5.1115
5.1038
5.0963
5.0858
5.0779
5.0705
2.2143
2.2046
2.1959
2.1885
2.1786
2.1685
2.1526
2.1427
1.8922
1.8883
1.8681
1.8647
1.8611
OH
O
O
H
Ph
syn-11
1H NMR 400 MHz, CDCl3
1.03
4.14
1.18
1.00
1.02
1.09
2.12
1.08
f1 (ppm)

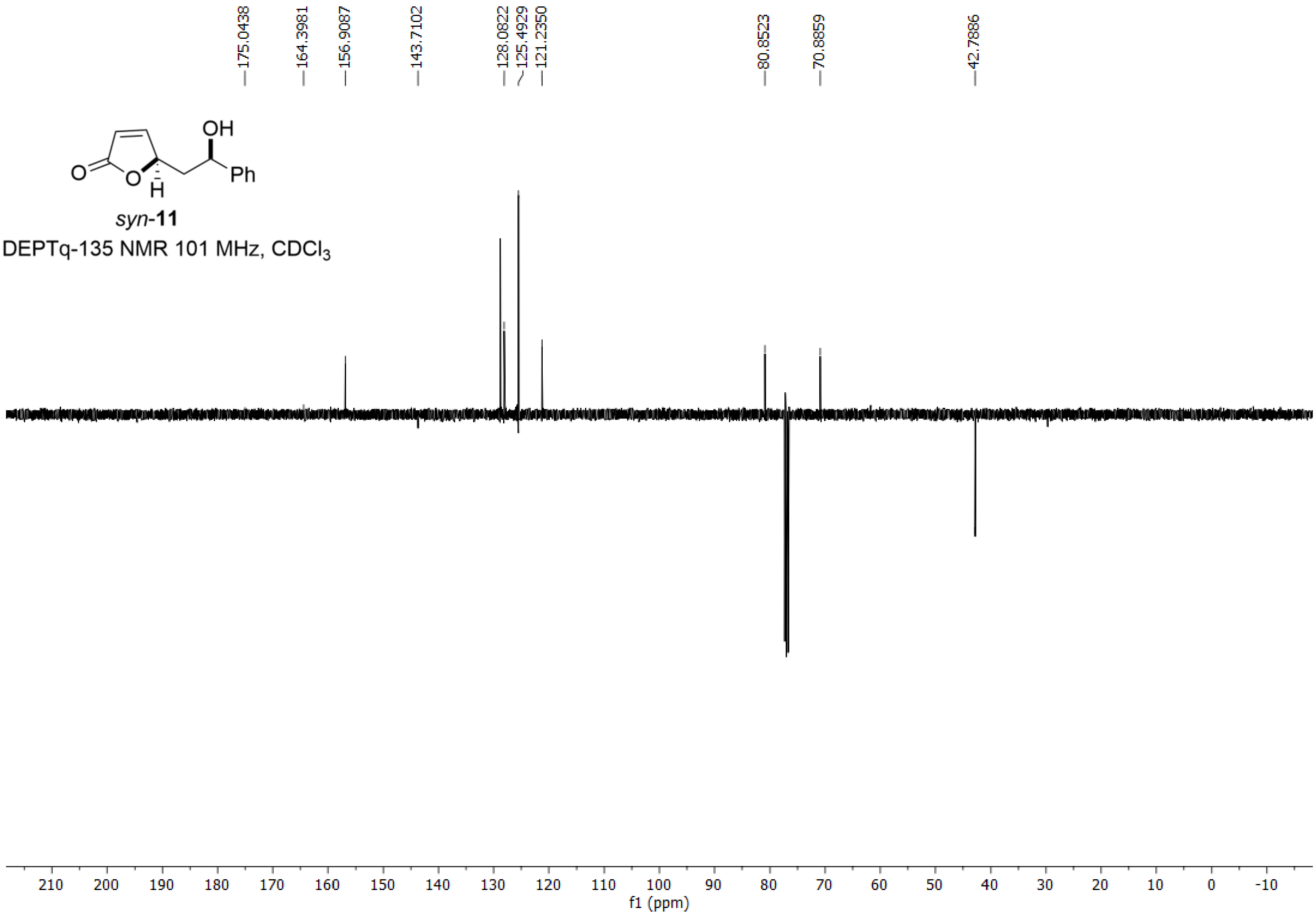


*syn*-**11**

$^{13}C$ DEPTq-135 NMR 101 MHz, $CDCl_3$

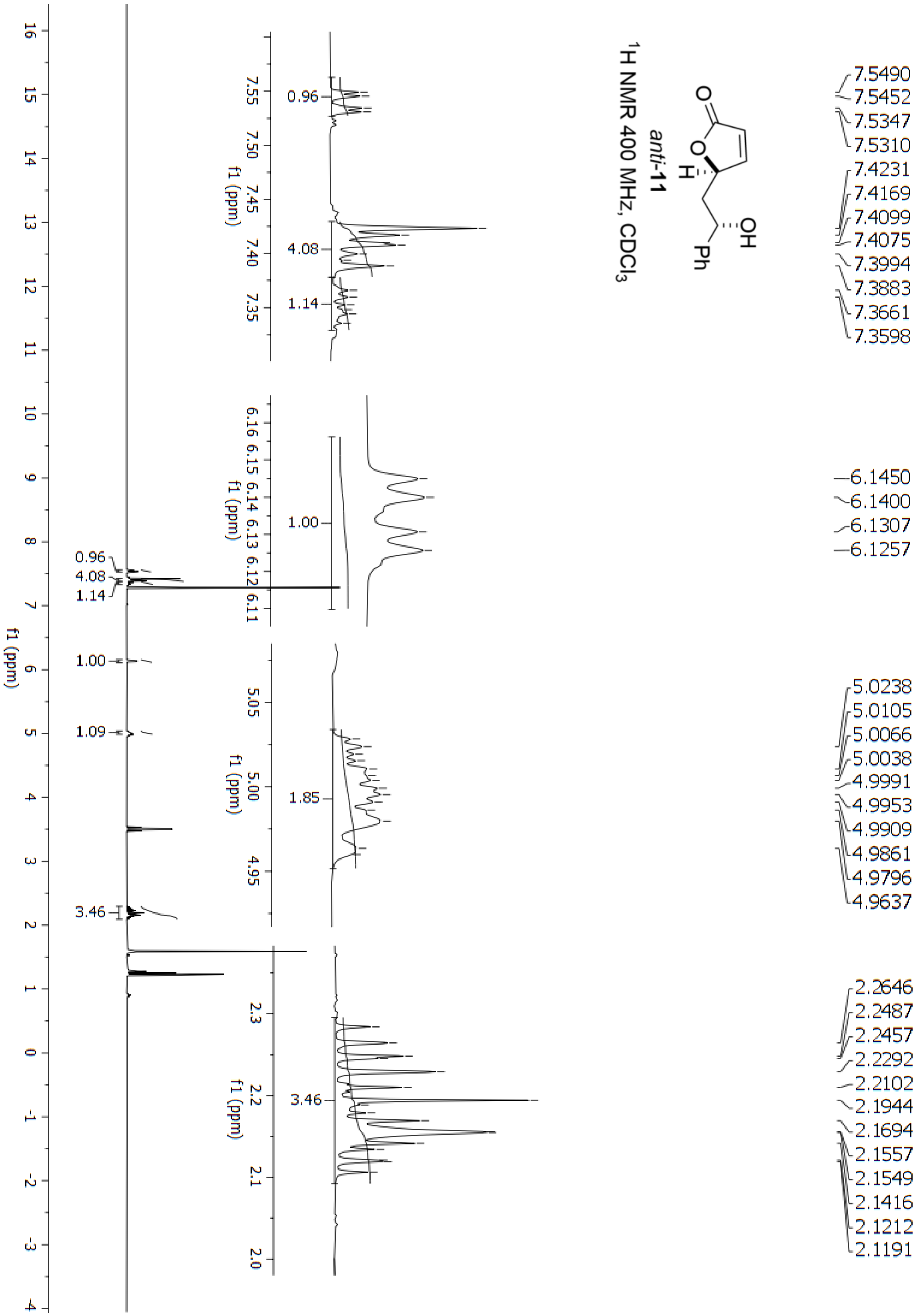
anti-11
1H NMR 400 MHz, CDCl3
OH
Ph
O
H
7.5490
7.5452
7.5347
7.5310
7.4231
7.4169
7.4099
7.4075
7.3994
7.3883
7.3661
7.3598
6.1450
6.1400
6.1307
6.1257
5.0238
5.0105
5.0066
5.0038
4.9991
4.9953
4.9909
4.9861
4.9796
4.9637
2.2646
2.2487
2.2457
2.2292
2.2102
2.1944
2.1694
2.1557
2.1549
2.1416
2.1212
2.1191
0.96
4.08
1.14
1.00
1.09
3.46
1.85
f1 (ppm)

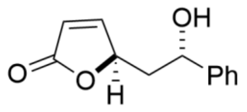


*anti*-**11**

$^{13}C$ DEPTq-135 NMR 101 MHz, $CDCl_3$

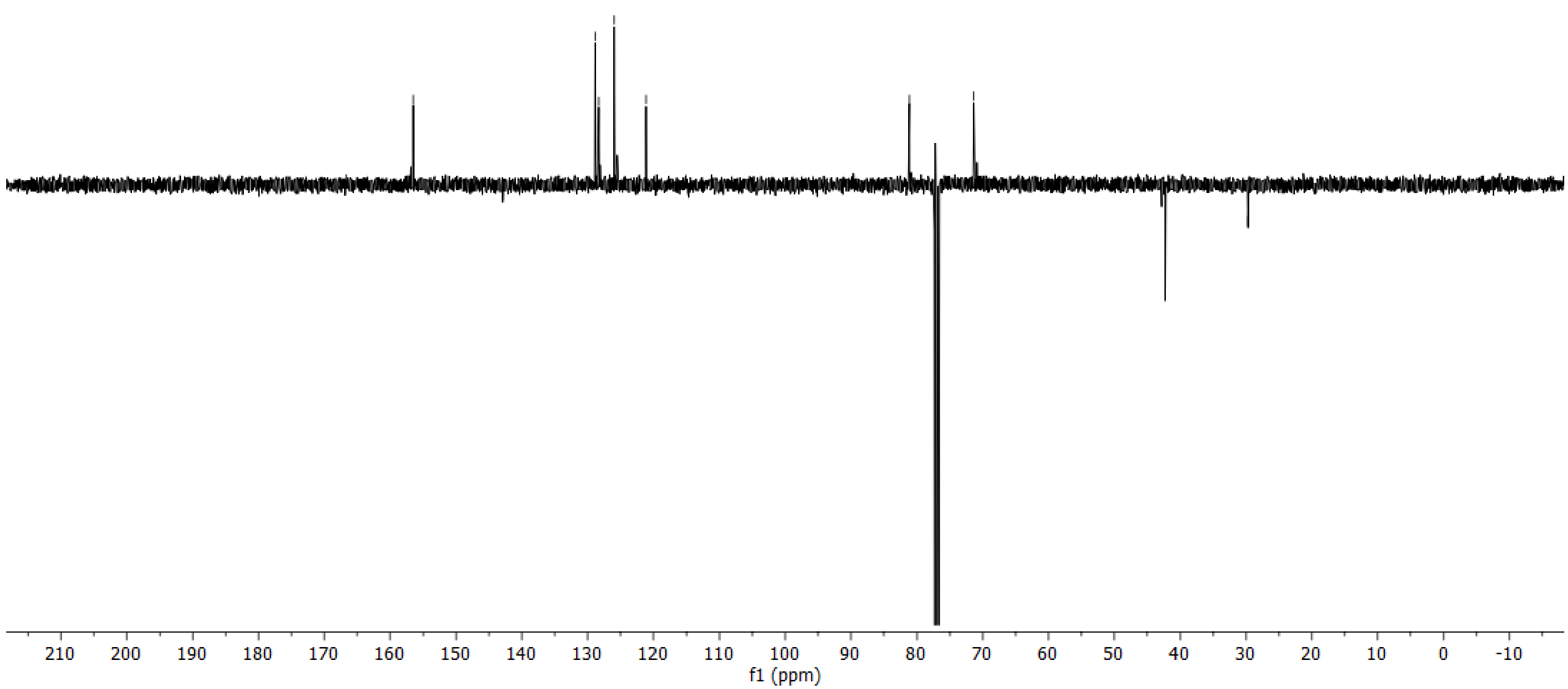

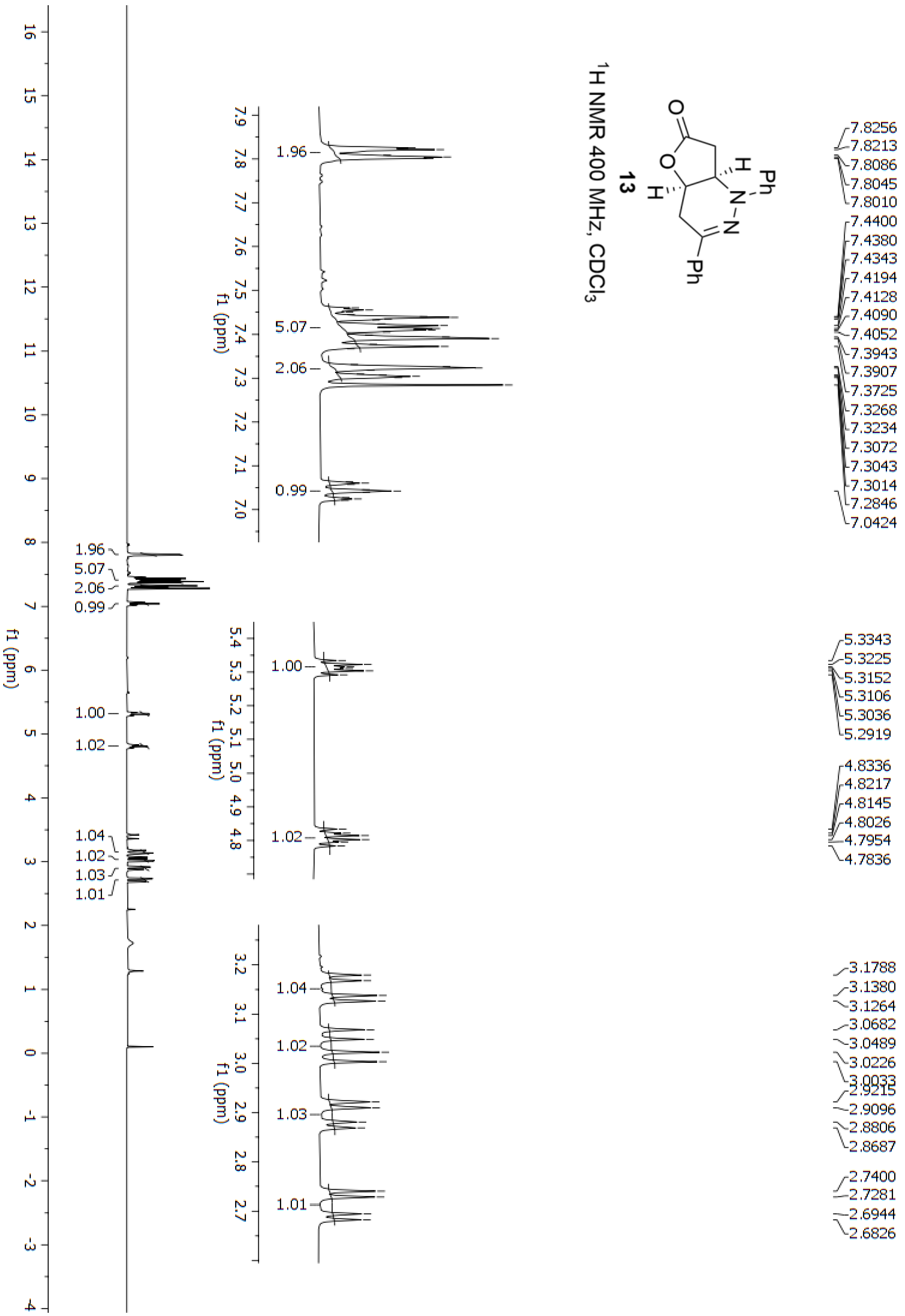
13
$^1$H NMR 400 MHz, $CDCl_3$
f1 (ppm)

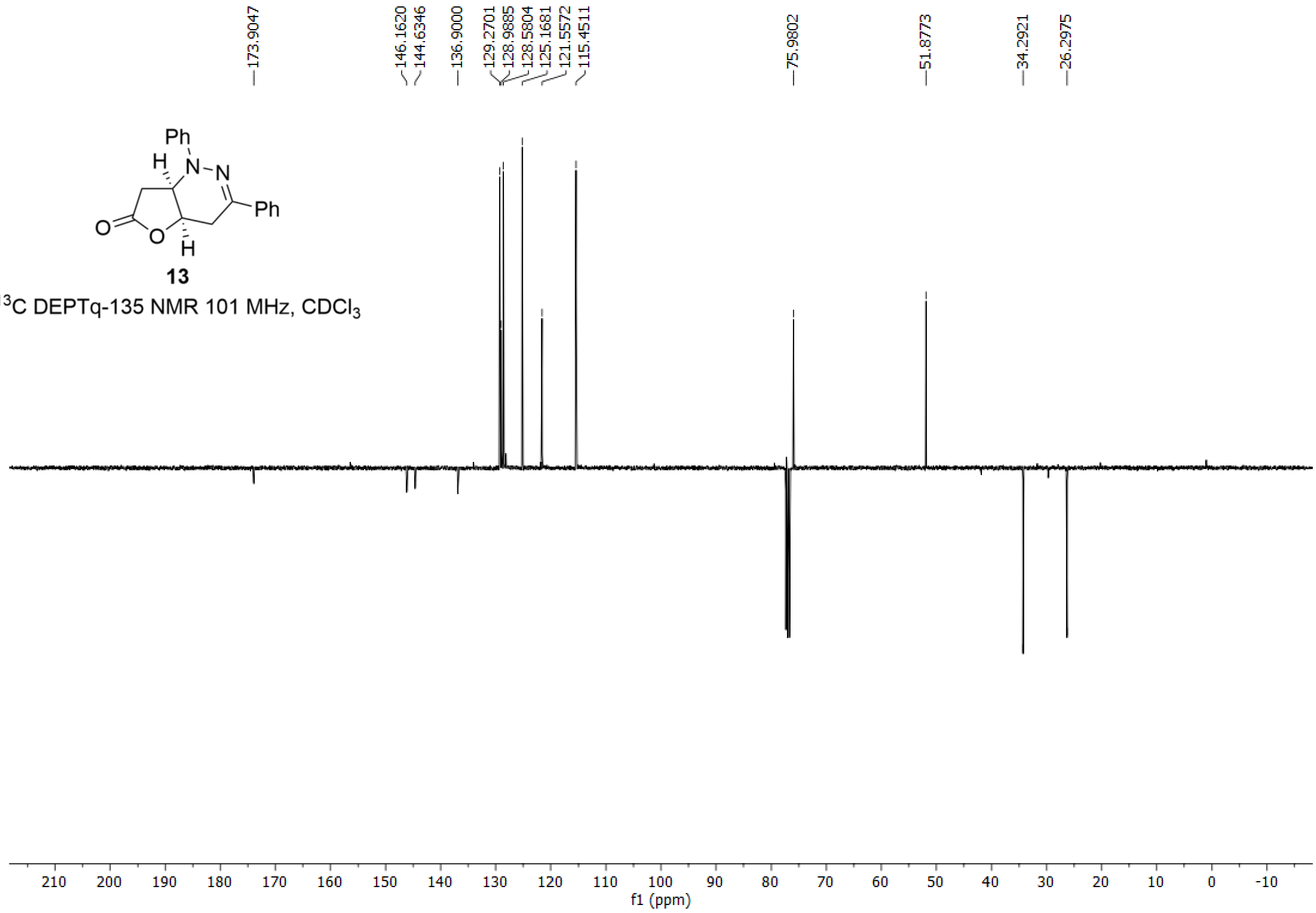


$^{13}$C DEPTq-135 NMR 101 MHz, $CDCl_3$
